# A core network in the SARS-CoV-2 nucleocapsid NTD mediates structural integrity and selective RNA-binding

**DOI:** 10.1038/s41467-024-55024-0

**Published:** 2024-12-09

**Authors:** Karthikeyan Dhamotharan, Sophie M. Korn, Anna Wacker, Matthias A. Becker, Sebastian Günther, Harald Schwalbe, Andreas Schlundt

**Affiliations:** 1 Institute for Molecular Biosciences, Goethe University, Frankfurt, Germany; 2 Center for Biomolecular Magnetic Resonance (BMRZ), Goethe University, Frankfurt, Germany; 3 Department of Biochemistry and Molecular Biophysics, Columbia University, New York, NY USA; 4 Institute for Organic Chemistry and Chemical Biology, Goethe University, Frankfurt, Germany; 5 Center for Free-Electron Laser Science CFEL, Deutsches Elektronen-Synchrotron DESY, Notkestr. 85, Hamburg, Germany; 6 Institute of Biochemistry, University of Greifswald, Greifswald, Germany

**Keywords:** X-ray crystallography, Solution-state NMR, RNA-binding proteins

## Abstract

The SARS-CoV-2 nucleocapsid protein is indispensable for viral RNA genome processing. Although the N-terminal domain (NTD) is suggested to mediate specific RNA-interactions, high-resolution structures with viral RNA are still lacking. Available hybrid structures of the NTD with ssRNA and dsRNA provide valuable insights; however, the precise mechanism of complex formation remains elusive. Similarly, the molecular impact of nucleocapsid NTD mutations that have emerged since 2019 has not yet been fully explored. Using crystallography and solution NMR, we investigate how NTD mutations influence structural integrity and RNA-binding. We find that both features rely on a core network of residues conserved in *Betacoronaviruses*, crucial for protein stability and communication among flexible loop-regions that facilitate RNA-recognition. Our comprehensive structural analysis demonstrates that contacts within this network guide selective RNA-interactions. We propose that the core network renders the NTD evolutionarily robust in stability and plasticity for its versatile RNA processing roles.

## Abstract (web summary)

The authors use solution-state NMR and X-ray crystallography to study mutations in the SARS-CoV-2 Nucleocapsid NTD, uncovering a network essential for protein integrity and the selectively of RNA binding.

## Introduction

The Covid-19 pandemic is widely considered as overcome, not least due to the global vaccination levels. Yet, the causative positive-sense (+) single-stranded RNA-virus *severe acute respiratory syndrome coronavirus 2* (SARS-CoV-2) continues to spread within the human population, though with seemingly less pathogenicity. Constant genomic mutation has resulted in variants of concern (VOC) with increased propagation, infectivity, or mortality^1^. VOCs harbor the omnipresent risk of re-emergence of highly pathogenic species. Unpredictable mutations may result in variants of yet unknown robustness and thus pose a major threat to humanity.

**Fig. 1:**
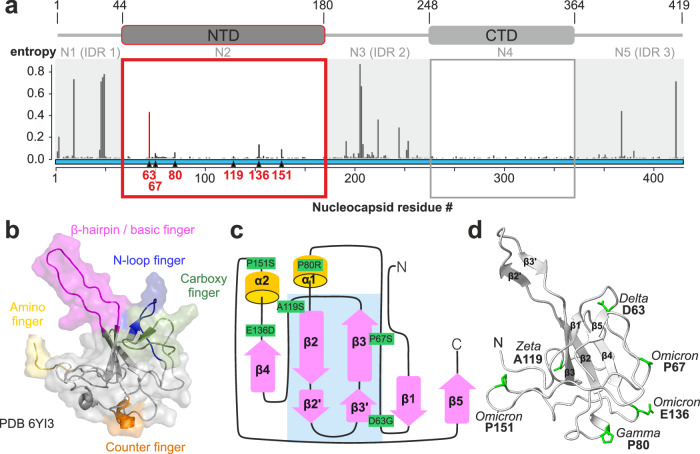
The SARS-CoV-2 nucleocapsid (N) RNA-binding domain NTD and its naturally occurring mutations (nat_mutants). **a** The N domain architecture including the folded NTD and CTD as indicated, flanked by IDRs. The amino acid numbering is given above, as well as an alternative nomenclature (N1-5). Mutations in the nucleocapsid coding sequence as of May 16, 2024, are depicted according to their respective normalized Shannon entropies^57,58^. Gray shades highlight IDRs, red and gray boxes the folded NTD and CTD, respectively. The red bar shows the entropy of residue 63 with the highest value inside the NTD (0.46). For the rationale of mutant selection, see the methods section. **b** NTD NMR structure (PDB 6YI3^6^) with color-coded flexible loop regions (fingers). **c** Scheme of secondary structure elements (α-helices shown as yellow cylinders, β-strands as purple arrows) shown for the NMR structure boundaries and localization of mutations in green. The primary RNA-binding interface is indicated by a blue box. **d** WT NTD crystal structure from this study. Residues, mutated in the indicated strains are highlighted in green stick representation (for a comparison with the NMR structure, see Supplementary Fig. 3c).

RNA viruses, such as those of the species SARS-CoV, rely on numerous viral-viral and viral-host RNA-protein interactions throughout their life cycle. One central protein involved in the formation of regulatory ribonucleoprotein complexes (RNP) is the coronaviral structural protein nucleocapsid (N). N plays a major role in RNA genome replication, translation, and packaging and has further been found to interfere with host-integrated stress responses and stress granule formation^1–3^. Its functions are indisputably based on selective interactions with viral and host RNA targets^4,5^. How N steers particular interactions relevant to the different functional requirements has remained incompletely understood. Recent studies have provided strong evidence for N’s folded RNA-binding domains (RBDs, Fig. 1a) to account for specific RNA-recognition, while its three extended intrinsically disordered regions (IDRs, N1, N3, and N5) are exploited for general affinity and necessary compaction of RNPs^6–9^ e.g., in the packaging of new viral particles.

The SARS-CoV-2 mutation rate is suggested to be 1 × 10^−6^ to 2 × 10^−^^6^ events per nucleotide per round of replication cycle^10^. A mutational hotspot, correlating with several VOCs, is the spike (S) protein required for host cell entry. Those mutations account e.g., for increased transmissibility^11^. Similarly, but less comprehensively correlated to distinct pathogenic characteristics, mutations in the N protein occur with high frequency^12,13^. Within the 419 amino acid long N protein, regions with increased mutational rates have manifested in several VOCs and cluster within the N-terminal and central IDRs, N1 and N3, respectively (Fig. 1a). Among them, R203(K/M) and G204R, located in the serine/arginine (SR)-rich region, are most prevalent and associated with an increased viral load and fitness^12–14^. In contrast, mutations in the N-terminal RBD (NTD, N2), which is reported as the driver for specific RNA-interactions^6,7,15^, are less frequent, but some are found to be lineage-defining (Fig. 1b–d). Mutations in the folded NTD will likely have a more complex impact on N functionality than those in the neighboring IDRs, and thus require our detailed examinations.

The N-NTD possesses a peculiar, hand-like three-dimensional fold, with a β-sheet palm and several flexible loops, arranged as fingers (Fig. 1b) around the central β-sheet^6,16,17^. The latter, together with the extraordinarily long basic β-hairpin finger, constitutes the positively charged primary RNA-binding surface. RNA complex formation has been suggested to rely on electrostatics and on stacking interactions mediated by highly conserved palm residues. Indeed, early studies on the model *Betacoronavirus* MHV have shown that mutations of residues R125 and Y127 (R107 and Y109 in SARS-CoV-2, respectively) result in loss of RNA-binding affinity and are lethal to the virus^18,19^. In the full-length (fl) context of multi-modular N, initial unspecific RNA engagement is mediated by the NTD in a mostly charge-driven manner^20^, supported by the IDRs that stabilize the newly formed RNP through high-affinity interactions. Indeed, a number of studies have shown the binding of NTD to non-viral model RNAs as proxys for single-stranded, double-stranded, and/or transiently structured motifs^6,7,21^. However, beyond non-specific interactions, the NTD is able to distinguish target RNA elements, such as the transcriptional-regulatory sequence (TRS) and packaging signals^7,15,19^. Preferences for viral RNAs are correlated with increased complex stability and NMR-observed signatures, both indicating that flexible loops are essential for specific RNP formation^7^.

The error-free distinction of RNA motifs seems to rely on an intricate correlation of finger motions, for which the domain exploits its intrinsic flexibility^6,7^. This multi-faceted interaction requires a conserved intramolecular network that acts in concert to scan RNA with respect to sequence, length, and fold, as well as to lock onto the right motifs. Despite achievements in modeling NTD RNPs as well as co-crystallization attempts^6,22,23^, the exact mechanism underlying NTD-RNA complexation remains hypothetical. We still face ambiguous information of how the NTD differentiates between RNAs, and what exactly allows specificity. It is thus not surprising that also the naturally occurring mutants (nat_mutants) within the NTD have remained largely uncharacterized with respect to their general influence on the NTD structural integrity as well as specific RNA-binding.

In this work, we investigate multiple mutations within the SARS-CoV-2 N-NTD in detail at the atomic level. We structurally and functionally characterize six prevalent naturally occurring NTD mutations that have either been categorized as lineage-defining for several VOCs or occur in Omicron VOC sub-lineages. Two of these nat_mutants show a slight increase in RNA affinity. We further provide evidence for an NTD core network originating from central residues Q58, W108, and F171, and responsible for NTD fold integrity. Disrupting the network at neuralgic sites, as proven by individual high-resolution structures, interferes with RNA-binding affinity and selectivity, which we unambiguously quantify and categorize with NMR spectroscopy and accessory biophysical techniques.

In sum, our data reveal the NTD structural and functional robustness relies on a distinct core network conserved among *Betacoronaviruses*. Our network hypothesis suggests that structural integrity and RNA-binding selectivity are intimately linked and offer an explanation for the lack of evolved mutations within the (expanded) network.

## Results

### The NTD 3D-fold is conserved in naturally occurring mutants

**Fig. 2:**
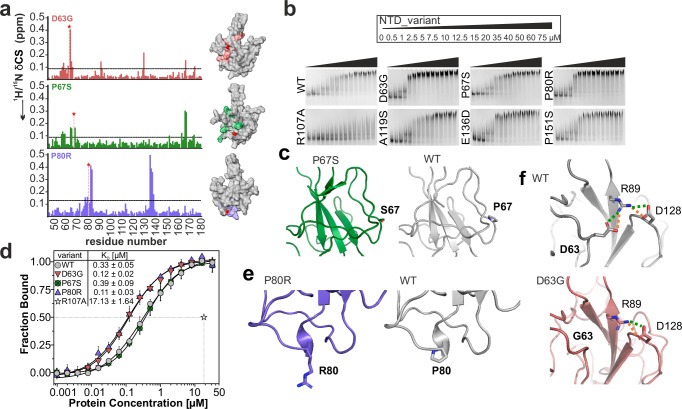
NTD nat_mutant 3D-folds are identical to WT. **a** ^1^H/^15^N CSD plots for NTD nat_mutants D63G, P67S, and P80R compared to the WT NTD, plotted over the amino acid sequence. Significant CSDs (average + 1SD, threshold indicated by dotted line) are mapped on the surface of the NTD NMR structure (PDB 6YI3^6^) in the respective color. The site of mutation is shown by a red sphere (Cα) and by a red star in the CSD plot. **b** EMSAs of NTD mutants with a described target RNA, SL4ext (Supplementary Fig. 2c). Protein concentrations are given above. Shown is a representative of two biological replicates (*N* = 2, see source data for quantification). **c** Zoom-ins of P67S and WT crystal structures showing the site of mutation. The zoom-in was set to comprise regions of significant CSDs according to panel (**a**) (see also Supplementary Fig. 2a). **d** Comparison of MST-derived *K*_D_ values of WT and three nat_mutants D63G, P67S and P80R for viral Ext RNA (3’-Cy5 labeled, see also source data). The transition point (50% bound) for RNA-binding deficient mutant R107A is indicated by dotted lines (see source data for the full curve). Data are presented as mean values +/− SD from three biological replicates (*N* = 3), each measured in duplicate. **e**, **f** Zoom-ins of the P80R and WT (**e**), and D63G and WT (**f**) crystal structures showing the site of mutation. The zoom-in area was set to comprise regions of significant CSDs according to panel (**a**) (see also Supplementary Fig. 2a). Contacts that are affected by the mutation are indicated (green – salt bridges, orange – H-bonds).

Along with multiple mutations in the SARS-CoV-2 N protein outside the structured domains, which cluster in the IDRs, several naturally occurring mutations (nat_mutants) have also emerged in the folded NTD (Fig. 1a, d). We selected NTD nat_mutants to compare them to the NTD from the Wuhan-Hu-1 N protein, further referred to as WT. Mutants were chosen based on their prevalence and/or categorization as lineage-defining for several VOCs listed on GISAID^24^, which remain the predominant nat_mutations (May 2024) (Fig. 1a, c and d and Supplementary Fig. 1a–c and “methods”). To reveal structural and possible functional consequences of the mutations described in Fig. 1, we initially analyzed the fold of mutant variants qualitatively in relation to WT using nuclear magnetic resonance (NMR) spectroscopy. We carried out extensive NMR backbone assignments and compared the ^1^H/^15^N HSQC (fingerprint) spectra for all nat_mutants with that of WT (Supplementary Table 1 and Supplementary Fig. 2). This allowed mapping of chemical shift differences (CSD) on the WT NMR structure (PDB 6YI3^6^). We found that nat_mutants could–as a proxy–be divided into two groups according to the distribution of CSDs: NTDs A119S, E136D and P151S show exclusively local effects induced by their respective mutations, while P67S, D63G and P80R display more pronounced and long-range CSDs (Fig. 2a and Supplementary Fig. 2a), suggesting those two groups of mutants could differentially modulate RNA-binding of the NTD.

We thus next probed RNA-binding of all nat_mutants for viral RNA-target SL4ext^7^ by electromobility shift assay (EMSA) (Fig. 2b). SL4ext is a described *cis*-regulatory element located in the 5’-untranslated region (UTR) of the genomic RNA, comprising the stable stem-loop (SL) 4^25^ and a 22 nucleotide extension (Ext)^26^, that transiently folds as a SL at physiological temperature (Supplementary Fig. 2c). We recently showed that the NTD preferentially binds to single-stranded Ext^7^, in line with its described preference for ss over dsRNA^6,21^. With the exception of D63G and P80R, all nat_mutants show WT-like binding (see source data for EMSA quantification), while previously described RNA-binding-impaired mutant R107A^6^ shows significantly reduced complex formation (Fig. 2b). In line with the unaltered RNA-binding behavior of these nat_mutants, we assumed a retained structure based on their NMR fingerprint spectra. Well-dispersed HSQC peak patterns overlaid largely with that of the WT spectrum, suggesting a merely local or less apparent impact of natural mutations on the NTD fold. For more detailed structural insight, we solved the high-resolution structures of all NTD nat_mutants and of the WT using X-ray crystallography (Fig. 2c, e and f, Table 1, Supplementary Tables 2 and 3 and Supplementary Fig. 3a). All crystal structures of nat_mutants with WT-like RNA-binding (nat_mutants P67S, A119S, E136D and P151S) superimpose well with the NTD WT with RMSD values between 0.592 and 1.776 Å (Table 1 and Supplementary Fig. 3a). Mutated residues in the above-named nat_mutants lie peripheral to the structural core (Fig. 1d), which further supports both the converging structures and the unaltered apparent RNA-binding affinities.

**Table 1:** Overview of crystal structures in this study with their PDB codes and respective specifications along with NMR assignment IDs. See Supplementary Table 3 for statistics and Supplementary Table 1 for the backbone assignment details

NTD_variant^a^	PDB code	Resolution in Å	RMSD^c^ in Å	Number of chains	Average chain RMSD^d^ in Å	Chain used for depiction^e^	BMRB ID^f^	Sequence coverage (% of all possible backbone amides)
NTD_WT	9EXB	2.30	–	4	0.581	C	34511^6^	–
NTD_D63G	9F83	1.70	0.668	4	0.476	D	52471	98.5
NTD_P67S	9EZB	1.60	1.047	4	0.667	A	52472	98.5
NTD_P80R	9F7A	1.90	0.898	1	–	A	52473	98.5
NTD_A119S	9F5L	2.36	0.592	4	0.399	B	n/a^g^	n/a^g^
NTD_E136D	9EVY	1.55	0.766	4	0.627	A	n/a^g^	n/a^g^
NTD_P151S	9FBG	2.54	1.776	16	0.775	H	n/a^g^	n/a^g^
NTD_Q58I^b^	9F5J	2.20	4.552	2	0.331	A	52469	98.5
NTD_S105I	9F7C	2.00	3.198	1	–	A	52474	94.2
NTD_Y109A	9EWH	1.93	0.674	4	0.727	C	52470	97.8

^a^NTD boundaries for X-ray crystallography span residues 41–174.

^b^NTD_Q58I was crystallized using a construct with boundaries from 44 to 180.

^c^RMSD for mutants with the WT crystal structure from this study; PDB 9EXB.

^d^Average RMSD for all chains within one asymmetric unit.

^e^See Method section for details on the selection of chain usage in figure panels.

^f^Backbone chemical shift assignments have been deposited at the BMRB for domain boundaries 44-180.

^g^NTD nat_mutants with only local CSD compared to WT. For details, see the Methods section.

### NTD natural mutants with increased RNA-binding affinity

Compared to the WT, the nat_mutants D63G and P80R appeared to form more distinct complex bands with SL4ext in the EMSAs (Fig. 2b). Interestingly, in contrast to the other nat_mutants, NMR-derived CSD plots for both mutants revealed more significant changes in comparison to the WT, suggesting a more far-reaching modulation of their fold or plasticity toward complex formation with RNA (Fig. 2a and Supplementary Fig. 2a). To determine whether this observation correlates with higher RNA-binding affinity we quantitatively compared *K*_D_ values of mutants and WT for the previously described target RNA Ext^7^ (Supplementary Fig. 2c) using microscale thermophoresis (MST). Interestingly, for this RNA sequence described as one of the prime N binding sites within the genomic 5’-UTR^7,26^, both NTD variants D63G and P80R show approximately 3-fold higher affinities than the WT (Fig. 2d), while–in line with the EMSAs–e.g., the P67S mutant shows no altered RNA-binding.

We further investigated the RNA-binding of D63G and P80R, located in the N-loop and counter finger, respectively, by NMR spectroscopy. The addition of 1.2 equivalents of Ext to nat_mutants yielded HSQC-observed chemical shift perturbation (CSP) patterns comparable to WT (Supplementary Fig. 4a). Yet, the comparison revealed no difference in binding interface or affinity, judged by CSP distribution and magnitude. We thus set out to solve the structures of both mutants for an atom-resolved explanation of increased RNA affinity. We were able to solve the 1.7 Å and 1.9 Å crystal structures for D63G and P80R, respectively (Fig. 2e, f). Surprisingly, both mutant structures superimposed with WT similarly well as the other nat_mutants, with RMSD values of 0.668 Å (D63G) and 0.898 Å (P80R), respectively (Table 1 and Supplementary Fig. 3a). Although the overall differences between the mutant and WT structures were insignificant, a closer look at the site of mutation at position 63 revealed the loss of salt-bridge/H-bond interactions between residues G63 and R89 (Fig. 2f) in our D63G structure. These subtle changes in intramolecular interactions possibly alter the flexibility of R89 sidechain and may lead to modulated RNA-binding properties of D63G (Supplementary Fig. 4b). In line with the retained backbone at this position in the crystal structures, {^1^H}^15^N heteronuclear steady-state NOE (hetNOE) values of backbone amides in the D63G mutant do not differ significantly from WT around the site of mutation (Supplementary Fig. 4c).

In sum, the tested nat_mutations largely resemble the WT NTD structure, but two nat_mutations showed a slightly increased affinity for RNA targets. Thus, our data stress the critical role of a conserved NTD fold for viral fitness that might account for an evolutionary advantage over other SARS-CoV-2 lineages.

### Conserved residues in the primary RNA-binding interface

**Fig. 3:**
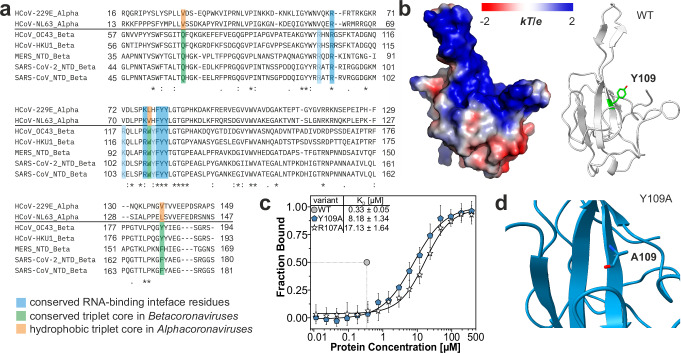
Mutation of Y109 within the RNA-interaction surface diminishes RNA-binding. **a** Alignment of NTD sequences from *Alpha*- and *Betacoronaviruses*, highlighting conserved residues of the RNA-binding interface (blue) and the triplet residues forming a core network (green for *Betacoronaviruses*, orange for the hydrophobic core in *Alphacoronavirus*). Sequences are taken from Uniprot^81^ entries Q6Q1R8 (HCoV-NL63), P15130 (HCoV-229E), K9N4V7 (MERS-CoV), P33469 (HCoV-OC43), Q5MQC6 (HCoV-HKU1), P59595 (SARS-CoV) and P0DTC9 (SARS-CoV-2), respectively. **b** Electrostatic surface potential of the WT. Y109–involved in RNA-binding–is located in the β-sheet core as visualized by the cartoon representation on the right. **c** Comparison of MST-derived *K*_D_ values for the two RNA-binding deficient mutants R107A and Y109A when titrated with viral Ext RNA (3’-Cy5 labeled). The transition point (50% bound) at the *K*_D_ of WT is indicated by dotted lines (see also source data). Data are presented as mean values +/− SD from three biological replicates (*N* = 3), each measured in duplicate. **d** Zoom-in on the site of mutation in our crystal structure of Y109A. The zoom-in area was set to include regions of significant CSDs from Supplementary Fig. 2b.

The limited number of evolutionary occurring mutations found within the N-NTD as compared to the neighboring IDRs (Fig. 1a) underlines a low tolerance in sequence deviation for maintaining the intricate NTD fold. Studies on the model *Betacoronavirus* MHV N-NTD have identified residues essential for RNA-binding, among them R125 and Y127^18,19^ (corresponding to R107 and Y109 in SARS-CoV-2, respectively). Two rationally designed, non-naturally occurring mutations (des_mutant) of palm residues R107 and Y109 in the SARS-CoV-2 N-NTD to alanine have subsequently been introduced as efficient RNA-binding impaired mutants early-on during the pandemic^6,17,26^. The primary NTD RNA-binding interface is conserved among *Betacoronaviruses*, revealing that RNA-binding is steered by the high density of positive charge and a central core of aromatic residues, among them Y109 (Fig. 3a, b)^16,27,28^. Mutations within this interface impact NTD RNA-binding^6,17^, while no experimental structure of such a mutant has been provided yet, which could report on the holistic effects of the exchange of critical amino acids. We determined *K*_D_ values for des_mutant Y109A by MST and found that it binds Ext RNA around 25-fold weaker compared to WT (Fig. 3c). Yet interestingly, it still binds Ext about two- to three-fold stronger than the R107A mutant (Supplementary Fig. 5). Considering a structure-based explanation, we solved the 1.93 Å crystal structure of Y109A (Fig. 3d, Table 1, and Supplementary Fig. 3b) and found that this des_mutation, similarly to the nat-mutants tested above, has no mentionable effect on the global NTD fold (RMSD 0.674 Å). Furthermore, NMR-derived backbone dynamics for the Y109A mutant compare well with those of WT suggesting the domain’s fold and intrinsic plasticity are unaltered (Supplementary Fig. 6). Altogether and in line with previous studies^21^, this study shows by high-resolution information that the tyrosine-to-alanine mutation directly interferes with RNA-binding, but not with the NTD fold integrity.

### Intradomain contacts vital for structure and RNA-binding

**Fig. 4:**
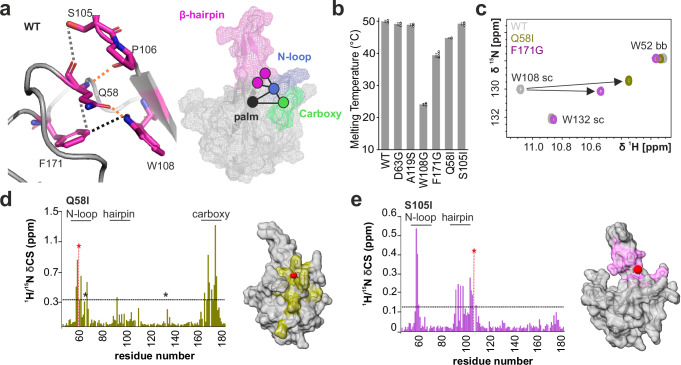
A core network is crucial for the structural integrity of the NTD. **a** Core network (purple)–as seen in our WT crystal structure–made up of triple residue contacts between Q58, W108 and F171, connecting the NTD N-loop, β-sheet palm and carboxy finger. The core network is expanded by contacts to the β-hairpin residues S105 and P106 (orange – H-bond; gray – vdW; black – vdW/π-π). Note that for the contact to S105, both H-bond and vdW contacts can occur within the four chains of PDB 9EXB. The F171-W108 contact has additional contributions from possible π-π interactions. **b** Melting temperatures for WT, the two nat_mutants D63G and A119S, and the four des_mutants of the network: W108G, F171G, Q58I and S105I. Data are presented as mean values +/− SD from three biological replicates (*N* = 3), each measured in duplicate. Individual replicates are shown as circles. **c** Spectral excerpt showing a comparison of ^1^H/^15^N-HSQCs for WT, Q58I and F171G, highlighting the tryptophan sidechain region. The extraordinary shift of W108 is indicated by the arrows. See Supplementary Fig. 2b and Supplementary Fig. 9 for a full spectral overlay. **d** ^1^H/^15^N CSD plots for Q58I and (**e**) S105I des_mutants vs WT plotted over the amino acid sequence. Significant CSDs (average + 1SD, threshold indicated by dotted line) are mapped on the surface of the NTD NMR structure (PDB 6YI3^6^) in the respective color. The site of mutation is shown by a red sphere (Cα) on the structure and by a red star in the CSD plot. Asterisks in the CSD plot indicate significantly line-broadened residues.

Our RNA-binding data for des_mutants R107A and Y109A confirm the role of both the electrostatic surface potential and central interface residues, likely involved in stacking interactions with RNA as suggested for MHV N-NTD^19^ and HCoV-OC43 N-NTD^27^. They do not, however, explain the capability of the NTD to distinguish RNA motifs for preferential interactions. Presumably, there is a more complex interplay of the rigid palm with the flexible fingers in the NTD that fine-tunes RNA-recognition. We have recently reported that mutation of serine 105, located at the interface of the β-hairpin and the N-loop, to isoleucine, results in more sophisticated changes in the NTD RNA-binding behavior^7^. Slightly more distant from the palm region, des_mutant S105I interferes with the ability of NTD to selectively recognize RNA target elements, likely caused by an impaired contact between β-hairpin residue S105 and the N-loop residue Q58. Located in the flexible N-loop finger, Q58 is positioned centrally to the core fold and seems crucial to a network connecting both the β-sheet palm and the flexible fingers: N-loop, β-hairpin and carboxy finger (Fig. 4a). In the heart of this network, a triplet of residues appears crucial for stabilizing the intramolecular connection between fingers and palm: Q58 (N-loop), W108 (palm) and F171 (carboxy finger) (Fig. 4a and Supplementary Fig. 7a). The core network is further expanded by contacts to neighboring regions, e.g., to the β-hairpin via the backbone of highly conserved P106. These conserved connections are supported by additional contacts to less conserved residues (such as S105). The core network averts the RNA-binding interface and is conserved among *Betacoronaviruses* (Fig. 3a). Interestingly, a similar network exists for *Alphacoronavirus* N-NTDs, however, composed of a hydrophobic triad establishing analogous contacts (e.g., V - V/L - L/V, Fig. 3a and Supplementary Fig. 8a).

To probe our network hypothesis, we designed mutations to study their influence on NTD architecture. We mutated tryptophan 108, the palm residue making contacts to Q58 and F171, to glycine, abrogating any potential sidechain interaction. Besides its obvious placement in the RNA-binding interface between the crucial RNA-binding residues R107 and Y109, we chose W108 as a site for mutation to interrogate its role in the SARS-CoV-2 N-NTD characteristic fold. Strikingly, the des_mutant W108G resulted in a highly unstable protein, indicated by a drastically reduced melting temperature (Tm) by more than 50 % compared to WT and the nat_mutants (Fig. 4b and Supplementary Fig. 7b). In agreement with its low Tm, W108G was prone to precipitation at room temperature further supported by NMR spectroscopy showing peak collapse into a narrow range of ^1^H chemical shifts, indicative of a loss of structural integrity (Supplementary Fig. 9a). Next, we mutated core network residue phenylalanine 171 to glycine. Comparable to W108G, F171G also showed a reduced Tm, indicating the mutation impact on NTD thermal stability. Of note, the HSQC overlay of F171G with WT revealed strong CSDs for residues located in the N-loop, the β-hairpin and the β-sheet palm (Supplementary Fig. 9b). The importance of the central core network is further supported by the mutation of residue Q58. To restrict its polar sidechain contact to W108 (Fig. 4a), we exchanged the bulky glutamine with a similarly sized, yet non-polar, isoleucine to maintain the local steric dimensions. Like the other core network mutations, Q58I had a reduced melting temperature (Fig. 4b). The lost contact with W108 in both des_mutants Q58I and F171G is clearly reflected in the extraordinary CSDs observed for the tryptophan 108 sidechain, contrasting the minor effects observed for other mutations (Fig. 4c, Supplementary Fig. 2d and Supplementary Fig. 9c).

Strikingly, in addition to the pronounced CSDs found for residues in the N-loop, hairpin, and carboxy regions (Fig. 4d), Q58I spectra showed significant line broadening for several backbone NH resonances (Supplementary Fig. 2b). This implies the destabilizing effect of the glutamine-to-isoleucine substitution is accompanied by enhanced conformational dynamics on the µs-timescale. Precisely, line broadening beyond detection was observed for Q58I residues 58, 64, 107, 109, and 131 (Supplementary Table 1, Supplementary Figs. 2b and 10a). While L64 and I131 form van-der-Waals (vdW) contacts to the core network (Supplementary Fig. 7a), R107 is located at the interface between N-loop and β-hairpin. We compared R_2_ relaxation rates of Q58I with WT and observed additional substantial contributions of µs-dynamics for residues Y111, W132, Y172, and A173 (Supplementary Fig. 10a). The destabilization originating from I58 can thus be tracked throughout an expanded network surrounding the core residues 58, 108, and 171.

In line with the notion of an expanded network, we also observed increased sub-ns motions within the Q58I N-loop and hairpin regions. {^1^H}^15^N hetNOE values for N-loop residues 58–63 decreased from 0.64 (WT average value, +/− 0.02) to 0.50 (+ /− 0.03) for Q58I, and from 0.52 (+ /− 0.01) to 0.44 (+ /− 0.01) for hairpin residues 90–105 (Supplementary Fig. 10b and Supplementary Table 4).

Comparing CSDs between des_mutants Q58I (core network) and S105I (expanded network) to the WT, respectively, shows that the Q58 mutation results in broadly dispersed chemical shift changes for residues in the N-loop, the β-hairpin, the carboxy finger and the palm (Fig. 4d and Supplementary Fig. 2b). In contrast, the S105 mutation exhibits more local affects, comprising the β-hairpin and the N-loop (Fig. 4e and Supplementary Fig. 2b), in line with the mutation site located outside the core network. Consequently, and different from Q58I, the melting temperature of S105I is not affected, but remains WT-like (Fig. 4b). In sum, our NMR-derived data, supported by biophysical analysis, highlights the importance of network residues Q58-W108-F171 for the NTD structural integrity.

### Impact of network mutations on structure and function

**Fig. 5:**
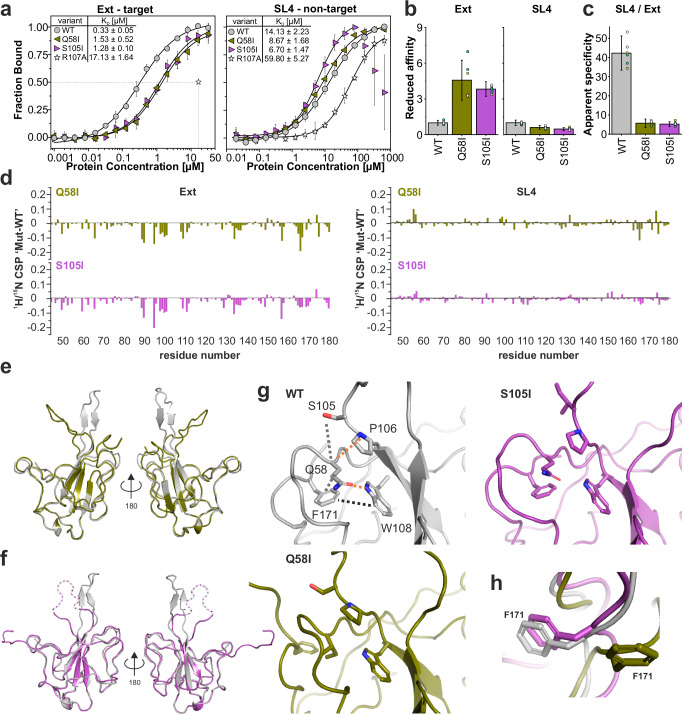
The core network is critical for NTD structural integrity and specific RNA-recognition. **a** Comparison of MST-derived *K*_D_ values of viral Ext and SL4 RNAs (3’-Cy5 labeled), respectively, when titrated with WT, the two des_mutants Q58I and S105I, or the negative control R107A. The transition point (50% bound) at the *K*_D_ of NTD_R107A to Ext RNA is shown by dotted lines. Data are presented as mean values +/− SD from three biological replicates (*N* = 3), each measured in technical duplicates. See Supplementary Fig. 11b and c for details on fitting S105I binding to SL4. **b** Reduced affinities indicated by fold-change of des_mutant *K*_D_ values from panel (**a**) normalized to the WT. Error bars represent standard errors propagated during normalization. Individual replicates are grouped by colored circles representing each biological replicate (yellow – N1, green – N2, and white – N3). **c** Apparent specificity was determined as the relative affinity increase for Ext RNA (target) versus SL4 RNA (non-target) by quantification of the fold-change of respective *K*_D_ values. A high value correlates with a strong preference for Ext over SL4. For error bars, see the explanation for panel (**b**). **d** Differential ^1^H/^15^N CSP plots (ΔΔδ) for Q58I and S105I in the presence of 1.2 equivalents Ext and SL4, respectively, obtained by subtraction from the corresponding WT CSP plot (see Supplementary Fig. 11a). **e**, **f** Superimpositions of Q58I and S105I with our WT crystal structure, respectively. For S105I, no consecutive electron density was obtained for hairpin residues from 94 to 102, and the loop was modeled with residual gaps (indicated by the dotted lines, see Supplementary Fig. 3b and d). **g** Zoom-ins on WT, Q58I, and S105I crystal structures highlighting the core network region. Residues and contacts (orange – H-bond; gray – vdW; black – vdW/π-π) are labeled in the WT structure only. **h** Superimposition of core network residue F171 in both mutants and the WT.

We next aimed to determine the role of the identified conserved network for NTD functionality. To this end, we analyzed the RNA-binding of des_mutants Q58I and S105I in more detail. Using MST, we determined their *K*_D_ values for the preferred viral RNA target Ext and the non-preferred viral stem-loop RNA SL4^7,25^ (Fig. 5a and Supplementary Fig. 2c). Compared to WT, Q58I and S105I show four to five-fold reduced binding to Ext (Fig. 5b). Intriguingly, no reduced RNA-binding is observed for the non-target SL4, in line with the model that SL4 is bound by NTD via electrostatic interaction, in a non-specific manner^7^ (Fig. 5b). The lost ability of both mutants to recognize a preferred RNA target is reflected by the relative decrease in affinity for Ext compared to SL4 (Fig. 5c). The apparent specificity is clearly expressed by a more than 40-fold increased affinity of WT for Ext over SL4, contrasting the merely small changes in binding observed in the two mutants. We further used NMR spectroscopy to probe mutant RNA interactions on a residue-resolved level (Fig. 5d). The general CSP patterns of Q58I and S105I upon interaction with 1.2 equivalents of Ext and SL4 RNAs, respectively, remain comparable to that of WT (Supplementary Fig. 11a). However, the CSP magnitude is significantly reduced for the complex with Ext, best visible in a differential CSP plot between mutants and WT (Fig. 5d). In contrast and in line with our MST-derived binding curves, differential plots for SL4-binding show no significant difference between the two mutants and the WT, respectively. In sum, these data confirm that Q58I and S105I are capable of binding to non-target RNA with a similar affinity as WT. Yet at the same time, the ability to recognize preferred RNA targets is strongly impaired.

To unravel the structural basis of network-disrupting mutations, we solved the 2.2 Å and 2.0 Å crystal structures of Q58I and S105I, respectively (Fig. 5e, f). The comparison of our WT structure to Q58I and S105I reveals a strongly deformed β-hairpin (basic finger) for both mutants (Fig. 5e, f, Table 1, and Supplementary Fig. 3b). This is reflected by high RMSD values of 4.552 Å and 3.198 Å for Q58I and S105I with the WT, respectively. Most importantly, while we find the β-hairpin architecture to be significantly altered in both mutants, the core network remains intact in S105I (Fig. 5g). This observation fits the WT-like melting temperature observed for S105I (Fig. 4b). In contrast to that, the Q58I crystal structure reveals a complete disruption of the core network Q58-W108-F171. Interestingly, phenylalanine 171 in Q58I is flipped out of its position, demonstrating the propagating effect of one single mutation within the core network (Fig. 5h). This influences the structural context of the carboxy-terminal stretch (171–174) in Q58I recapitulated in the Q58I R_2_ relaxation rates for residues 172 and 173, which contain significant contributions from conformational exchange (Supplementary Fig. 10a, c). This finding is consistent with our observation that NTD_Q58I only crystallized in a slightly extended C-terminal sequence context and, in fact, serves as a reasonable explanation for that behavior.

Collectively, our data from combined high-resolution X-ray crystallography, residue-resolved solution NMR experiments, and complementary biophysical methods indicate that the three-dimensional fold of the NTD of *Betacoronaviruses* depends on the conserved triple residue network (Q58-W108-F171), connecting the β-sheet core with the adjacent N- and C-terminal fingers. As derivable from the above, the postulated core network not only establishes the NTD fold integrity but also positions the flexible loops around the RNA-binding interface, thus enabling their coordinated interplay that appears critical for specific RNA-recognition.

## Discussion

Since the first described cases of SARS-CoV-2 infection in 2019, subsequent variants have evolved through genomic mutation from the origin^24^. So-called VOCs have the potential of being more transmissive or pathogenic, thus of concern, and are branded by specific lineage-defining mutations^29^. The N protein, crucial for every step in the viral life cycle, was found to carry several stable mutations in different variants, e.g., in its IDRs^14,30^. As shown also for other nucleic acid-binding proteins, IDRs are often associated with increased RNA-binding affinity^9,31^, and mutations likely affect binding strength. In contrast, it seems plausible that mutations in the SARS-CoV-2 N-NTD, which had been the focus of several studies in the context of immunogenicity and viral fitness^32,33^, can influence specific RNA-binding rather than just modulate affinity.

**Fig. 6:**
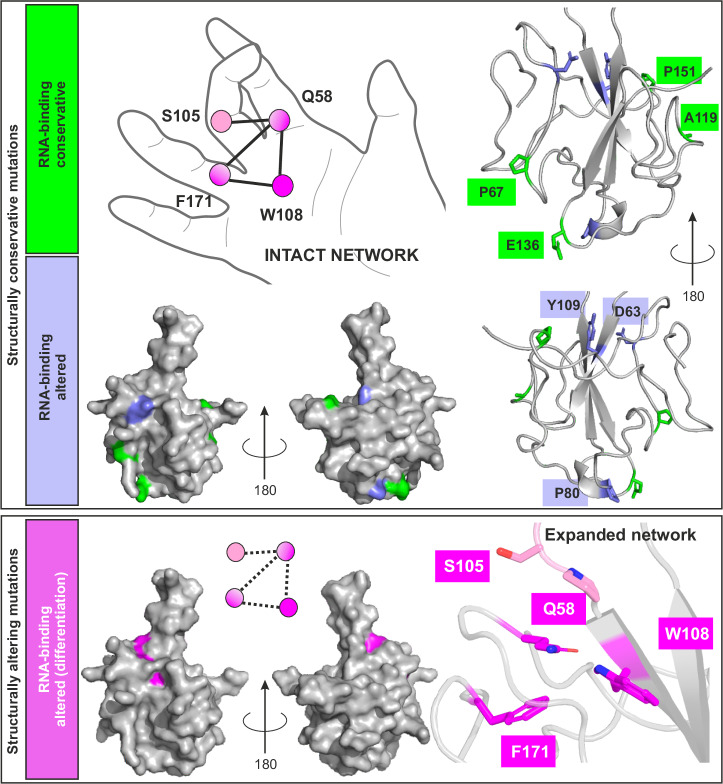
Summary of SARS-CoV-2 N-NTD mutations and their impact on NTD function and fold. Schematic depiction of the NTD hand-like fold highlighting the expanded network that connects the β-sheet palm (W108), the flexible carboxy finger (F171), the N-loop finger (Q58), and the basic finger (β-hairpin, S105). Herein characterized naturally occurring mutants (nat_mutants) within the NTD are conservative regarding the NTD fold and–except for D63G and P80R–show WT-like RNA-binding (green). The des_mutants R107A and Y109A, as well as nat_mutants D63G and P80R show generally changed RNA-binding affinities (decreased and increased, respectively) despite unchanged fold, in line with an unaffected core network (lilac). Mutations within the (expanded) network destabilize the domain fold (indicated by dotted lines in the schematic depiction) and prevent specific RNA-binding (magenta).

The SARS-CoV-2 N-NTD has an exceptional fold, reminiscent of a right hand, with a β-sheet core (palm) and flexible loops (fingers) as schematically presented in Fig. 6. The peculiar domain exhibits dynamics covering a broad range of timescales, is highly susceptible to pH and salt concentration, and described to bind bulk nucleic acids with a preference for labile folded AU-rich RNA elements^6,7,34,35^. However, a comprehensive understanding of the NTD RNA-binding mechanism is still missing, despite manifold large efforts to gain structural information on RNPs by our lab and others^6,22,23,36,37^. The extruding fingers, especially the large central β-hairpin, play a key role in the interaction with RNA^6,7,17^. The crosstalk between fingers, in combination with their intrinsic flexibility, is suggested to be essential for sensing specific target RNAs and forming stable complexes^7^. Similar concerted intradomain motions have been proposed for other RNA-binding proteins^38,39^, indicating it to be a more widespread mechanism for specific RNA-recognition^40^.

Despite numerous high-resolution structures of the NTD^6,16,17,41^, the underlying intramolecular loop interactions had only been investigated for SARS-CoV^42^. Further, the impact of naturally occurring NTD mutations on structure and RNA-binding competence had not been addressed comprehensively. While the effects of some NTD mutations have been analyzed in silico^43,44^, no broad experimental validation of N-NTD protein stability and RNA-binding has followed up on this.

We thus here specifically investigated the top six naturally occurring mutations in the NTD (three of them lineage-defining) as of September 2022. High-resolution crystal structures supported by solution NMR data reveal that all six nat_mutants are overall conservative regarding fold and functionality (Fig. 6). While this is in line with previously suggested minor mutant effects in e.g., D63G^45^, our study provides a systematic structure-driven analysis of all relevant natural NTD mutations. From those, only P80R and D63G exhibit slightly increased affinity for RNA, which likely correlates with an increased positive surface charge that possibly supports RNA engagement (Supplementary Fig. 4b). The effects are moderate and may be neglectable in the full-viral context, which is in line with earlier findings using P80R (and A119S) variants of N testing the infectiousness of virus-like SARS-CoV-2 particles, where no significant effect was observed^46^. However, we suggest that such mutations still may be more effective, when e.g., combined with other mutations and/or relevant changes in targeted RNA elements. Of note, the listed mutations might have a more relevant effect on the genomic level, e.g., by altering local RNA structure or stability. On the protein level, mutations D63G and P80R also impact the gene product of the overlapping shifted open reading frame ORF9b^47,48^, where they result in T60A and Q77E, respectively. In the context of N itself, these mutations may also have direct consequences for immune evasion of the virus and could e.g., alter epitopes detected by circulating antibodies. A recent study suggests P151 is part of such an epitope^49^. As our structure shows the NTD fold is unaltered in the P151S mutant, this suggests a respective virus variant may benefit from lowered detection by the immune system, and the same could be true for other loop-located mutations.

We further investigated the non-naturally occurring mutant Y109A, located in the primary RNA-binding interface (Fig. 6), and described it as an RNA-binding deficient mutation^17,19,26^. Interestingly, though widely used as a biological tool to mute NTD RNA-binding in a fl-N context^26,50^, no structural characterization of this des_mutant existed. We here solved the Y109A crystal structure and can show that structural integrity is retained in this mutant. Notably, while significantly reduced in affinity, the mutant still bound RNA via the primary RNA-binding interface (Supplementary Fig. 5b). Yet, mutation of close-by residue R107 had a much stronger effect on RNA-binding, underlining that Y109A should be considered as RNA-binding impaired rather than RNA-binding incapable in future studies.

The observed conservation of the NTD fold (Fig. 6) in nat_mutants and the Y109A des_mutant in the RNA-binding interface underlines the evolutionary robust nature of the N-NTD and its crucial role in genome processing. However, it seems a plausible scenario that mutations in the NTD may further co-evolve with mutations in regulatory target RNA-regions. We thus suggest to carefully follow the evolution of SARS-CoVs on the molecular level, including the currently neglected genome and proteome regions with possible roles in genome processing.

Early during the pandemic, residues beyond the primary RNA-interaction surface, located in the N-loop, β-hairpin, and the carboxy finger, had been suggested as relevant for NTD RNA-binding^51^, but a structural basis was not given. We here provide structural evidence for an expanded network, originating from a central core–Q58-W108-F171–that bridges the central β-sheet platform (W108) with the neighboring flexible N-loop (Q58) and carboxy finger (F171) and steers specific RNA-recognition (Fig. 6). Disruption of either the core or expanded network results in an NTD incapable of specific RNA-interaction. This supports earlier assumptions that the NTD engages with bulk RNA in a charge-driven, non-specific manner^7,20^, but senses and locks onto specific targets via the concerted interaction of finger motions.

Our crystal structures of Q58I and S105I show a strongly impaired arrangement of the extruding basic finger and a loss of its β-hairpin character (Fig. 5e, f). Both mutants lack the capacity to specifically recognize target RNA yet retain the general capacity to bind RNA non-specifically. Remarkably, the core network Q58-W108-F171 remains intact in S105I, with a local decoupling of the N-loop and the β-hairpin. This stands in contrast to Q58I, where destabilization of the core network is consistently reflected in reduced thermal stability. Equally, mutations of the core network residues W108 and F171 result in highly unstable proteins and a strongly altered 3-D fold as assumed by NMR spectroscopic analysis. Beyond W108 and F171 making π-π and vdW interactions in the SARS-CoV-2 N-NTD, F171 is fixed in position by hydrophobic contacts to neighboring residue L64. In Q58I, the conserved orientation of F171 is abolished, indirectly confirmed by NMR-observed line-broadening of L64 and elevated conformational exchange contributions for Y172 and A173 (Supplementary Fig. 10). We hypothesize that the interplay of flexible fingers in the domain is crucial for specific complex formation, allowing the NTD to grasp preferred targets. That concerted mechanism likely requires an intact network, e.g., to timely coordinate protein sidechain contacts with respective RNA bases or backbone, if in the correct sequence-encoded conformation, i.e., when considering recognition of RNA shape. Our hypothesis is well supported by the unaffected binding of S105I and Q58I to the non-target RNA SL4, while the affinity of S105I and Q58I to target RNA Ext is significantly reduced compared to the WT.

The crucial role of an intradomain network, mediating structural integrity and building the basis for an intricate crosstalk between flexible fingers is further seen in its evolutionary conservation. A comparison of available *Betacoronavirus* N-NTD crystal structures reveals the N-NTD core network to be highly conserved in the subgenus *Sarbecovirus* (SARS-*Betacoronavirus*). However, also in phylogenetically more distant *Betacoronavirus* species a comparable core network exists (Fig. 3a and Supplementary Fig. 8c). In line, the orientation of W108 and F171 is highly coordinated as shown by intra-residue RMSD values between 0.058 to 0.567 Å for these two residues over five different Betacoronavirus species structures (HCoV_43 PDB 4J3K^28^; MERS PDB 4UD1^52^; MHV PDB 3HD4^19^; SARS-CoV PDB 2OFZ^53^).

Interestingly, the *sarbecoviral* phenylalanine located in the NTD carboxy finger is replaced by a tyrosine residue in HCoV-OC43 and HCoV-HKU1, two representatives of the subgenus *Embecovirus*. The core network in both is established by H_2_O coordination between Q-W-Y (analogous to Q58-W108-F171 in SARS-CoV-2). Of note, in both *Embecovirus* NTDs, the orientation of the central tryptophan (W108 in SARS-CoV-2) is dictated by additional π-π interaction to a phenylalanine within the N-loop. This residue correlates to L64 in SARS-CoV-2, a residue we find strongly affected by our Q58I mutation.

Further comparison shows that a similar network exists in *Alphacoronaviruses*, yet the core residues form a hydrophobic cluster instead (Fig. 3a and Supplementary Fig. 8a). In the *Gammacoronavirus* IBV N-NTD, the core network is less pronounced in that it lacks sidechain contacts from the Q58 equivalent residue A41 (IBV N-NTD PDB 2BXX^54^), while the interaction between the network pair F153 and W91 (F171 and W108 in SARS-CoV-2) exists (Supplementary Fig. 8b). Of note, the IBV N-NTD was described to bind to its TRS with 50-fold lower affinity compared to the *Betacoronavirus* MHV N-NTD, suggesting the underdeveloped core network to correlate with that^18^. For any context, we envision that targeted disruption of coronaviral NTD core networks may, in the future be exploited in adding to inhibitor-based therapeutic cocktails silencing virus propagation. We and others have recently started to target the N-NTD with potential small-molecule inhibitors^23,55^, and the herein presented findings will help to rationally design more tailored compounds against the network.

Altogether, we show here that a combination of biochemical, biophysical, and spectroscopic solution methods with X-ray crystallography is suited to comprehensively describe the structural and dynamic features of the NTD required for function. We consistently demonstrate structure-function relationships unambiguously linking NTD RNA-binding to structural and dynamic prerequisites. We delineate–by a set of strategic mutations–that the conserved network steers RNA-binding via communication of the flexible fingers with each other and the palm. Such direct structure-function relationships are often obscured when either relying on low-resolution methods or when investigating N in a fl-context, occasionally leading to misinterpretation/overemphasis of effects that are rather a sum of several contributions than assignable to a single residue. A prominent example is the proclaimed loss of RNA-binding capability in Y109A that had been misinterpreted before, while we here–together with a recent study by Estelle et al.^21^–revise the effect of this mutant, facilitated by an atom-resolved view. The herein-composed data represent a valuable and comprehensive mutational analysis, providing detailed, high-resolution information that suggests a critical structural entity. We propose that its functional embedment in the context of N is relevant for specifically recognizing RNA targets.

## Methods

### Selection of NTD nat_mutants

Single nucleotide polymorphisms (SNP) that occurred within the domain boundaries of the SARS-CoV-2 nucleocapsid NTD (residues 44–180) and are either characterized as lineage-defining mutations, or as prevalent in the circulating VOC Omicron^56^ were chosen from GISAID^24^ (as of 9/22/2022). Lineage-defining mutants were chosen based on three criteria: 1) the total number of sequences carrying the mutation (at least 75,000 sequences carrying the mutation, except for A119S which has 19,272 sequences), 2) the ratio of mutant sequences to all sequences (minimum of 1 % cumulative prevalence), and 3) fraction of sequences carrying the mutation within lineages (minimum of 75 % mutational prevalence within lineage). Omicron-specific mutants were selected based on their appearance as SNP (following criteria 2 from above). Three of the herein investigated nat_mutations were lineage-defining: D63G specific to Delta (PANGO lineage B.1.617.2), P80R specific to Gamma (PANGO lineage P.1), and A119S Zeta (PANGO lineage P.2) variants of SARS-CoV-2. The other three mutations were prevalent mutations in Omicron: P67S (PANGO lineage BA.1.20), E136D (PANGO lineage BE.1.1) and P151S (PANGO lineage BA.4).

SARS-CoV-2 variants evolution focusing on the nucleocapsid protein was followed on nextstrain.org. For visualization, the nucleocapsid coding sequence–either as of September 22, 2022, or as of May 16, 2024–was depicted according to their respective normalized Shannon entropies^57^. In the context of proteins, the Shannon entropy is used to estimate mutational hotspots^58^. Plots showing mutation distribution were generated by graphical adaptation of a screenshot taken of the website nextstrain.org (URL: https://nextstrain.org/ncov/gisaid/global/all-time?c=gt-N_63).

### Construct design

The SARS-CoV-2 N-NTD coding sequence, defined as WT in this study, was based on NCBI reference genome entry NC_045512.2^59^. In this study, two different domain boundaries were chosen: (1) Boundaries of a first construct–referred to as NTD–were defined in analogy to the available NMR structure (PDB 6YI3^6^), spanning amino acids 44–180 and was cloned as described previously in detail by us ref. ^7^. (2) Based on literature^17^ and our own crystallization efforts, boundaries for NTD_xtal_ were chosen to span residues 41–174 (with the exception of Q58I, for which a crystal structure was solved for boundaries spanning residues 44–180). Comparison of the WT NMR structure (44–180) (PDB 6YI3^6^) with our WT crystal structure (41–174) shows both are in good agreement with an RMSD of 1.75 Å (see Supplementary Fig. 3c). The NTD_xtal_ coding sequence was amplified via PCR using xFW and xRV primers (see Table 2). The amplified PCR product was cloned into the pET-Trx1a vector with an N-terminal His_6_-Tag, a thioredoxin tag (Trx), and a tobacco etch virus (TEV) cleavage site via Gibson assembly^60^. The final protein sequence of NTD has one (G) and NTD_xtal_ two (GS) additional non-native amino acids at the N-terminus after TEV cleavage, respectively. Single amino acid mutations were either based on naturally occurring Omicron VOCs [P67S (BA.1.20), E136D (BE.1.1), P151S (BA.4)], derived from GISAID^24^ as of September 22, 2022, lineage-defining mutants [D63G (Delta), P80R (Gamma), A119S (Zeta)] or non-natural, design mutants (des_mutants) Q58I, S105I^7^, R107A, W108G, Y109A and F171G. All mutations, except R107A (only expressed with boundaries spanning residues 44–180), were introduced in both the NTD and the NTD_xtal_ background by site-directed mutagenesis with primers (Table 2) designed using the NEBaseChanger® webtool (https://nebasechanger.neb.com/). In brief, plasmids were amplified with the single point mutation introduced via PCR using Q5 DNA polymerase. The PCR product was treated with Polynucleotide kinase, T4 DNA ligase at 25 °C for 2 h followed by *Dpn*I digestion at 37 °C for 1 h. The resultant plasmids were transformed into *Escherichia coli* (*E. coli*) DH5α and individual colonies with the correct mutations of interest were identified by sequencing (Microsynth).

**Table 2:** List of DNA oligonucleotides used in this study

Name	Sequence (5’ - > 3’)	Usage
xFW	GGTCTCGAGAATCTTTATTTTCAGGGCAGCCGTCCACAAGGTCTCCCTAAC	Cloning of NTD_xtal__WT(Gibson assembly)
xRV	GTTAGCAGCCGGATCCCGACCCTTATTCTGCATAAAAGCCCTTTGGGAGCGTTGTGCCTTG	Cloning of NTD_xtal__WT(Gibson assembly)
D63G_FW	CAGCATGGCAAAGAAGGGTTGAAGTTTCCCCGGG	SDM Mutagenesisa
D63G_RV	GGTTAGGGCCGTGAACCAGG	SDM Mutagenesis^a^
P67S_FW	CAAAGAAGATTTGAAGTTTAGTCGGGGACAGGGGGTTC	SDM Mutagenesis^a^
P67S_RV	CCATGCTGGGTTAGGGCC	SDM Mutagenesis^a^
P80R_FW	GAACAGCTCGCGGGATGATCAAATTGG	SDM Mutagenesis^a^
P80R_RV	GTGTTGATCGGAACCCCCTG	SDM Mutagenesis^a^
A119S_FW	GTCCCGAATCAGGCCTGCCGTATG	SDM Mutagenesis^a^
A119S_RV	CAGTGCCCAGATAGTAAAAGTACCATC	SDM Mutagenesis^a^
E136D_FW	GTCGCAACCGACGGTGCGCTCAATAC	SDM Mutagenesis^a^
E136D_RV	CCAAATAATGCCGTCTTTATTAGCACC	SDM Mutagenesis^a^
P151S_FW	CACTCGCAACTCGGCGAATAACG	SDM Mutagenesis^a^
P151S_RV	CCAATGTGGTCCTTCGGGG	SDM Mutagenesis^a^
Q58I_FW	GTTCACGGCCCTAACCATTCATGGCAAAGAAGATTTGAAG	SDM Mutagenesis^a^
Q58I_RV	CAGGAAGCCGTGTTATTAGGAAGTCC	SDM Mutagenesis^a^
S105I_FW	GAAAGACTTAATTCCGCGATGGTAC	SDM Mutagenesis^a^
S105I_RV	ATTTTCCCATCGCCACCA	SDM Mutagenesis^a^
Y109A_FW	GAAAATGAAAGACTTAAGTCCGCGATGGGCGTTTTACTATCTGGGCACTG	SDM Mutagenesis^a^
Y109A_RV	CCATCGCCACCACGGATACG	SDM Mutagenesis^a^
R107A_FW	GACTTAAGTCCGGCGTGGTACTTTTACTATCTG	SDM Mutagenesis^a^
R107A_RV	CAGATAGTAAAAGTACCACGCCGGACTTAAGTC	SDM Mutagenesis^a^
W108G_FW	GAAAGACTTAAGTCCGCGAGGGTACTTTTACTATCTGGG	SDM Mutagenesis^a^
W108G_RV	ATTTTCCCATCGCCACCACGGATAC	SDM Mutagenesis^a^
F171G_FW	CCTGCCTAAAGGTGGTTATGCCGAAGGCTCCCG	SDM Mutagenesis^a^
F171G_RV	GTGGTACCCTGTGGCAGTTGCAG	SDM Mutagenesis^a^

^a^SDM = site-directed mutagenesis using primers designed with NEBaseChanger® following the protocol described in the methods section.

The rationale for choosing isoleucine as replacement of glutamine in des_mutant Q58I was to maintain a similarly sized sidechain but abolish any possible polar contacts. Glycine as replacement for tryptophan and phenylalanine in W108G and F171G, respectively, was chosen to fully avoid any sidechain-mediated contacts that might compensate for aromatic stacking interactions.

All plasmids used in this study are listed in the source data file.

### Protein production

Protein expression and purification were performed comparable to previous purifications^61^. Plasmids encoding NTD and NTD_xtal_, and mutants thereof, were transformed in *E. coli* BL21 (DE3) for protein expression. The cells were grown either in LB (unlabeled protein), in minimal M9 medium supplemented with ^15^NH_4_Cl (^15^N labeled protein), or M9 supplemented with ^15^NH_4_Cl and ^13^C glucose (^13^C and ^15^N labeled protein) at 37 °C and shaking at 120 rpm until an OD_600_ of 0.6–0.8 was reached. Protein expression was induced with 1 mM Isopropyl β-D-1-thiogalactopyranoside (IPTG), and the cultures were incubated at 25 °C and shaken at 80 rpm for 18 h. Cells were harvested by centrifugation at 4 °C and 6238 × *g* for 15 min and subsequently lysed by sonication in 50 mM Tris pH 8.0, 300 mM NaCl, and 2 mM β-mercaptoethanol supplemented with 310 µg of protease inhibitor mix G (SERVA) per liter of culture. The lysate was separated from cell debris by centrifugation at 58,545 × *g* at 4 °C for 30 min. The supernatant was loaded onto Nickel-NTA agarose beads for immobilized metal affinity chromatography (IMAC). The protein of interest was eluted at 300 mM imidazole and was dialyzed overnight at 4 °C to remove excess imidazole and with 1:25 (molar ratio) of TEV protease to cleave off the N-terminal tag. Unbound cleaved protein of interest from a second IMAC was subjected to an initial size exclusion chromatography (SEC) on a Superdex™ 75 HiLoad 16/600 column (Cytiva), ran at 4 °C in 25 mM KPi pH 6.5, 50 mM KCl. Subsequently, possible traces of co-purified RNases were removed from the concentrated protein by ion exchange chromatography on a 6 mL RESOURCE™ S (Cytiva) cation exchange chromatography (CEX) column. The CEX column was equilibrated with 25 mM KPi pH 6.5 with 50 mM KCl, and the protein of interest was eluted using a salt gradient from 50 mM to 500 mM KCl. The final sample was buffer adjusted to 25 mM KPi pH 6.5, 150 mM KCl, and concentrated by ultrafiltration. Protein samples for crystallization were purified in 20 mM Tris-HCl pH 8.0, 50 mM NaCl, and 1 mM DTT during SEC. For CEX, the column was equilibrated with the same buffer as SEC, and the protein of interest was eluted using a salt gradient from 50 mM to 500 mM NaCl. The final sample was buffer adjusted to 20 mM Tris-HCl pH 8.0, 50 mM NaCl, and 1 mM DTT.

### RNA Production and Cy5-labeling

Three SARS-CoV-2 viral RNAs from the 5’ genomic end were used in this study, which are SL4^25^ (residues 86–125 of the SARS-CoV-2 genome, elongated by two non-natural G-C base pairs, 5’-ggG UGU GGC UGU CAC UCG GCU GCA UGC UUA GUG CAC UCA CGC cc-3’)^62^, SL4ext (residues 83-149 of the SARS-CoV-2 genome, elongated by two non-natural 5’ G’s, 5’-ggU CUG UGU GGC UGU CAC UCG GCU GCA UGC UUA GUG CAC UCA CGC AGU AUA AUU AAU AAC UAA UUA CUG-3’) and Ext (residues 129-148 of the SARS-CoV-2 genome, elongated by two non-natural 5’ G’s 5’-ggA UAA UUA AUA ACU AAU UAC U-3’), further defined in detail in ref. ^7^ and Supplementary Fig. 2c. Unlabeled RNA was in vitro transcribed by in-house expressed T7 RNA polymerase and purified as follows: dsDNA templates, derived from linearizing plasmid-DNA with *Hind*III-HF^7^ (see source data), were used for preparative-scale (10–20 mL) transcription reactions (4 h at 37 °C) and RNA was precipitated with 2-propanol overnight at − 20 °C. RNAs were separated on denaturing polyacrylamide gels (12–16 %), visualized by UV shadowing, and eluted into 0.3 M NaOAc overnight. Subsequently, RNA was buffer-exchanged to the final experimental buffer.

3’-Cy5 labeled RNAs were either purchased from Horizon Discovery or labeled in-house as follows. RNAs were buffer-exchanged to 20 mM Tris-HCl, 50 mM NaCl, and 1 mM DTT at pH 8.0. Labeling was performed in 100 µL reaction volume containing 200 pmol RNA, 5x (for Ext) or 10x (for SL4) molar excess of pCp-Cy5 (Jena Biosciences), 35 units of T4 RNA Ligase 1 and 80 units of RNase inhibitor (NEB) at 18 °C overnight. Unincorporated pCp-Cy5 was removed by using the Oligo Clean and concentrator kit (Zymo Research) following the manufacturer’s protocol.

### Crystallization and data collection

Crystallization trials were performed in 96-well SWISSCI plates with 10 commercial screens by sitting drop vapor diffusion method. Crystals appeared after 1–7 days at 4 °C, 16 °C or 20 °C. Diffraction-quality crystals were obtained from further optimization of initial hits. Obtained crystals were cryo-protected in mother liquor and snap frozen at 100 K. Datasets were collected at EMBL P13 beamlines at the PETRA III storage ring of the DESY synchrotron^63^ and at the Swiss Light Source (SLS) on macromolecular crystallography beamline PXI-X06SA. Preprocessed unmerged datasets from autoPROC+STARANISO^64^ were further processed in CCP4cloud^65^. Phases were obtained by molecular replacement (MR) using Phaser^66^ with 7CDZ as the search model. SAD experimental phasing was performed with the Crank2^67^ automated experimental phasing pipeline for NTD_Q58I and NTD_S105I mutants, as phases obtained from MR were insufficient for complete model building. Structures were built using ModelCraft^68^ as an automatic model-building pipeline, optimized using PDB-REDO^69^, and refined in REFMAC^70^ and BUSTER^64^ with manual corrections in Coot^71^.

Except for NTD_P80R and NTD_S105I all structures contained more than one chain per crystal unit. For further analysis, comparison between each other and for depiction in figures we used the chains given in Table 1. A representative chain of WT, nat_mutants and des_mutants was selected by highest completeness and total quality of the model (according to PDB validation). All structure images in the figure panels have been created using Pymol version 2.5.5 (Schrödinger) and UCSF ChimeraX (v1.8)^72^.

Regarding the completeness of the structural models, we found differential electron densities for the NTD β-hairpin/basic finger. In line with earlier crystal structures of the SARS-CoV-2 NTD WT as well as the decreased convergence of this region in the NMR structure (PDB 6YI3^6^), the basic finger region showed significantly weaker electron densities than the remaining domain also for most mutants. Still, we were able to unambiguously model the full β-hairpin in at least one of the chains in all NTD variants including WT (4/4: WT, D63G, P67S, A119S, E136D, Y109A: 16/16: P151S) except for P80R (0/1, completely missing: 94–103) and S105I. For the latter (0/1), no consecutive density was found between 94 and 102, but individual residues of the loop still show unambiguous density (G99, K100), and we thus decided to indicate the loop in the model. Clearly, loop-bending relative to the WT, and similar to the loop conformation in the functionally related Q58I mutant (full density for β-hairpin residues seen in 1/2), is unambiguously present as summarized in Supplementary Fig. 3d.

Additional experimental details are provided in Supplementary Table 2. Data collection and structure refinement statistics are provided in Supplementary Table 3.

### Microscale thermophoresis

Microscale thermophoresis (MST) experiments were performed using a NanoTemper Monolith NT.115 instrument with samples in 25 mM KPi 50 mM KCl buffer at pH 6.5 supplemented with 0.02 % Tween-20. In a volume of 10 µL, 16 serial dilutions (1:1) were prepared from protein stocks of 64 µM or 768 µM (for R107A and Y109A) (for titration to Ext) and 1280 µM (for titration to SL4), respectively. Each dilution was mixed with 10 µL of 12 nM 3’-Cy5-labeled RNA and incubated at 25 °C for 30 min. The samples were centrifuged for 5 min at 10,600 × *g* and loaded onto Monolith standard capillaries (NanoTemper Technologies). Ext-Cy5 and SL4-Cy5 were excited with 50 % and 100 % LED power, respectively. In all experiments, initial fluorescence (pre-heat) was recorded for 5 s followed by 20 s heating with 20 % infra-red (IR) laser power. The IR laser was turned off, and the back diffusion was recorded for 5 s (post-heat). All data were analyzed using PALMIST with F_cold_ and F_hot_ regions defined between 2–3 s and 5.5–6 s, respectively^73^. Representative data, shown as fraction bound, was obtained by normalization to response amplitude after baseline correction, with error values corresponding to standard deviations between three biological replicates (*N* = 3), each measured as a technical duplicate (see source data). Final curves were plotted in OriginPro.

Reduced affinities of NTD mutants for SL4 and EXT RNAs, respectively, were obtained from the ratio of their mean *K*_D_ values and the mean *K*_D_ value of the WT. The apparent RNA specificity of individual NTD versions was calculated by dividing their mean *K*_D_ values for SL4 by the respective mean *K*_D_ values for EXT. Within these two procedures, errors based on replicates (see above) were treated as follows: A fractional error (FE) of MST-derived affinities was calculated by dividing the standard deviation over all replicates by the mean *K*_D_ value. The final standard errors (SE), given for the reduced affinity of an NTD mutant as well as the apparent affinity of an NTD version, were propagated from fractional errors using the following equations, respectively:

1 $$	{{{\rm{SE}}}}({{{\rm{reduced}}}}\; {{{\rm{affinity}}}}\; {{{\rm{of}}}}\; {{{\rm{mutant}}}}) \\ 	=\left({{{\rm{Mean}}}}\; {{{\rm{red}}}}.{{{\rm{affinity}}}}({{{\rm{mutant}}}})*\left(\root 2 \of{{\left({{{{\rm{FE}}}}}_{{{{\rm{mutant}}}}}\right)}^{2}+{\left({{{{\rm{FE}}}}}_{{{{\rm{WT}}}}}\right)}^{2}}\right)\right)$$

2 $$	{{{\rm{SE}}}}({{{\rm{apparent}}}}\; {{{\rm{affinity}}}}\; {{{\rm{of}}}}\; {{{\rm{version}}}}) \\ 	=\left({{{\rm{App}}}}.{{{\rm{specificity}}}}({{{\rm{version}}}})*\left(\root 2 \of{{\left({{{{\rm{FE}}}}}_{{{{\rm{SL}}}}4}\right)}^{2}+{\left({{{{\rm{FE}}}}}_{{{{\rm{EXT}}}}}\right)}^{2}}\right)\right)$$

### NMR

NMR measurements were carried out at the Frankfurt BMRZ on Bruker spectrometers of 600, 700, and 1.2 GHz proton Larmor frequency, equipped with cryogenic probes and Z-axis pulsed field gradients. All NMR spectra of protein alone and in complex with RNA were recorded in 25 mM KPi, 150 mM KCl, pH 6.5, and 5 % D_2_O at 298 K and referenced with respect to external DSS^74,75^. Topspin versions 3 and 4 were used for data acquisition and processing. Backbone assignment and analysis of CSPs/CSDs and relaxation data were performed using the CCPNMR analysis 2.5 and 3.2 software suite^76^. Relaxation experiments were performed with 750 µM ^15^N labeled sample at 600 MHz (proton Larmor frequency) and 298 K. Both {^1^H}^15^N heteronuclear steady-state NOE (hetNOE) and R_2_ experiments were recorded as interleaved HSQC-based pseudo-3D versions including temperature compensation^77^ using standard Bruker pulse sequences (hsqcnoef3gpwg3d and hsqct2etf3gptcwg3d, respectively) Spectral widths were 16 ppm in the ^1^H dimension and 36 ppm in the ^15^N dimension. The ^15^N carrier was set to 117 ppm and ^15^N decoupling during acquisition was achieved with the garp4 pulse train at 3.6 kHz. hetNOE experiments^78^ were recorded with 2048–4096 and 128–144 complex points in the ^1^H and ^15^N dimensions, respectively, with 24–32 scans and a saturation delay of 6 s. R_2_ relaxation data^79^ were acquired with 2048 and 128 complex points in the ^1^H and ^15^N dimensions, respectively, and 48–80 scans, employing the following T2-delays: 16.96, 33.92, 50.88, 67.84, 101.76, 135.68 169.6, 203.52, and 271.36 ms. Inter-scan delays were set to 2 s. 3D ^1^H,^15^N NOESY-HSQC experiments were recorded at 298 K for 750 µM sample at 1.2 GHz (proton Larmor frequency) using the standard Bruker pulse sequence noesyhsqcf3gpsi3d with 2048 × 80 x 96 complex points in the (direct) ^1^H, (indirect) ^15^N and (indirect) ^1^H dimension, respectively. Spectral widths were 16 ppm for both ^1^H dimensions and 36 ppm for the ^15^N dimension. The ^15^N carrier frequency was set to 117 ppm and 16 scans with 100 ms mixing time and 1 s inter-scan delay were recorded. For RNA to protein titrations, we added 84 µM RNA to 70 µM apo NTD sample to the final titration point (1.2-fold molar excess). Combined ^1^H/^15^N-chemical shift perturbations (CSP) or differences (CSD) were calculated in ppm according to Eq. (3):

3 $${{{\rm{CSP}}}}/{{{\rm{CSD}}}}=\,\sqrt{{\left(\frac{{{{\rm{\delta }}}}{{{\rm{N}}}}}{5}\right)}^{2}+{\left({{{\rm{\delta }}}}{{{\rm{H}}}}\right)}^{2}}$$

Errors of hetNOE experiments were calculated according to Eq. (4):

4 $${{{{\rm{Error}}}}}_{{{{\rm{hetNOE}}}}}=\,\left({{{{\rm{I}}}}}_{1}/{{{{\rm{I}}}}}_{2}\right)*\left(\sqrt{{{\left({{{\rm{S}}}}/{{{\rm{N}}}}\right)}_{1}}^{-2}+{{\left({{{\rm{S}}}}/{{{\rm{N}}}}\right)}_{2}}^{-2}}\right)$$

where I_1_ is the intensity of the saturated peak, I_2_ is the intensity of the unsaturated peak, (S/N)_1_ is the signal-to-noise ratio for the saturated peak, and (S/N)_2_ is the signal-to-noise ratio for the unsaturated peak. The backbone ^1^H, ^13^C and ^15^N resonance assignments of SARS-CoV-2 nucleocapsid NTD nat_mutants (D63G, P67S, P80R) and des_mutants (Q58I, S105I, Y109A) were performed with 750 µM samples by analyzing ^1^H-^15^N-HSQC and the triple resonance experiments listed in Supplementary Table 1. In addition, for Q58I, sidechain ^1^Hε-^15^Nε (glutamine residues), ^1^Hδ-^15^Nδ (asparagine residues), and ^1^Hε-^15^Nε (tryptophan residues) were assigned, in parts supported by a ^15^N-NOESY experiment. For D63G, P67S, P80R, S105I, Y109A, and F171G, sidechain assignments of ^1^Hε-^15^Nε (tryptophan residues) were transferred from WT (BMRB 34511^6^) based on ^1^H,^15^N chemical shift similarity. The backbone ^1^H, ^15^N resonance assignments of SARS-CoV-2 nucleocapsid NTD nat_mutants (A119S, E136D, P151S) from 140 µM samples were transferred from WT (BMRB 34511^6^) based on ^1^H, ^15^N chemical shift similarity.

### EMSA

Qualitative EMSAs were performed with unlabeled SL4ext RNA and varying concentrations of protein in 25 mM KPi pH 6.5 150 mM KCl buffer. In a total volume of 10 µL, 15 dilutions of proteins in the range of 0–75 µM were prepared, to which 3 µM of SL4ext RNA was added and the samples were incubated at room temperature for 20 min. RNA-Protein complexes were resolved from free RNA by native polyacrylamide gel electrophoresis (1x TB, 6 % acrylamide/bis-acrylamide 37.5:1, 10 % glycerol). 3 µL of native RNA loading dye (1x TB, 60 % Glycerol, 0.02 % bromophenol blue) was added, and the samples were loaded onto the gel and ran for 60 min in 1x TB running buffer at 80 V. The gels were stained in 0.0005 % ethidium bromide solution for 10 min before being visualized on UV-28 ME UV transilluminator and analyzed on Herolab E.A.S.Y.429 K (Herolab GmbH, Germany).

For fluorescent EMSAs, 3’-Cy5 labeled SL4 was resuspended in 25 mM KPi, 50 mM KCl buffer at pH 6.5 supplemented with 0.02 % Tween-20. In a volume of 5 µL, 14 serial dilutions (1:1) were prepared from 1280 µM protein stocks. Each dilution was mixed with 5 µL of 12 nM Cy5-labeled SL4 and incubated at 25 °C for 30 min. RNA-Protein complexes were resolved from free RNA as described above, and gels were imaged using the Cy5 channel in a Bio-Rad ChemiDoc™ imaging system.

### Nano differential scanning fluorimetry

Thermal stability of SARS-CoV-2 NTD and mutants was characterized using nano differential scanning fluorimetry (nanoDSF) with a Prometheus Panta (NanoTemper Technologies) instrument. 12.5 µM samples in 25 mM KPi, 50 mM KCl buffer at pH 6.5 supplemented with 0.02 % Tween-20 were loaded onto Prometheus standard capillaries by capillary action. Changes in intrinsic fluorescence of tryptophan residues upon thermal unfolding of samples from 15 °C to 95 °C in 1 °C/min steps were recorded at 330 nm and 350 nm upon excitation at 280 nm. Samples were measured in three biological replicates (*N* = 3), each as a technical duplicate. The fluorescence signal at 330 nm as a function of temperature was analyzed using the MoltenProt web server, and the protein unfolding temperature (Tm) was obtained by fitting the raw data to an equilibrium two-state model^80^.

### Reporting summary

Further information on research design is available in the Nature Portfolio Reporting Summary linked to this article.

## Supplementary information

Supplementary Information

Reporting Summary

Transparent Peer Review file

## Source data

Source Data

## Data Availability

NMR spectral resonance assignments of this study use the following previously published entry for the WT SARS-CoV-2 N-NTD in the BMRB under the accession number BMRB 34511. The ^1^H, ^13^C, and ^15^N backbone chemical shift assignments of NTD mutants (nat_mutants and des_mutants) have been deposited in the BMRB under the following accession numbers: BMRB 52469 (NTD_Q58I), BMRB 52471 (NTD_D63G), BMRB 52472 (NTD_P67S), BMRB 52473 (NTD_P80R), BMRB 52474 (NTD_S105I), and BMRB 52470 (NTD_Y109A). The crystal structures presented in this study have been deposited in the PDB: 9EXB (NTD_WT), 9F83 (NTD_D63G), 9EZB (NTD_P67S), 9F7A (NTD_P80R), 9F5L (NTD_A119S), 9EVY (NTD_E136D), 9FBG (NTD_P151S), 9F5J (NTD_Q58I), 9F7C (NTD_S105I), and 9EWH (NTD_Y109A). All NMR spectra presented in this study will be provided upon request. In addition, we used NTD structures in this study that are available through PDB entries with the following accession codes: 6YI3 (NMR structure), 6M3M (crystal structure), 7CDZ (crystal structure), 5N4K (HCoV-NL63 NTD), 2BXX (IBV NTD), 4J3K (HCoV-OC43), 4UD1 (MERS-CoV NTD), 3HD4 (MHV NTD), and 2OFZ (SARS-CoV NTD). Material requests shall be made to the corresponding authors. Source data are provided in this paper.
