# Supplementary material for: A core network in the SARS-CoV-2 nucleocapsid NTD mediates structural integrity and selective RNA-binding
Source: Nat Commun. 2024 Dec 9;15:10656. doi: 10.1038/s41467-024-55024-0 (PMC11628620; doi:10.1038/s41467-024-55024-0)
Supplement: Supplementary file 1 — Supplementary Information [file 41467_2024_55024_MOESM1_ESM.pdf]

Supplementary Information

A Core Network in the SARS-CoV-2 Nucleocapsid NTD Mediates Structural Integrity and Selective RNA-binding

Karthikeyan Dhamotharan[1,2#], Sophie M. Korn[1,2,3#*], Anna Wacker[2,4], Matthias A. Becker[2,4], Sebastian Günther[5], Harald Schwalbe[2,4], and Andreas Schlundt[1,2,6*]

[1]Institute for Molecular Biosciences, Goethe University, Max-von-Laue-Str. 9, 60438 Frankfurt, Germany

[2]Center for Biomolecular Magnetic Resonance (BMRZ), Goethe University, Frankfurt/M., Germany

[3]Columbia University, Department of Biochemistry and Molecular Biophysics, 701W 168th Street, 10032 New York, NY, United States

[4]Institute for Organic Chemistry and Chemical Biology, Goethe University, Frankfurt/M., Germany

[5]Center for Free-Electron Laser Science CFEL, Deutsches Elektronen-Synchrotron DESY, Notkestr. 85, 22607, Hamburg, Germany

[6]University of Greifswald, Institute of Biochemistry, Felix-Hausdorff-Str. 4, 17489 Greifswald, Germany.

[#]These authors contributed equally.

*Correspondence. **Email:** smk2305@cumc.columbia.edu, andreas.schlundt@uni-greifswald.de

This document contains:

- 11 Supplementary Figures
- 4 Supplementary Tables
- Supplementary References

Supplementary Fig. 1

a

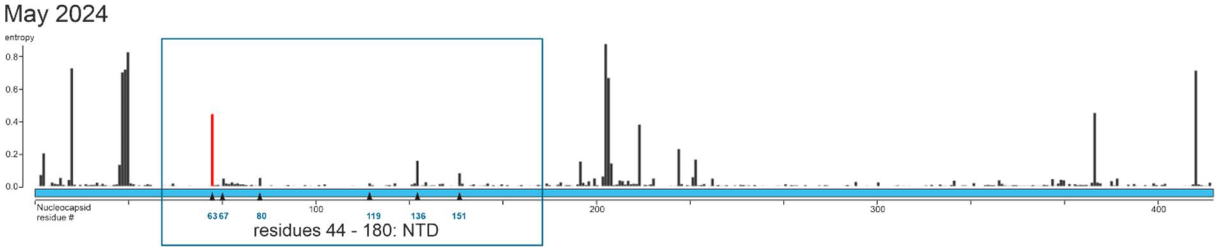

b

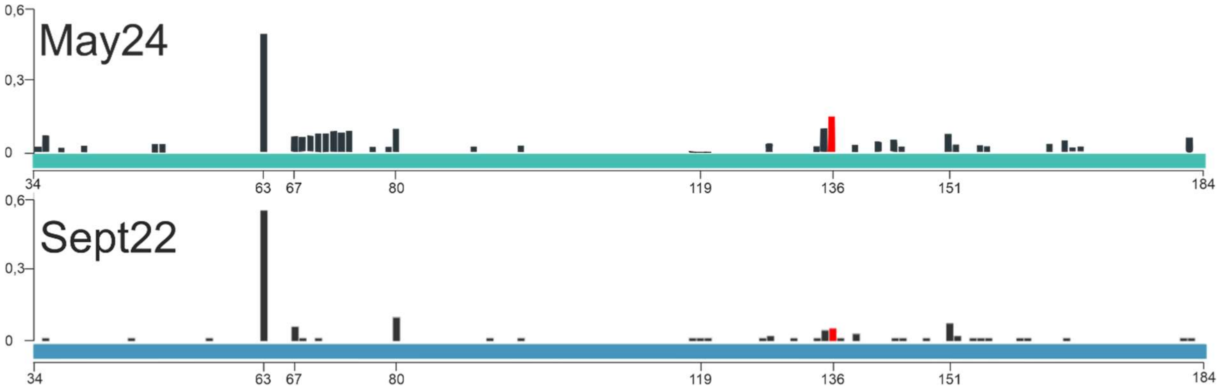

c

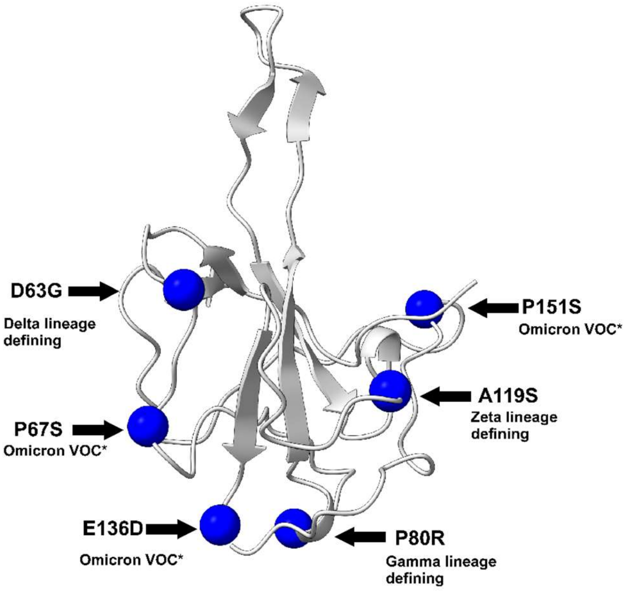

Supplementary Fig. 1: Naturally occurring mutants in the SARS-CoV-2 Nucleocapsid NTD (boundaries aa 44-180). a) Mutations in the Nucleocapsid coding sequence (codon sequence: x axis) are depicted according to their respective normalized Shannon entropies (y axis). The blue box comprises codons 44-180, corresponding to the NTD. The red bar shows the entropy of codon 63 representing the highest value inside the NTD (0.46). See methods for details. **b)** Comparison of Shannon entropies (y axes) for mutations in the NTD coding sequence (codon sequence: x axis), calculated for May 2024 (top) versus September 2022 (bottom). The red bars show the entropy of codon 136, corresponding to the E136D mutation, at the time points of 9/22/2022 (0.05) and 5/16/2024 (0.13). **c)** Shown on our WT_NTD crystal structure in spheres are Cα atoms in respective sites of mutations. The correlated viral strain is indicated. For details on mutant selection, see methods section. Three of the mutations D63G, P80R, and A119S were specific to Delta (PANGO lineage B.1.617.2), Gamma (PANGO lineage P.1) and Zeta (PANGO lineage P.2) strains of SARS-CoV-2, respectively and were lineage-defining. Three of the mutations P67S (PANGO lineage BA.1.20), E136D (PANGO lineage BE.1.1) and P151S (PANGO lineage BA.4) belonged to circulating Omicron variants of concern[1].

Supplementary Fig. 2

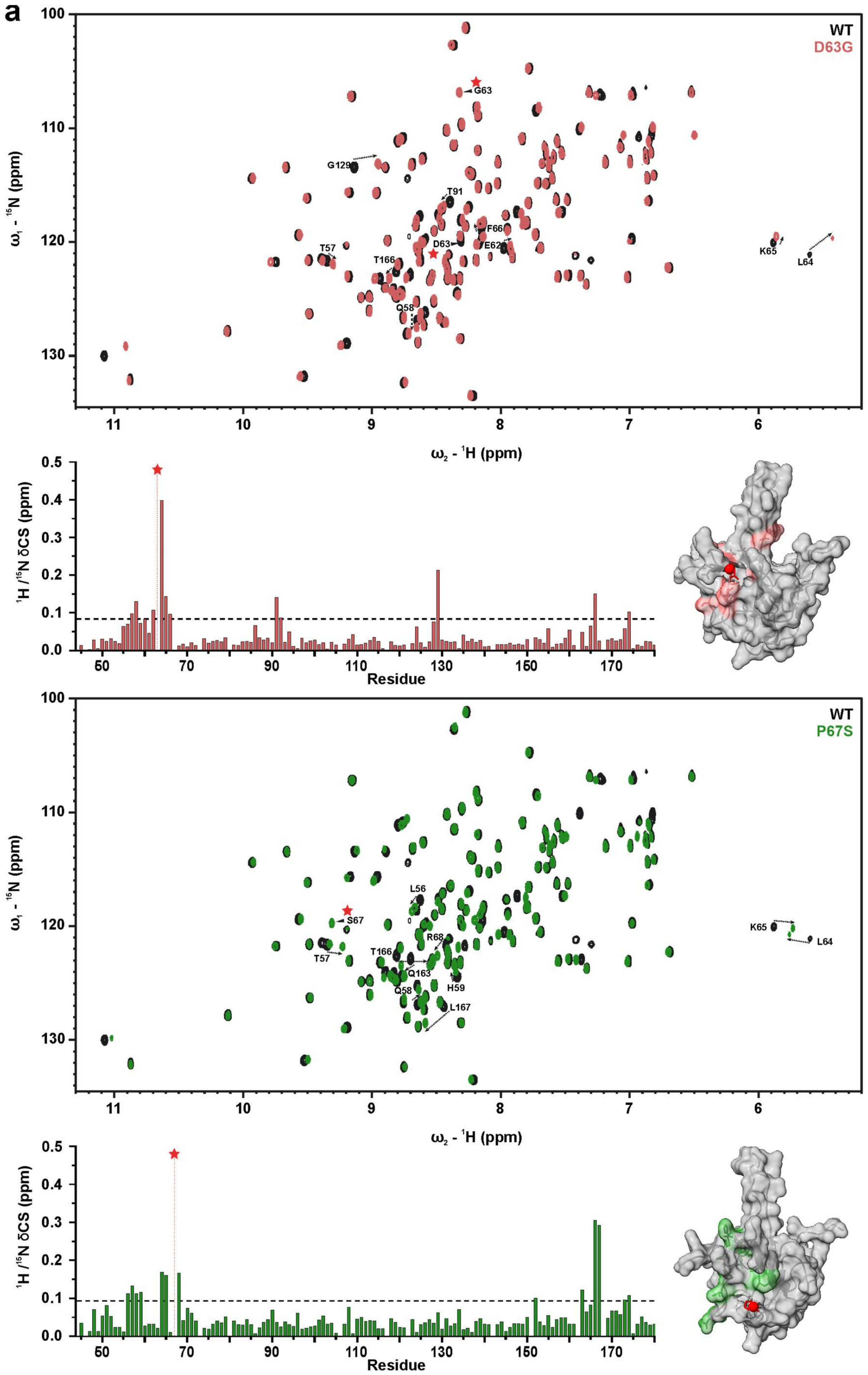

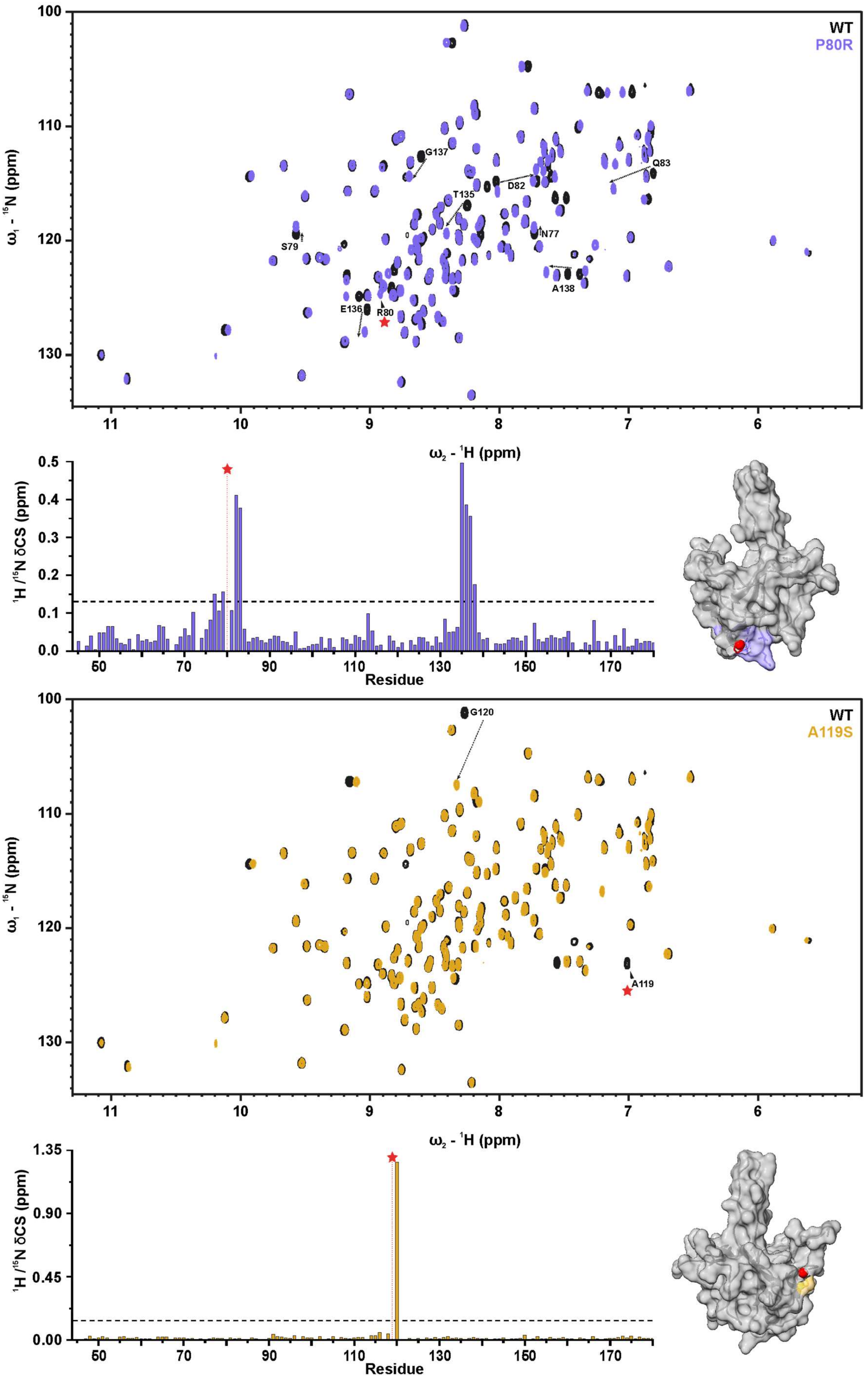
WT
P80R
ω1 - 15N (ppm)
ω2 - 1H (ppm)
G137
Q83
D82
T135
N77
S79
A138
E136
R80
1H /15N δCS (ppm)
Residue
WT
A119S
G120
A119

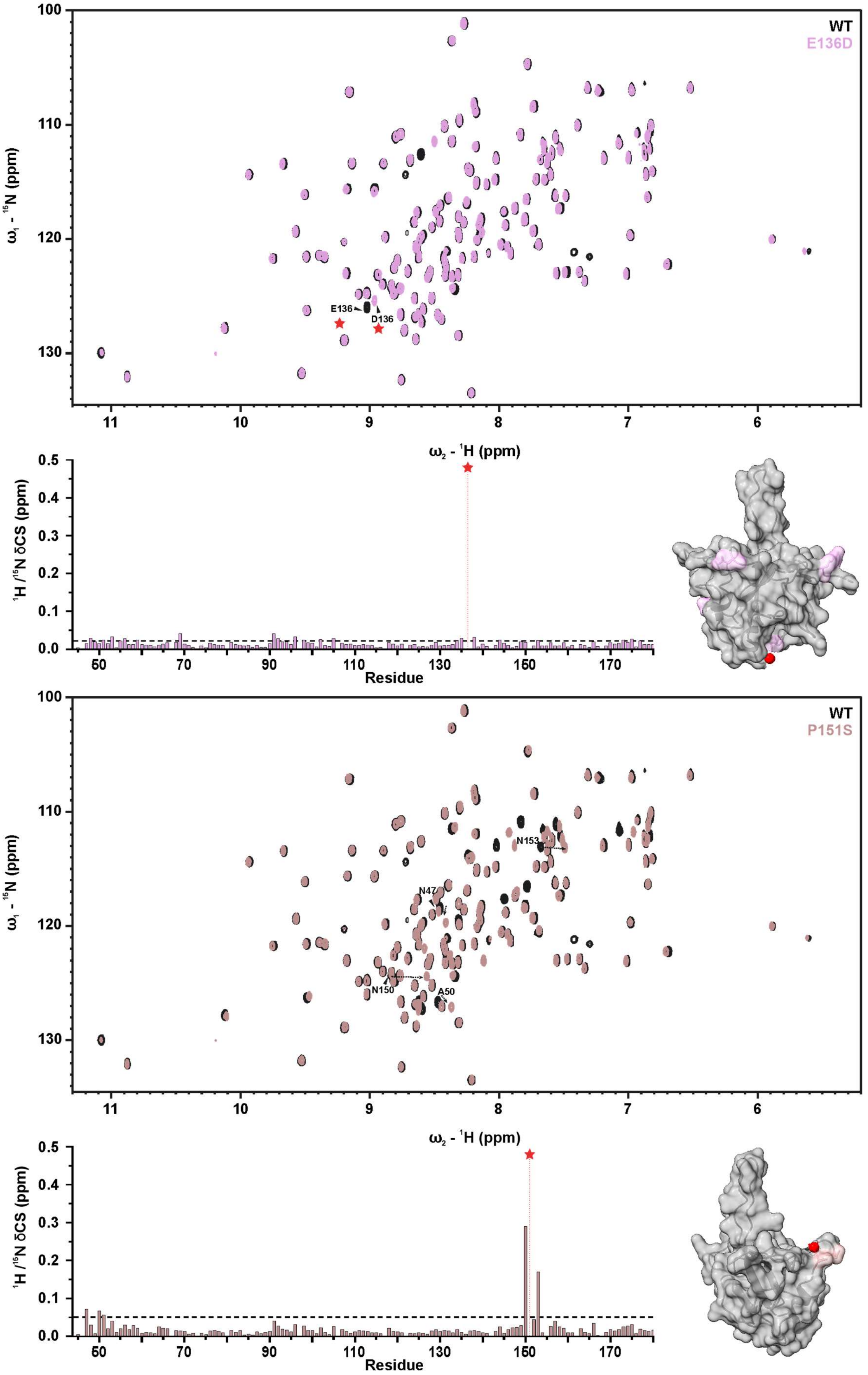
WT
E136D
E136
D136
ω1 - 15N (ppm)
ω2 - 1H (ppm)
1H /15N δCS (ppm)
Residue
WT
P151S
N153
N47
N150
A50
ω1 - 15N (ppm)
ω2 - 1H (ppm)
1H /15N δCS (ppm)
Residue

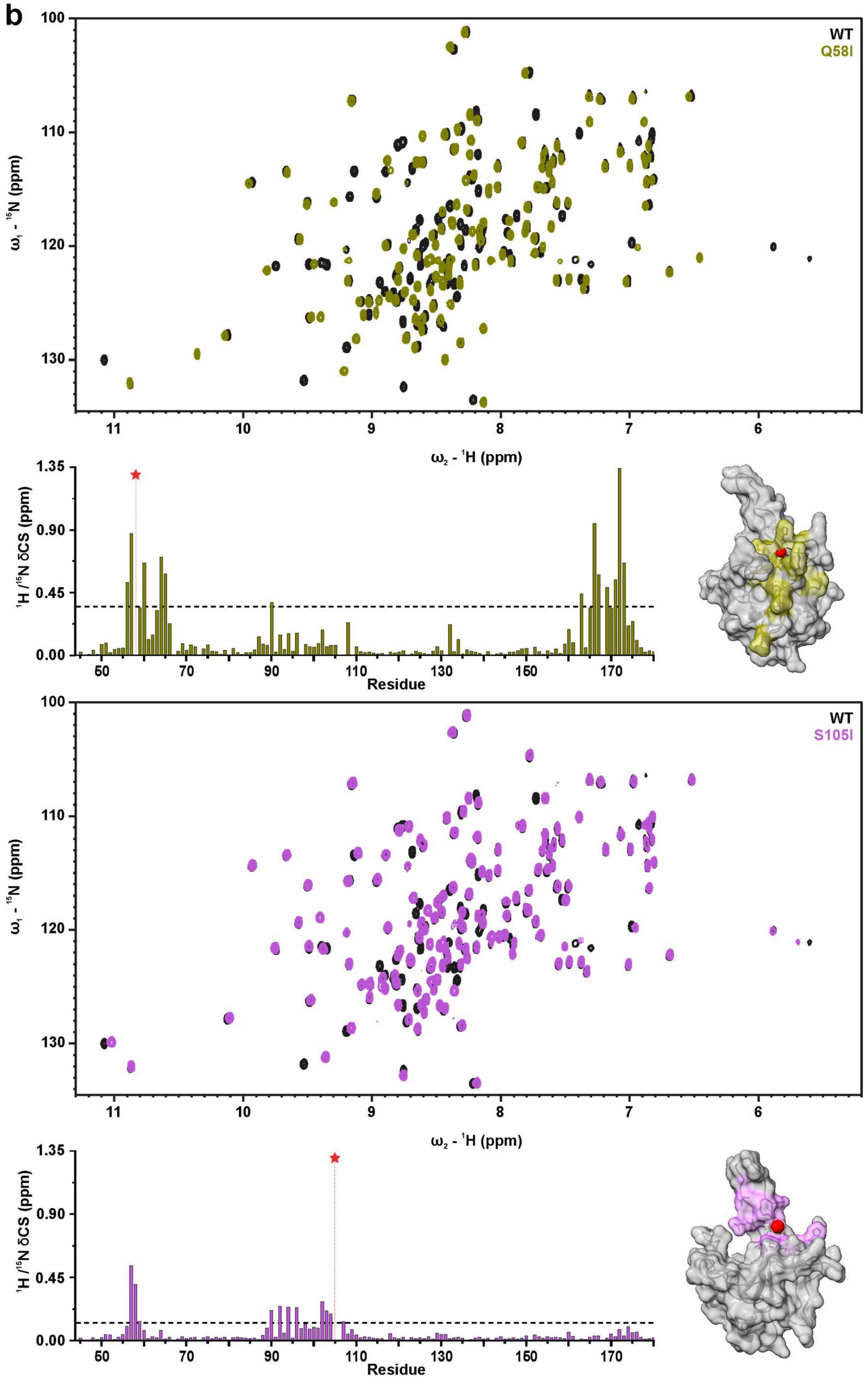
b
WT
Q58I
ω1 - 15N (ppm)
ω2 - 1H (ppm)
1H /15N δCS (ppm)
Residue
WT
S105I
ω1 - 15N (ppm)
ω2 - 1H (ppm)
1H /15N δCS (ppm)
Residue

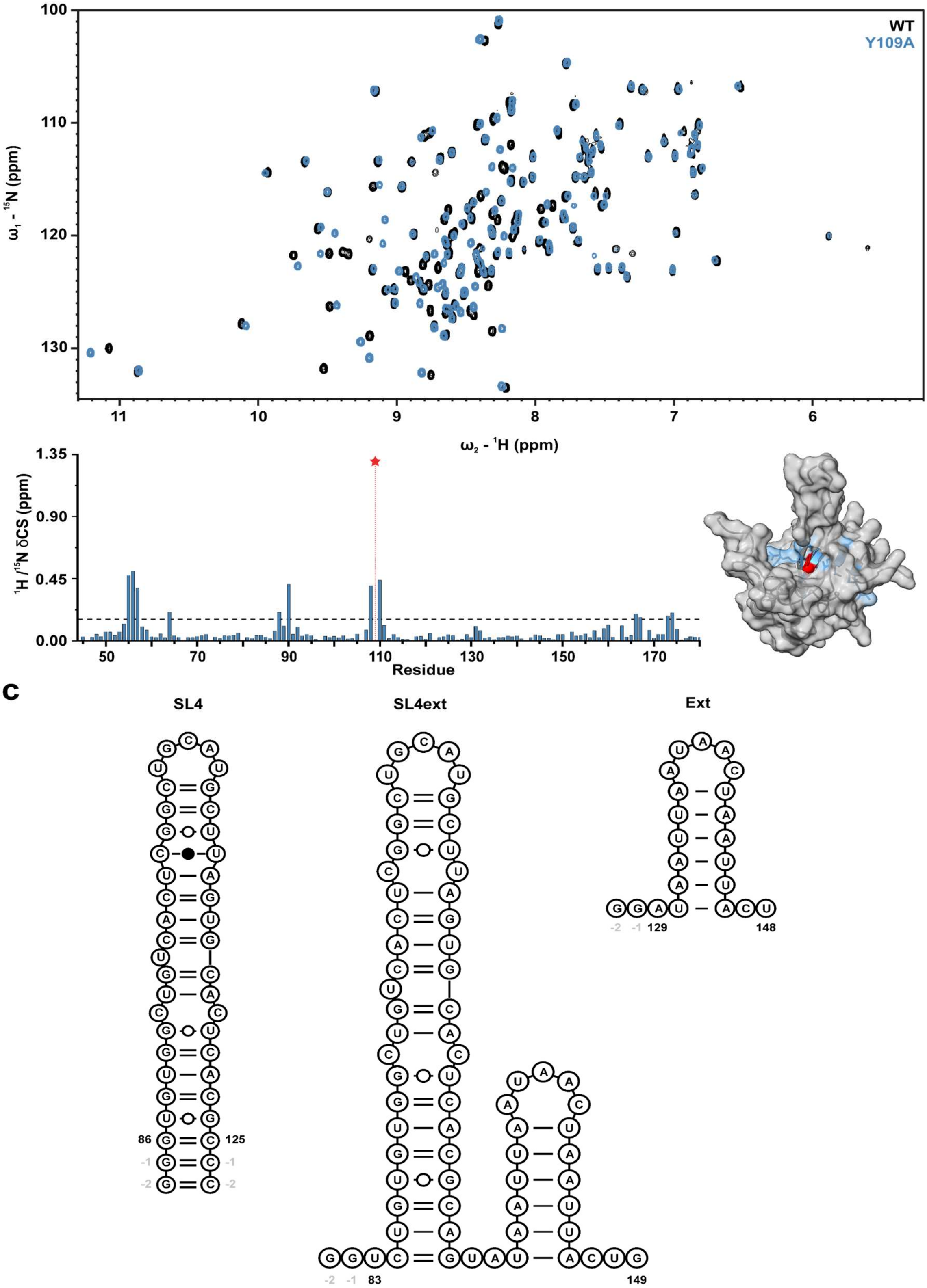
WT
Y109A
ω1 - 15N (ppm)
ω2 - 1H (ppm)
1H /15N δCS (ppm)
Residue
C
SL4
SL4ext
Ext

d

	50 · 75 · 100 · 125 · 150 · 175	
D63G	GGLPNNTASWFTALTQHGKEGLKFPRGQGVPINTNSSPDDQIGYYRRATRRIRGGDGKMKDLSPRWYFYYLGTGPEAGLPYGANKDGIIWVATEGALNTPKDHIGTRNPANNAAIVLQLPQGTTLPKGFYAEGSRGGS	98.5%
P67S	GGLPNNTASWFTALTQHGKEDLKFSRGQGVPINTNSSPDDQIGYYRRATRRIRGGDGKMKDLSPRWYFYYLGTGPEAGLPYGANKDGIIWVATEGALNTPKDHIGTRNPANNAAIVLQLPQGTTLPKGFYAEGSRGGS	98.5%
P80R	GGLPNNTASWFTALTQHGKEDLKFPRGQGVPINTNSSRDDQIGYYRRATRRIRGGDGKMKDLSPRWYFYYLGTGPEAGLPYGANKDGIIWVATEGALNTPKDHIGTRNPANNAAIVLQLPQGTTLPKGFYAEGSRGGS	98.5%
Q58I	GGLPNNTASWFTALTIHGKEDLKFPRGQGVPINTNSSPDDQIGYYRRATRRIRGGDGKMKDLSPRWYFYYLGTGPEAGLPYGANKDGIIWVATEGALNTPKDHIGTRNPANNAAIVLQLPQGTTLPKGFYAEGSRGGS	94.2%
S105I	GGLPNNTASWFTALTQHGKEDLKFPRGQGVPINTNSSPDDQIGYYRRATRRIRGGDGKMKDLIPRWYFYYLGTGPEAGLPYGANKDGIIWVATEGALNTPKDHIGTRNPANNAAIVLQLPQGTTLPKGFYAEGSRGGS	98.5%
Y109A	GGLPNNTASWFTALTQHGKEDLKFPRGQGVPINTNSSPDDQIGYYRRATRRIRGGDGKMKDLSPRWAFYYLGTGPEAGLPYGANKDGIIWVATEGALNTPKDHIGTRNPANNAAIVLQLPQGTTLPKGFYAEGSRGGS	97.8%

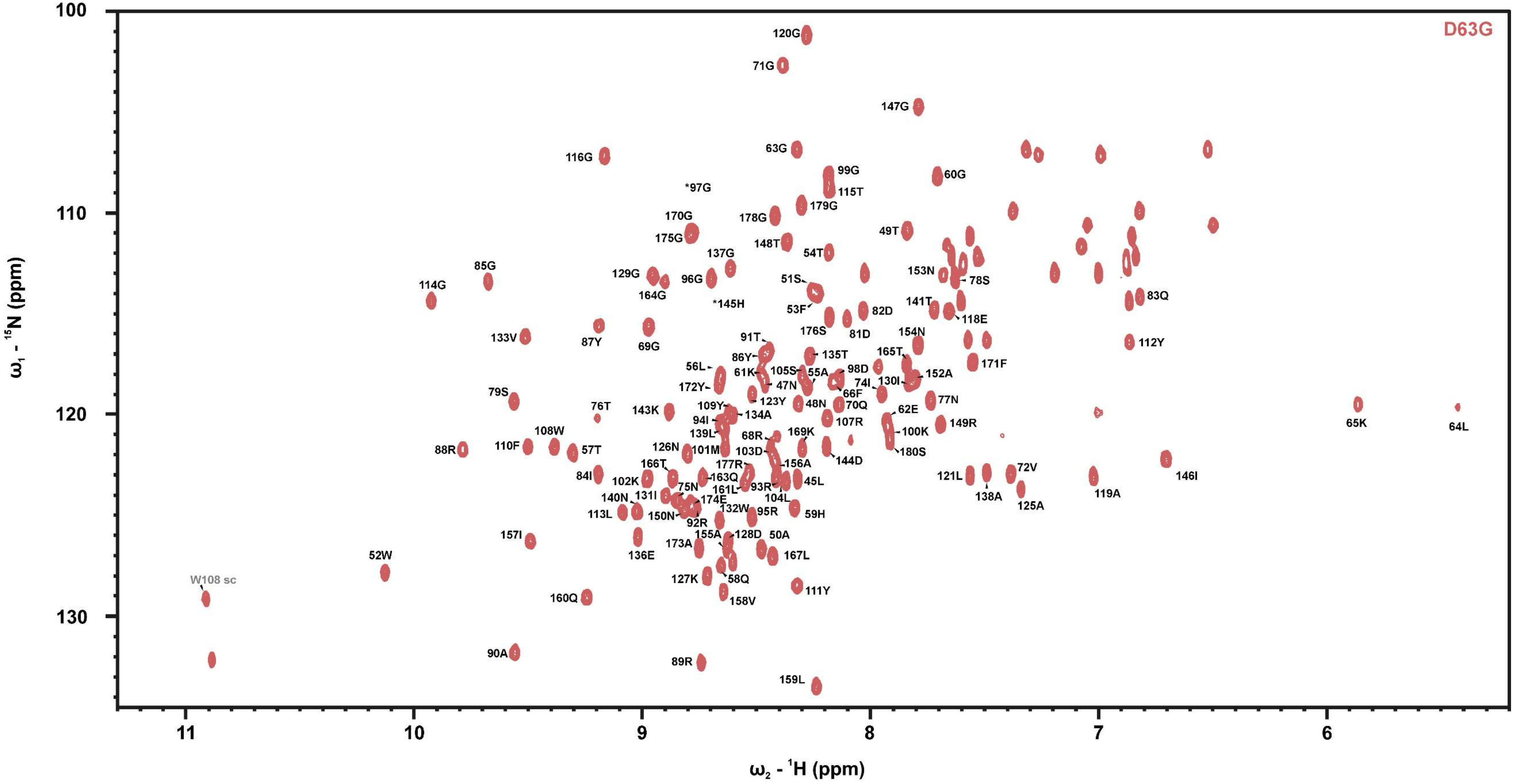

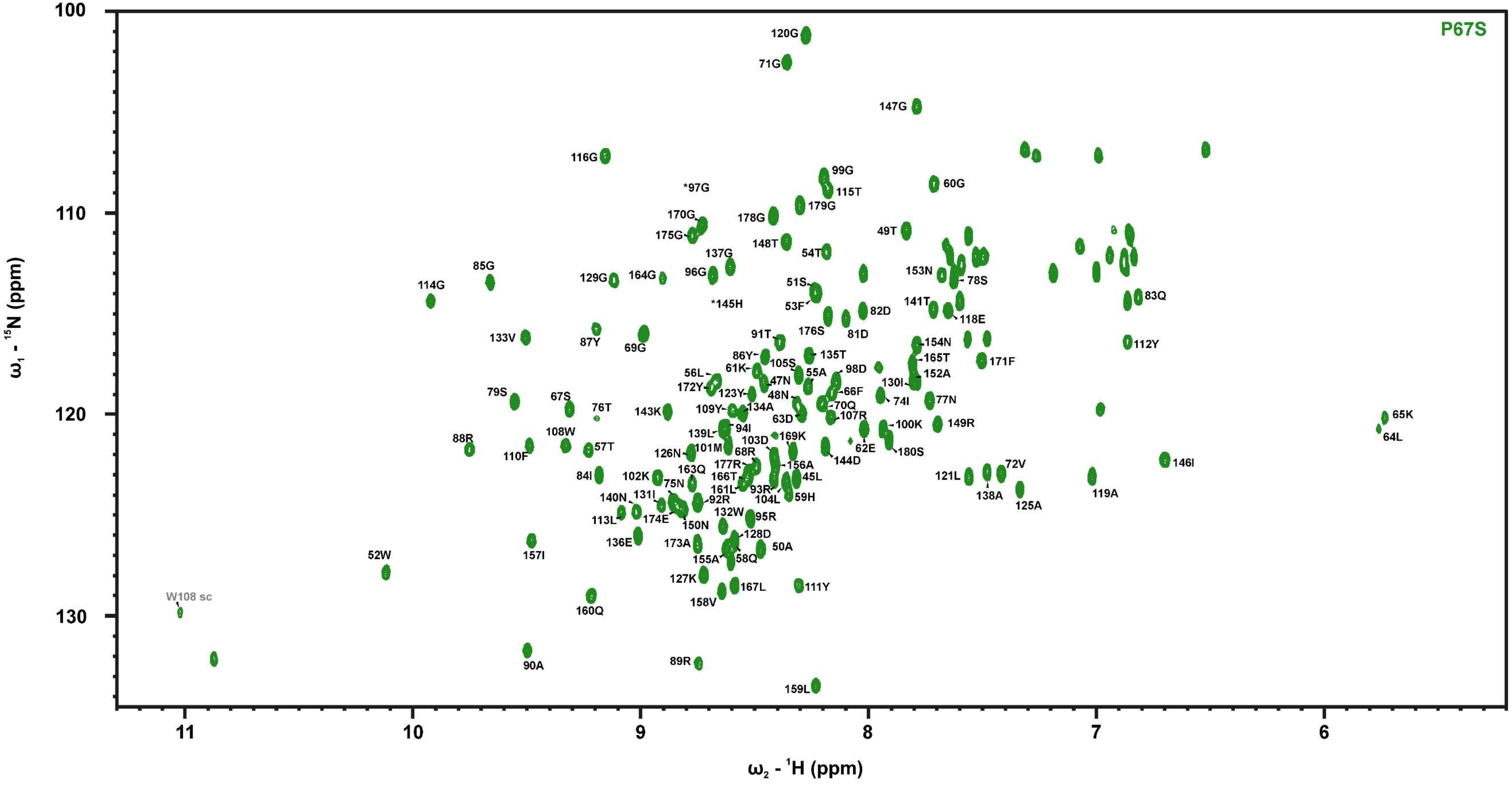
P67S
ω1 - 15N (ppm)
ω2 - 1H (ppm)
100
110
120
130
11
10
9
8
7
6
120G
71G
147G
116G
99G
60G
*97G
115T
179G
170G
178G
175G
49T
148T
54T
137G
85G
129G
164G
96G
153N
78S
114G
51S
83Q
141T
*145H
53F
82D
118E
91T
176S
81D
133V
87Y
69G
154N
112Y
86Y
135T
165T
171F
105S
61K
98D
56L
55A
152A
47N
130I
172Y
66F
48N
123Y
74I
77N
79S
67S
70Q
76T
109Y
134A
143K
107R
65K
63D
149R
100K
94I
64L
88R
108W
139L
169K
103D
62E
180S
110F
101M
57T
68R
126N
144D
146I
177R
156A
72V
163Q
84I
102K
166T
45L
121L
75N
161L
93R
119A
138A
125A
140N
131I
92R
104L
59H
174E
132W
113L
95R
150N
128D
136E
173A
50A
155A
58Q
52W
157I
127K
167L
111Y
158V
W108 sc
160Q
90A
89R
159L

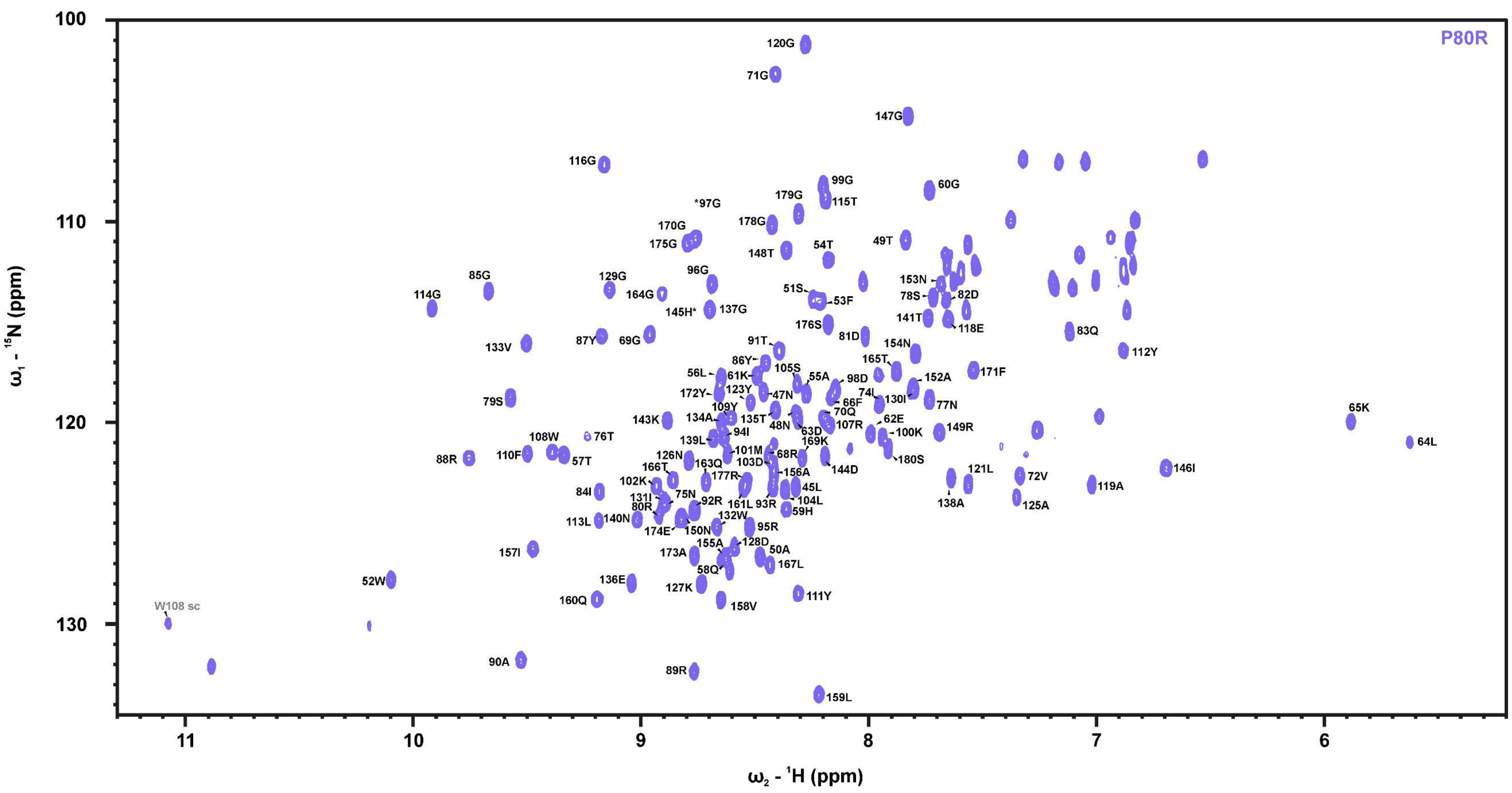
P80R
ω₁ - ¹⁵N (ppm)
ω₂ - ¹H (ppm)
100
110
120
130
11
10
9
8
7
6
120G
71G
147G
116G
99G
60G
179G
115T
*97G
178G
170G
175G
148T
54T
49T
96G
85G
129G
51S
53F
153N
114G
164G
78S
82D
145H*
137G
141T
176S
118E
81D
83Q
91T
154N
133V
87Y
69G
112Y
86Y
165T
105S
56L
61K
55A
98D
152A
171F
123Y
74L
172Y
47N
130I
66F
77N
79S
109Y
70Q
134A
62E
143K
135T
48N
107R
149R
65K
94I
63D
100K
139L
169K
64L
108W
76T
101M
68R
180S
88R
110F
57T
126N
103D
144D
166T
163Q
156A
121L
146I
102K
177R
72V
84I
45L
119A
131I
75N
92R
161L
93R
104L
138A
80R
59H
125A
113L
140N
132W
174E
150N
95R
155A
128D
157I
173A
50A
167L
58Q
52W
136E
127K
111Y
160Q
158V
W108 sc
90A
89R
159L

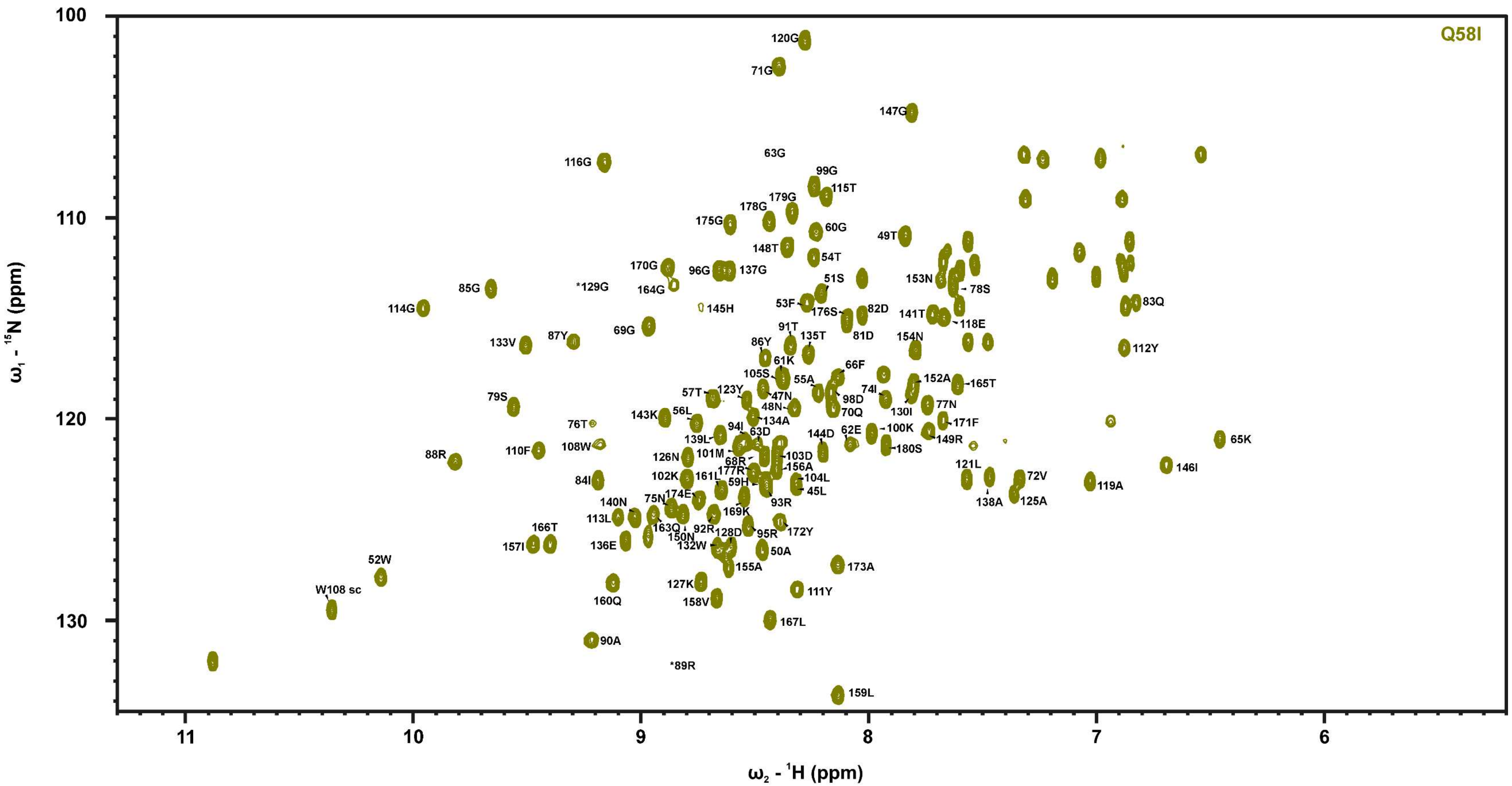

Q58I
100
110
120
130
11
10
9
8
7
6
ω1 - 15N (ppm)
ω2 - 1H (ppm)
120G
71G
147G
63G
116G
99G
115T
179G
178G
175G
60G
49T
148T
54T
170G
96G
137G
*129G
164G
51S
153N
78S
85G
53F
176S
82D
141T
83Q
114G
145H
118E
69G
91T
135T
81D
154N
133V
87Y
86Y
112Y
61K
66F
105S
55A
152A
165T
57T
123Y
47N
74I
79S
98D
77N
48N
130I
70Q
56L
143K
134A
171F
76T*
94I
63D
144D
62E
100K
139L
149R
65K
108W
110F
180S
88R
101M
103D
126N
68R
156A
121L
146I
177R
104L
72V
102K
161L
59H
84I
45L
119A
174E
138A
125A
140N
75N
93R
169K
113L
163Q
92R
172Y
166T
150N
128D
95R
157I
136E
132W
50A
155A
173A
52W
127K
W108 sc
111Y
160Q
158V
167L
90A
*89R
159L

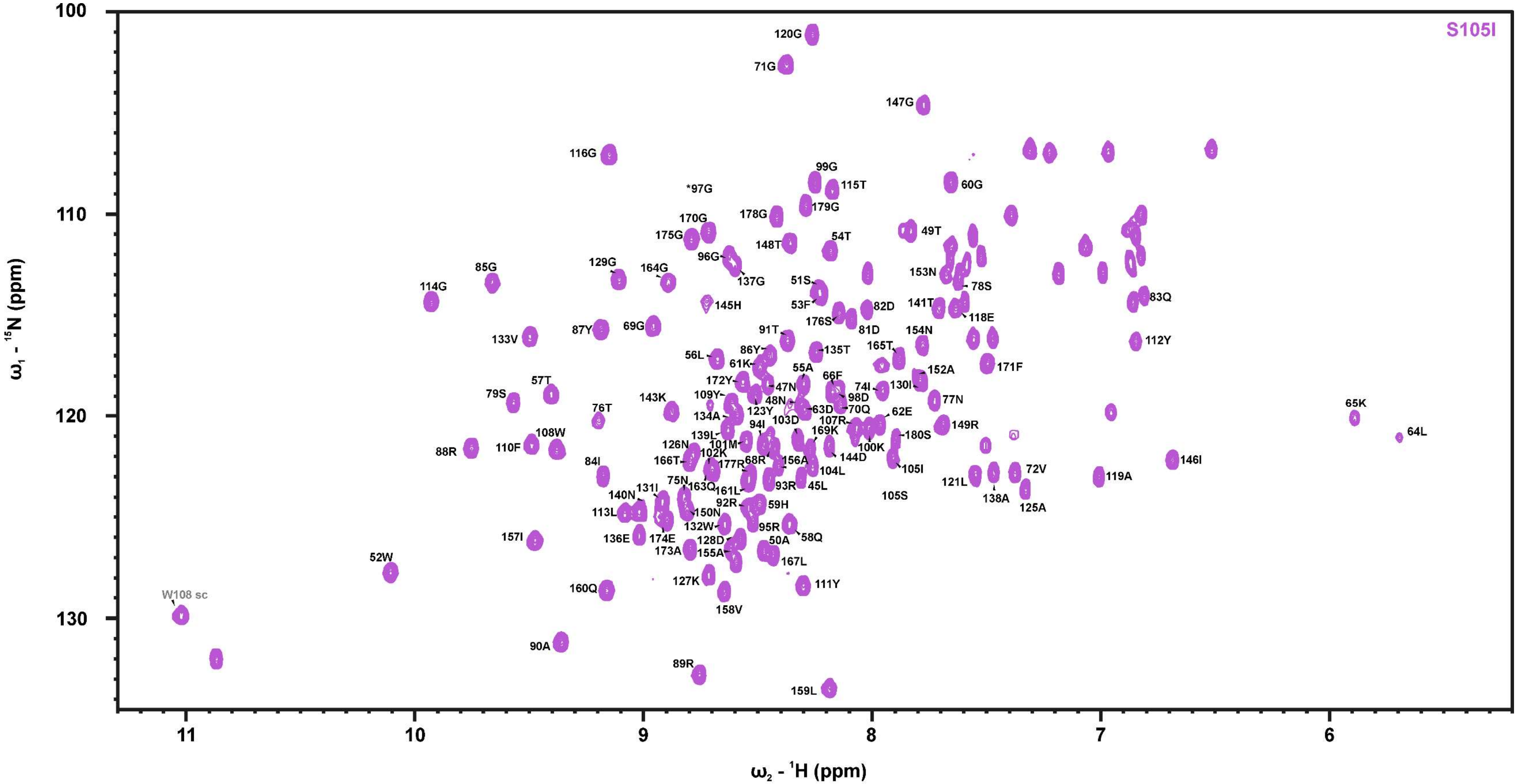

S105I
ω1 - 15N (ppm)
ω2 - 1H (ppm)
100
110
120
130
11
10
9
8
7
6
120G
71G
147G
116G
99G
115T
60G
*97G
178G
179G
170G
175G
148T
54T
49T
96G
137G
129G
164G
85G
114G
51S
153N
78S
83Q
53F
82D
141T
145H
176S
118E
69G
87Y
133V
91T
81D
154N
112Y
56L
86Y
135T
165T
61K
55A
171F
152A
172Y
66F
74I
130I
57T
47N
79S
143K
109Y
48N
98D
77N
76T
123Y
63D
70Q
134A
103D
107R
62E
94I
169K
149R
108W
139L
180S
88R
110F
101M
100K
126N
102K
144D
166T
68R
156A
146I
84I
177R
104L
105I
72V
75N
163Q
121L
119A
131I
161L
93R
45L
138A
140N
105S
92R
59H
125A
113L
150N
132W
95R
157I
136E
174E
128D
50A
58Q
173A
155A
167L
52W
127K
111Y
160Q
158V
W108 sc
90A
89R
159L
65K
64L

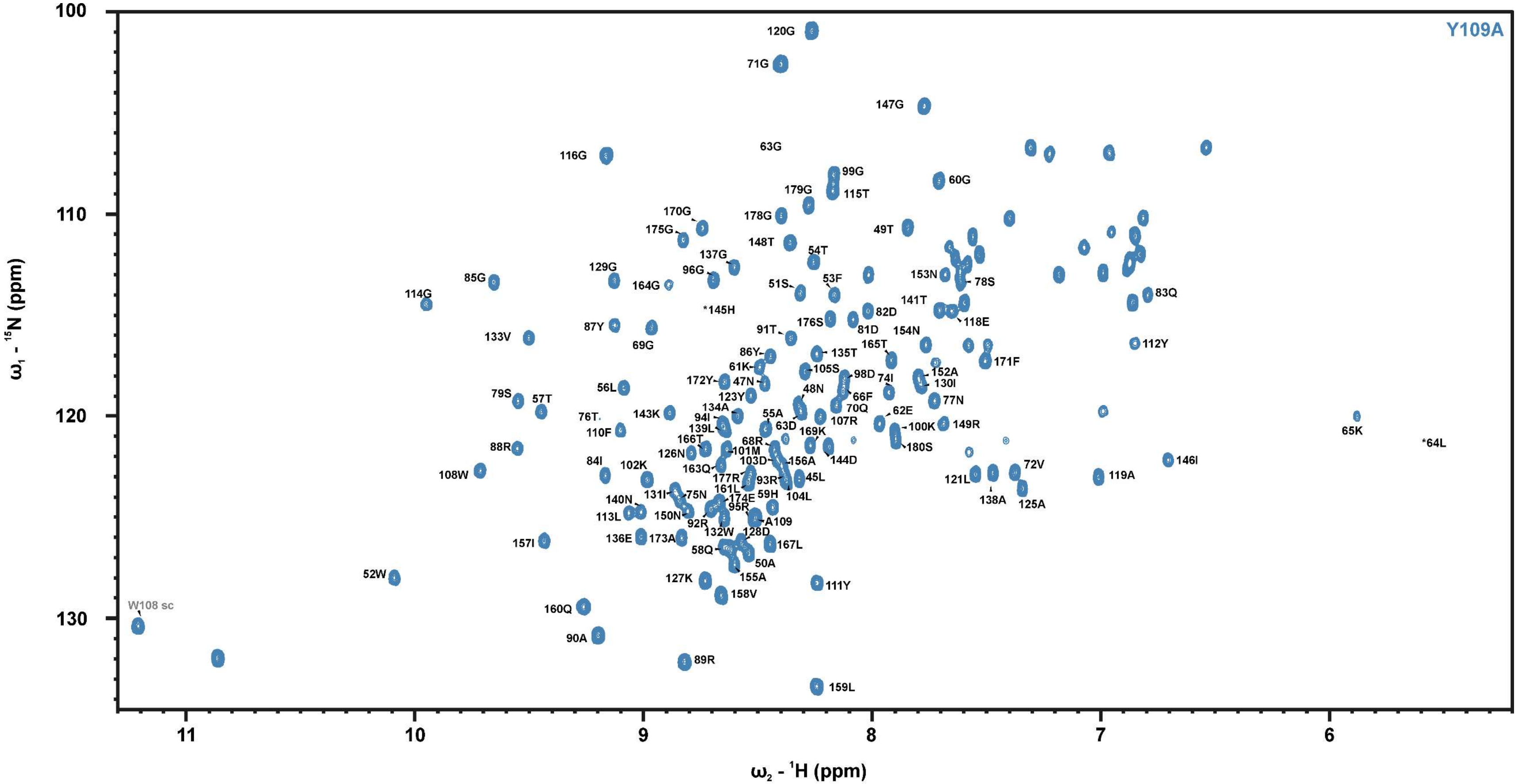

Supplementary Fig. 2: NMR observed chemical shift differences (CSD) induced by mutations in the NTD. a) $^1H/^{15}N$ HSQC overlays of NTD nat_mutants and **b)** des_mutants as indicated in the spectra with NTD_WT and plots of derived CSDs. Residues for nat_mutants that show significant CSDs are labeled in the spectra. Of note: CSDs of sidechain residues are not considered in the CSD plot, showing only changes for backbone NH groups. Prolines and peaks that were line-broadened beyond detection appear as gaps in the plot. Significant CSDs (Average + 1 SD, dotted line in CSD plots) are mapped onto the NTD_WT NMR structure (PDB 6YI3). C_α at the respective site of mutation is shown as red sphere. Spectra were recorded in NTD buffer for 140 µM protein at a 600 MHz spectrometer at 298 K. **c)** Secondary structures of viral RNA elements used in this study[2,3] obtained from RNApdbee[4] and plotted using RNArtist (https://github.com/fjossinet/RNArtist). **d)** Sequence coverage of assignment (Gray – not assigned and color – site of mutation) and protein backbone chemical shift assignments that have been deposited at the BMRB. Assigned tryptophan sidechain peak (W108 sc - $^1H\varepsilon$-$^{15}N\varepsilon$) of Q58I is labelled in black, whereas W108 sc assignment transferred from WT for D63G, P67S, P80R, S105I and Y109A is labelled in gray. Peaks of residues (97G and 145H) with low S/N that are not visible in the shown spectra are labelled at their respective location with an asterisk. Unassigned peaks are glutamine sidechain ($^1H\varepsilon$-$^{15}N\varepsilon$), asparagine sidechain ($^1H\delta$-$^{15}N\delta$) and aliased (by 1 spectral width in ^{15}N dimension) arginine sidechain ($^1H\varepsilon$-$^{15}N\varepsilon$) peaks.

Supplementary Fig. 3

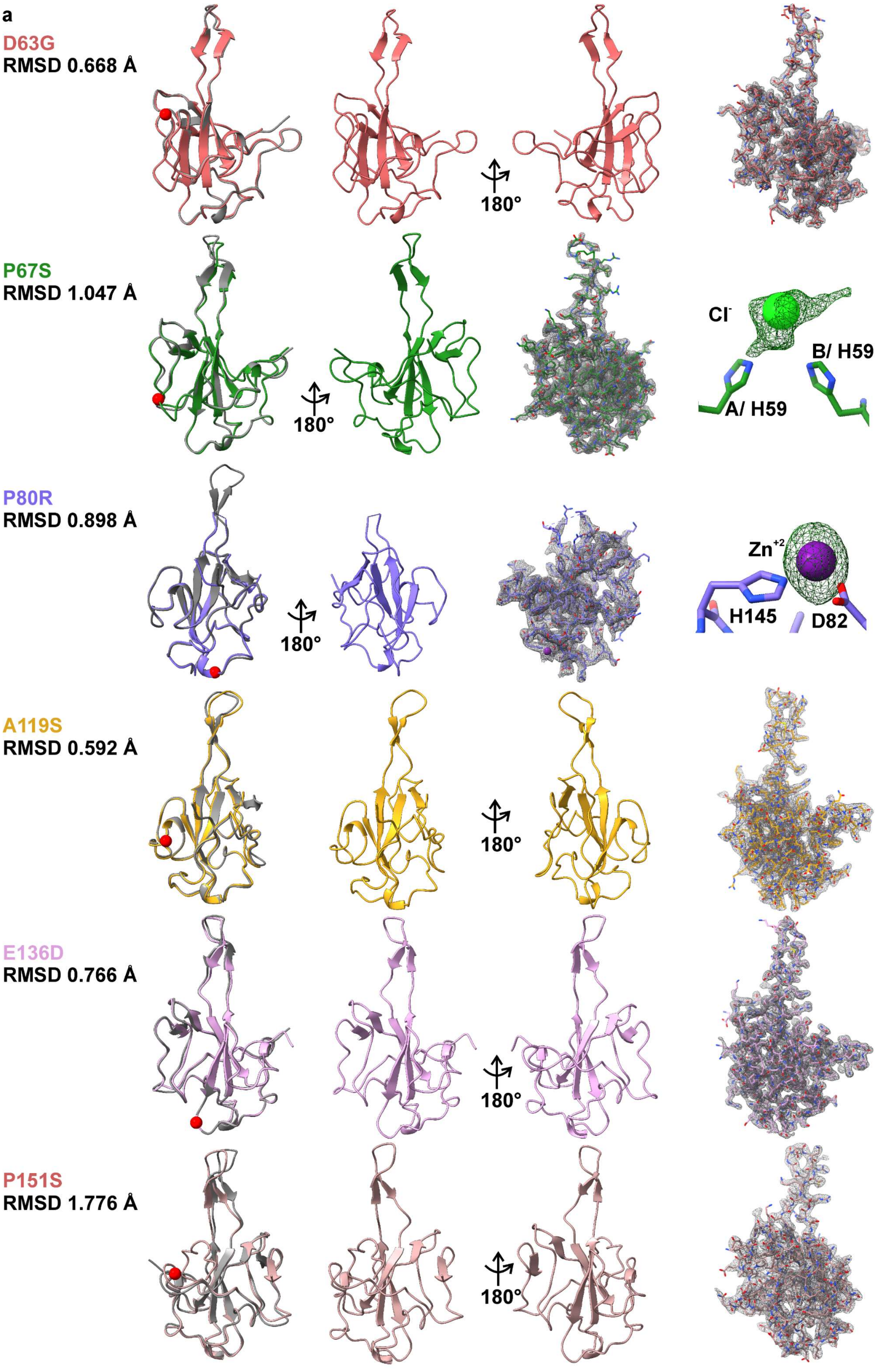

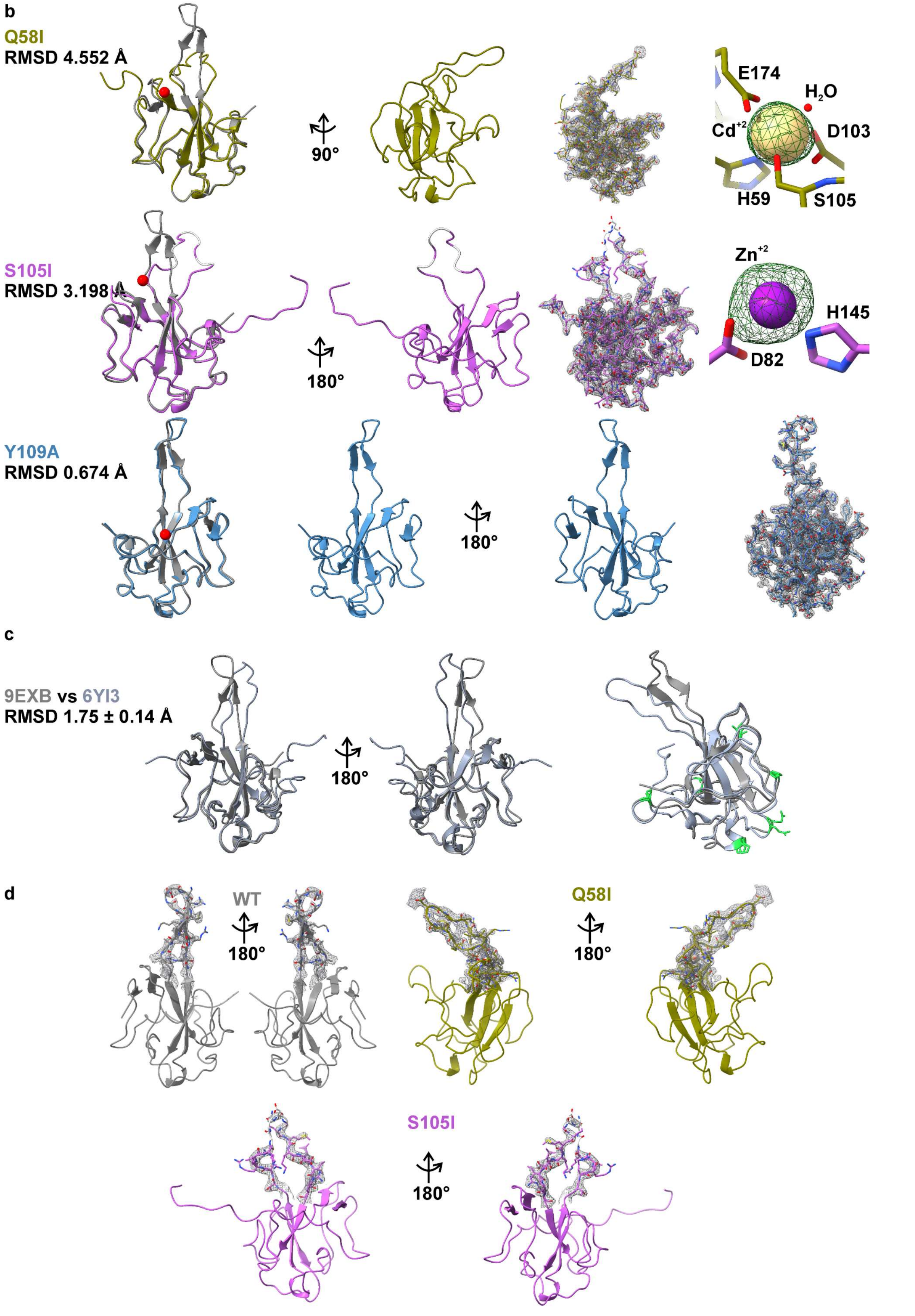

Supplementary Fig. 3: High resolution crystal structures of NTD mutants. a) Overlay of crystal structures of nat_mutants (in color) and **b)** des_mutants and their respective root mean square deviations to WT structure (in

grey). The Cα at the site of mutation is shown as red sphere on WT structure. The structures of the mutants are shown from two perspectives on the right. Missing β-hairpin residues in the structures of NTD_P80R and NTD_S105I are depicted as broken lines. 2mFo-DFc map (in gray) shows the electron density at 1σ for the protein chain and mFo-DFc map (in green) at 3σ for ligand. Residues and water coordinating the bound metal ion are labelled. **c)** Overlay of the WT crystal structure from this study (Chain C) to the NMR structure (model 35). The average RMSD between the 4 chains in the WT crystal structure and the 40 conformers in the NMR structure (excluding highly flexible residues 89 to 107) is given with standard deviation. Highlighted in green stick representation are the residues of nat_mutations in both NMR and crystal structure. **d)** Crystal structures of WT, Q58I and S105I with electron density (2mFo-DFc) shown in gray at 1σ for residues 89 to 107 from two perspectives. Continuous density allowed complete model building of loop residues 89 to 107 (represented as sticks) in WT and Q58I. For S105I, residues 94 to 102 were modelled for completeness but lack uniform and continuous experimental electron density. In the region between 94 and 102, residues are shown in white (no electron density) and color (electron density below 1σ).

Supplementary Fig. 4

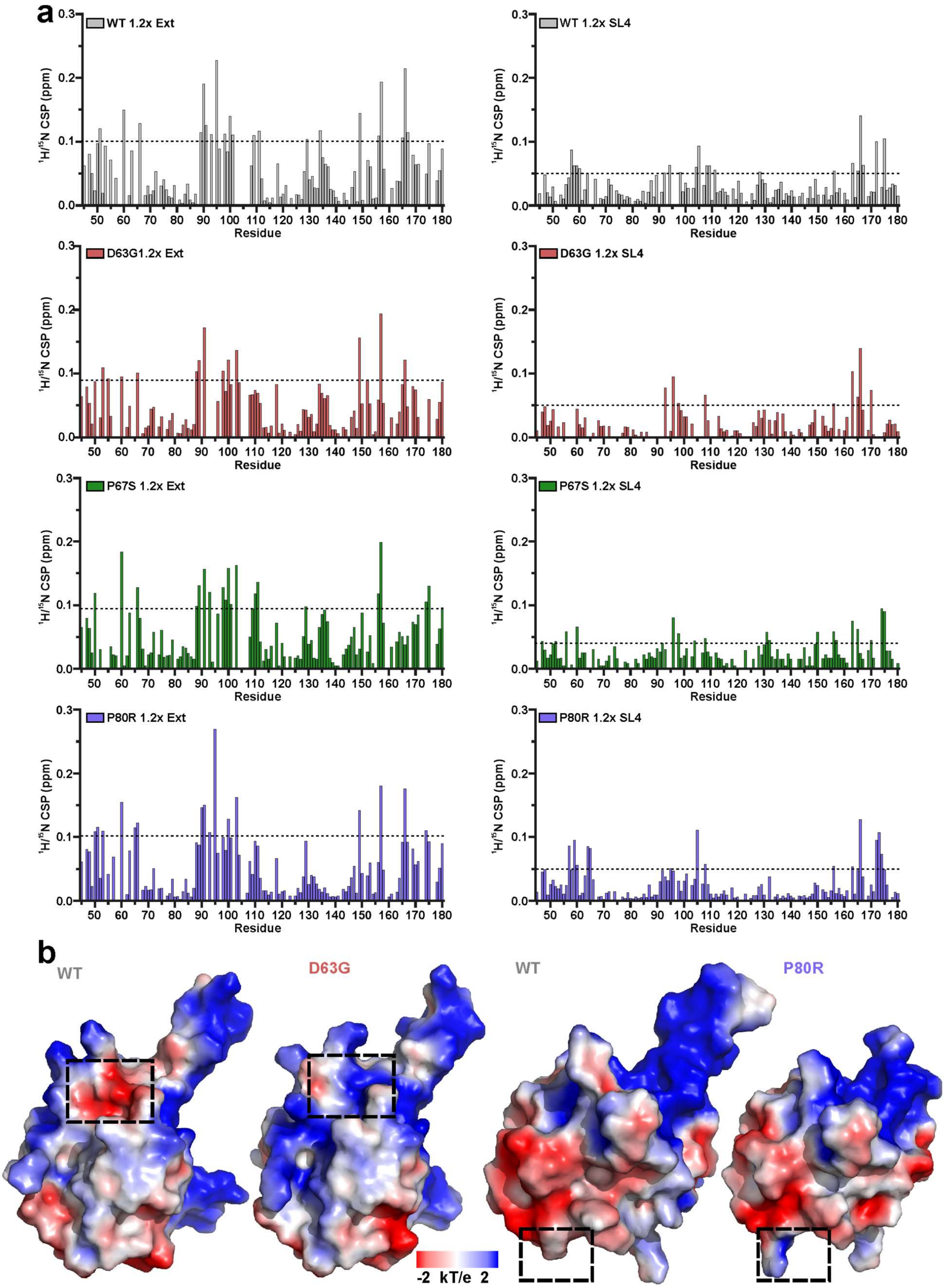

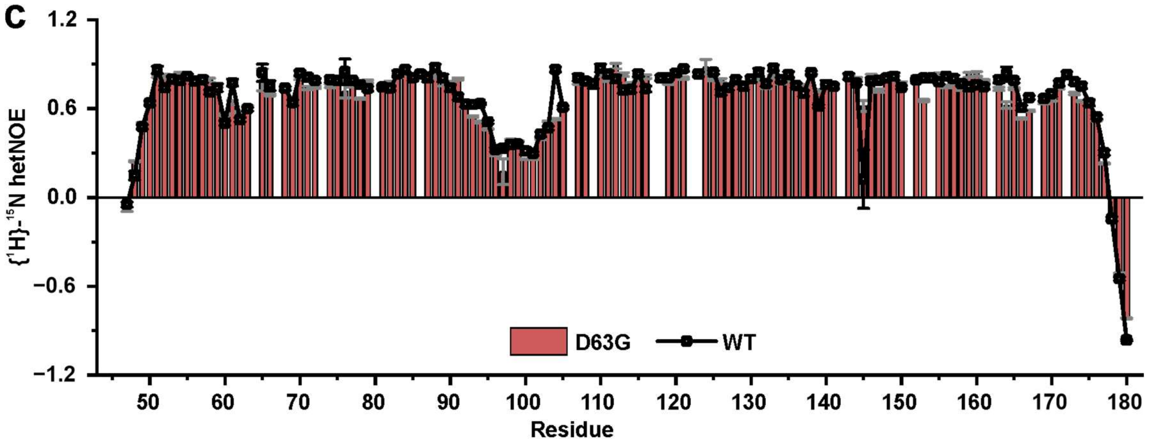

Supplementary Fig. 4: a) $^{1}H/^{15}N$ CSP plots for D63G, P80R and WT comparing apo proteins and their complexes with 1.2 equivalents of SL4 and Ext RNA, respectively. The threshold for significance of CSPs (average + 1 SD) is indicated by a dotted line. **b)** Electrostatic surface potential of WT compared to that of D63G and P80R obtained from Pymol APBS plugin[5]. Area around the site of mutation is highlighted by a dashed box. **c)** $\{^{1}H\}$-^{15}N hetNOE comparison of D63G (as bars in color with error bars in gray) and WT (as squares with error bars in black). Error bars represent the intensity ratio of saturated over unsaturated peaks, respectively, considering the local noise of each peak according to equation 4 in the Methods section. Spectra were recorded with 750 µM of protein at a 600 MHz spectrometer, at 298 K. Prolines and peaks that were line-broadened beyond detection appear as gaps in the plot.

Supplementary Fig. 5

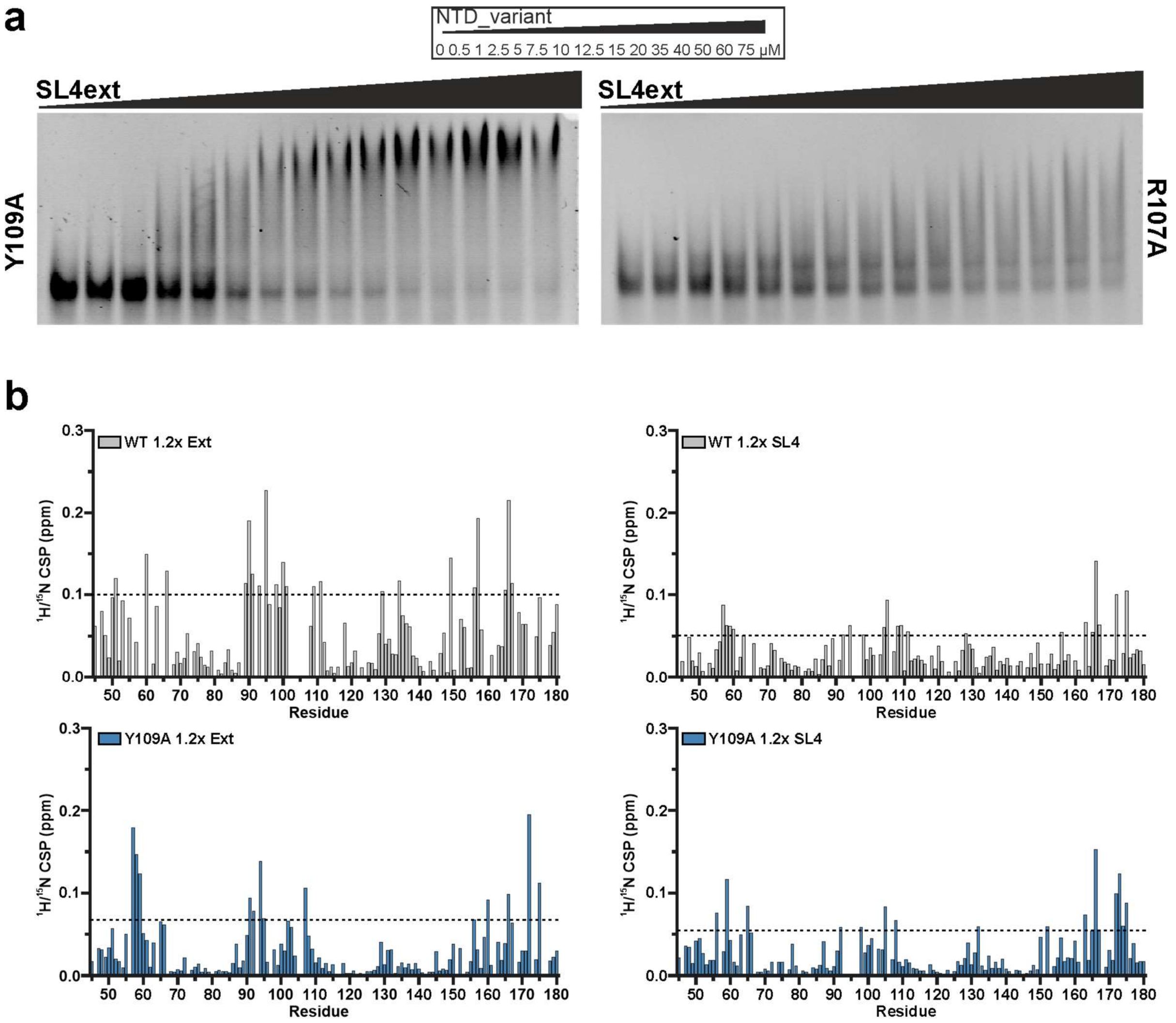

Supplementary Fig. 5: a) Qualitative EMSAs with des_mutants Y109A and R107A. SL4ext RNA (3 µM) has been incubated with increasing concentrations of protein as indicated above. **b)** $^{1}H/^{15}N$ CSP plots for Y109A and WT, respectively, in complex with either 1.2 equivalents of Ext or SL4 at a 600 MHz spectrometer at 298 K. Prolines and peaks that were line-broadened beyond detection appear as gaps in the plot. The threshold for significance of CSPs (average + 1 SD) is indicated by a dotted line.

Supplementary Fig. 6

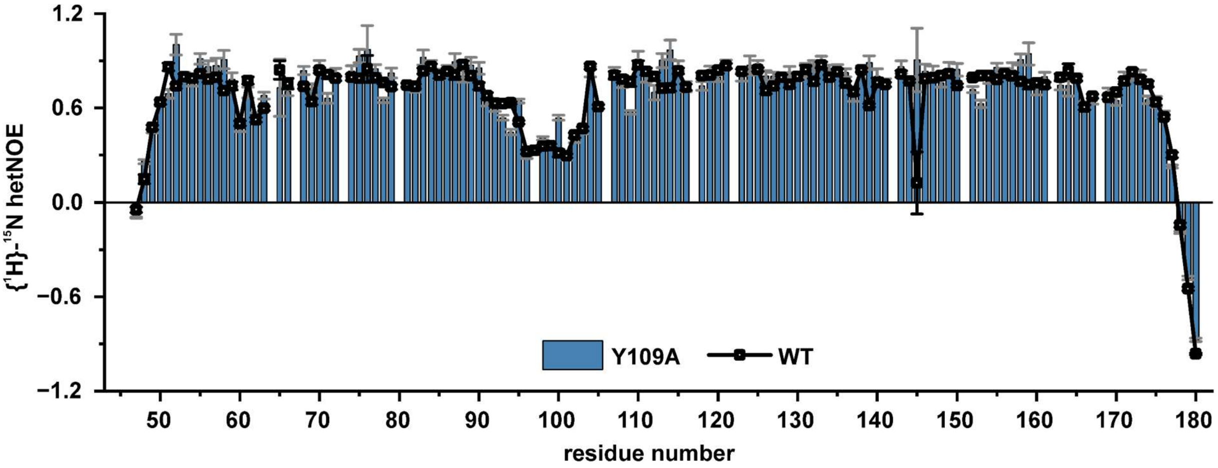

Supplementary Fig. 6: {^{1}H}-^{15}N hetNOE comparison of Y109A (as bars in color with error bars in gray) and WT (as squares with error bars in black). Error bars represent the intensity ratio of saturated over unsaturated peaks, respectively, considering the local noise of each peak according to equation 4 in the Methods section. Spectra were recorded for 750 μM protein at a 600 MHz spectrometer, at 298 K. Prolines and peaks that were line-broadened beyond detection appear as gaps in the plot.

Supplementary Fig. 7

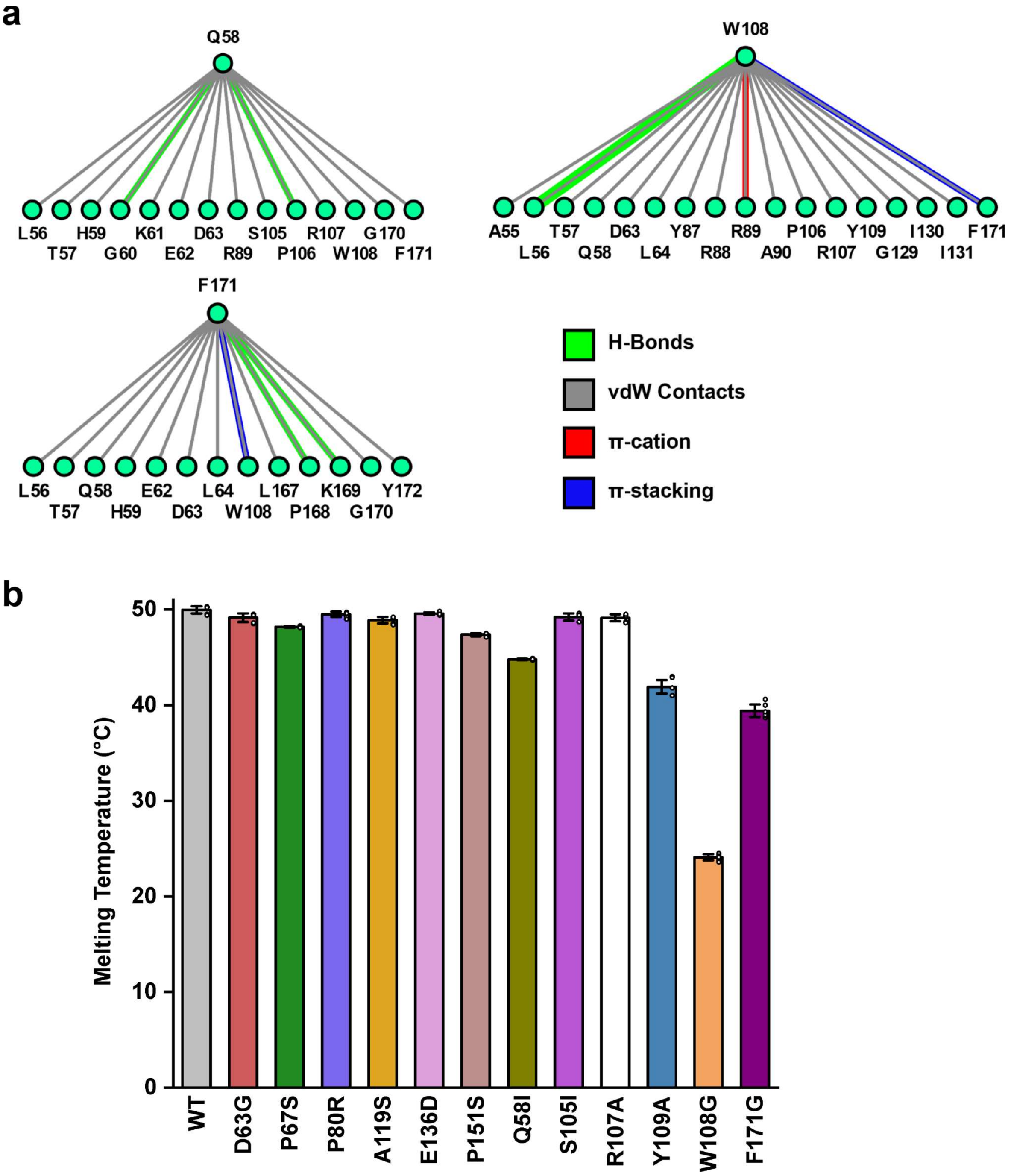

Supplementary Fig. 7: a) Detailed interaction map of core network residues as observed in our WT crystal structure, visualized and depicted in iCn3D[6]. **b)** Tm values (in °C) obtained from nano-DSF measurements for WT, nat_mutants and des_mutants used in this study. Errors represent standard deviation (SD) from three biological replicates (N=3) each measured in duplicates with values representing the average. Individual replicates are shown as white circles to the right of the bar plot.

Supplementary Fig. 8

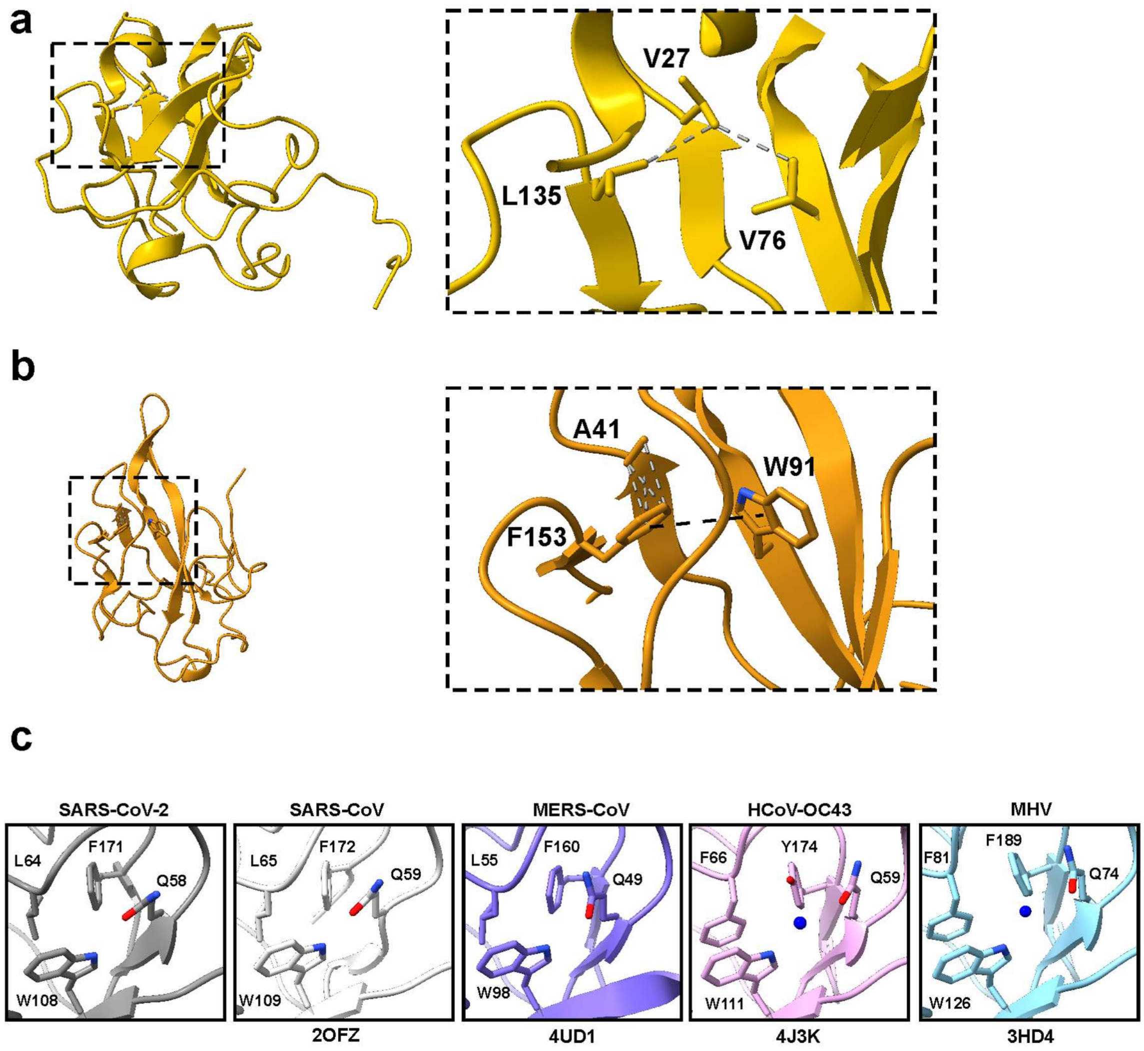

Supplementary Fig. 8: Respective NTD core network in *Alpha*-, *Beta*- and *Gammacoronaviruses*. a) Core interaction network in Human coronavirus NL63 (HCoV-NL63) 5N4K[7]. **b)** Core interaction network in infectious bronchitis virus (IBV) 2BXX[8]. Zoom-ins show residues involved in the network of interactions and the different types of contacts between them. Grey dashed lines indicate van der Waals contacts, the black dashed line shows an additional π-π interactions. **c)** Zoom-ins depicting the relative orientation of core network residues (Q-W-F/(Y)) in NTD representatives from four different *Betacoronavirus* species compared to SARS-CoV-2. Water molecules that are involved in mediating contacts are shown as blue spheres.

Supplementary Fig. 9

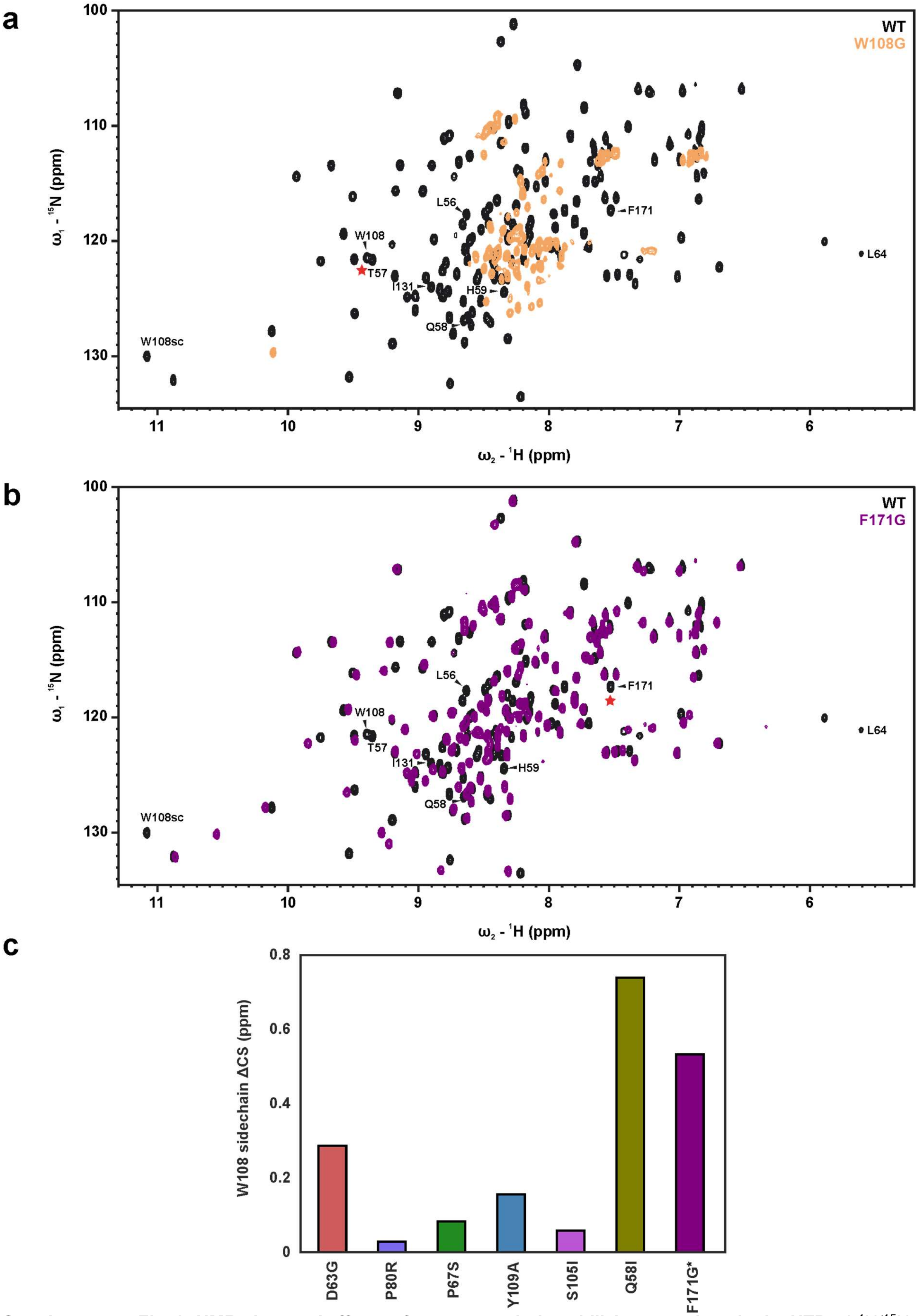

Supplementary Fig. 9: NMR observed effects of core network-destabilizing mutations in the NTD. a) $^{1}H/^{15}N$-HSQC overlay of WT and W108G showing that the mutation of tryptophan 108 to glycine results in a highly unstable protein prone to precipitation at 298 K even at low concentrations. **b)** $^{1}H/^{15}N$-HSQC overlay of WT and F171G showing significant and broadly dispersed CSDs. In both overlays, site of mutation is shown as red star and residues

located in different regions of the contact network are labelled with their assignments referring to the WT spectra. Spectra were recorded for 50 μM (W108G) and 750 μM (F171G) protein at 298 K at a 600 MHz spectrometer. **c)** Effect of nat_ and des_mutations on the $^{1}H/^{15}N$ CSD of the tryptophan 108 sidechain compared to WT. Asterisk denotes W108 sidechain assignment in F171G that was transferred from WT based on $^{1}H,^{15}N$ chemical shift similarity.

Supplementary Fig. 10

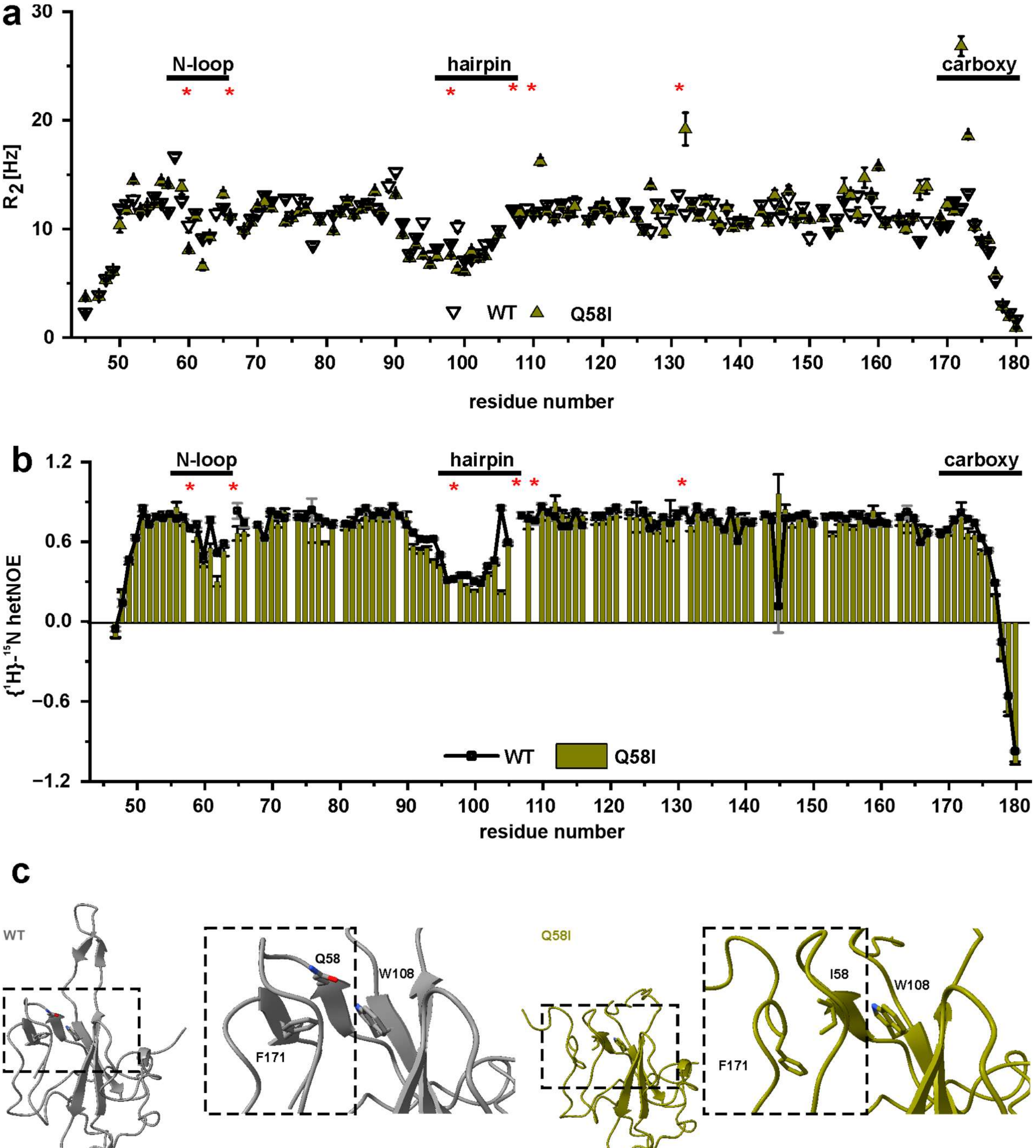

Supplementary Fig. 10: a) Residue-specific R_2 relaxation rates for Q58I (upward facing triangles in color) vs WT (downward facing triangles). Error bars are shown in gray. The Levenberg-Marquardt model was used to fit the relaxation rates. Error bars represent standard errors from the covariance matrix automatically calculated by the program CCPNMR Analysis. Red asterisks denote residues with invisible backbone NH resonances due to line broadening in Q58I. Spectra were acquired from 750 µM protein at 298 K. **b)** $\{^1H\}$-^{15}N hetNOE values for Q58I (as bars in color with error bars in black) and WT (as squares with error bars in black). Error bars represent the intensity ratio of saturated over unsaturated peaks, respectively, considering the local noise of each peak according to equation 4 in the Methods section. Red asterisks denote residues with invisible backbone NH resonances due to line broadening in Q58I. Spectra were acquired at 298 K. **c)** Crystal structures of WT (left) and des_mutant Q58I (right). Zoom-ins show the destabilized carboxy finger in the mutant compared to WT. Core network residues are labelled and represented as sticks.

Supplementary Fig. 11

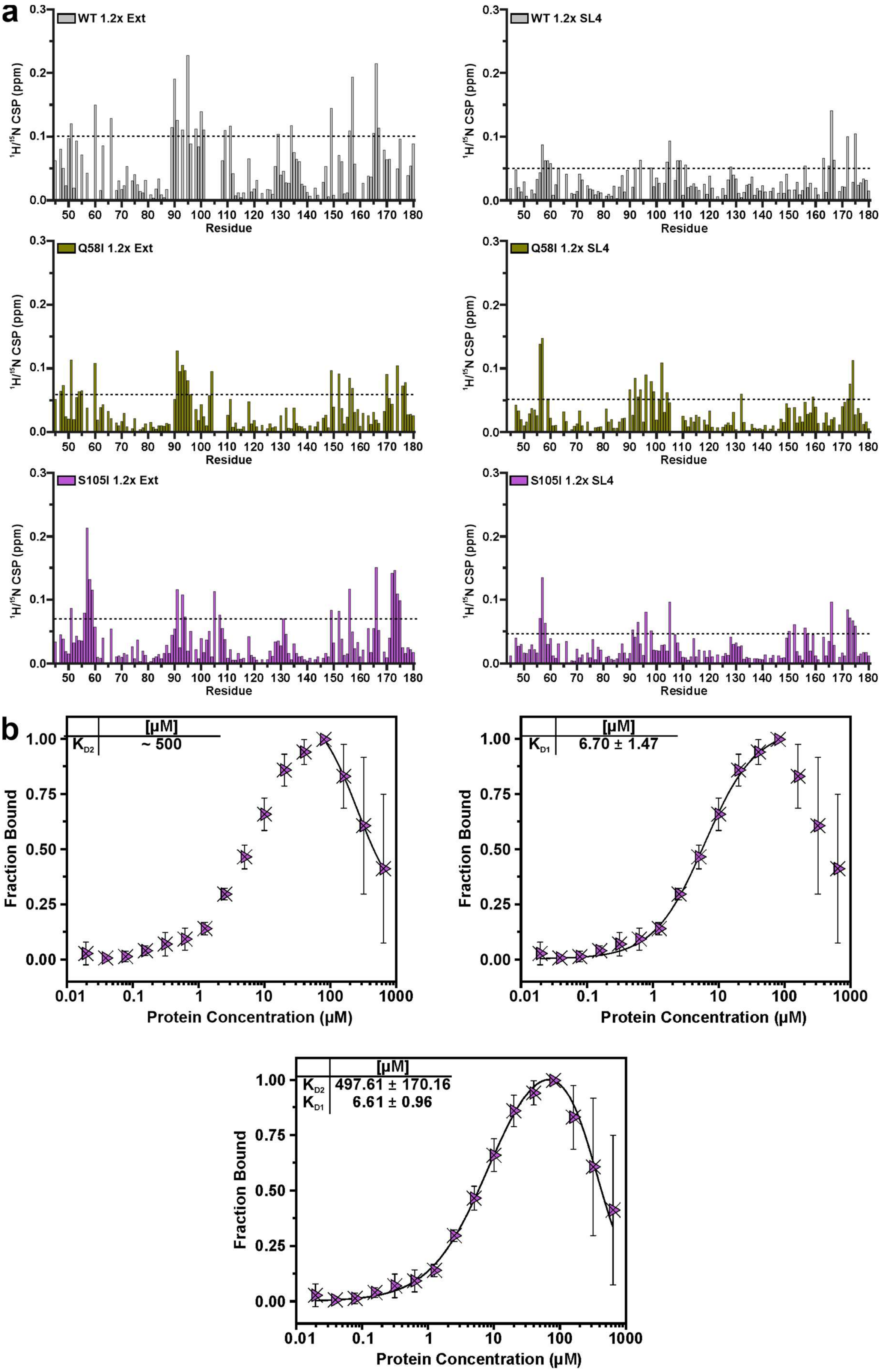

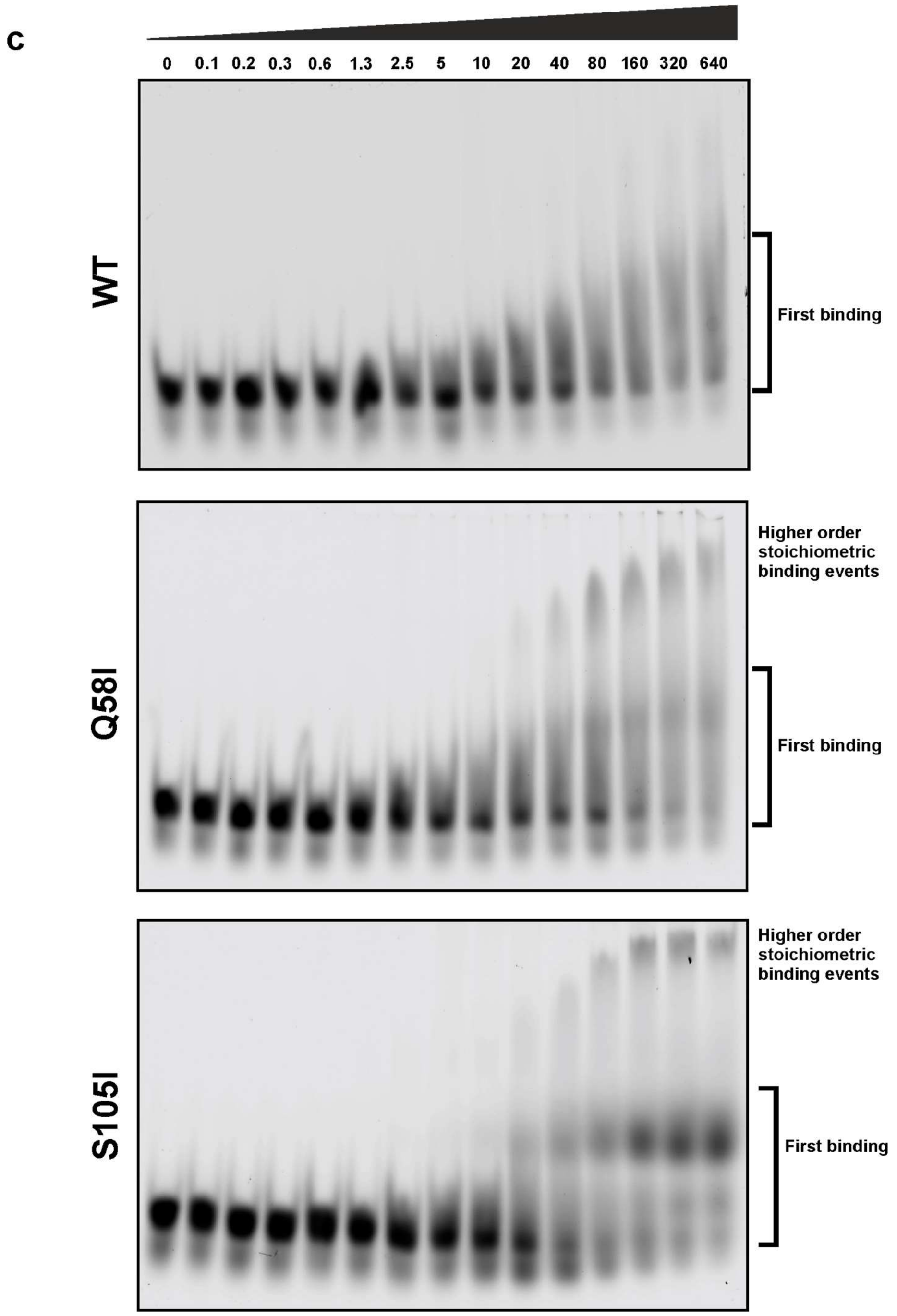

Supplementary Fig. 11: a) $^{1}H/^{15}N$ CSP plots for WT, Q58I and S105I comparing apo proteins to complexes with 1.2 equivalents of either Ext (left) or SL4 (right). Underlying spectra were recorded in NTD buffer for 70 µM protein (apo) and 70 µM protein with 84 µM RNA at a 600 MHz spectrometer at 298 K. Prolines and peaks that were line-broadened beyond detection appear as gaps in the plot. The threshold for significance of CSPs (average + 1 SD) is indicated by a dotted line. **b)** MST-derived K_D values of viral SL4 RNA when titrated with des_mutant S105I. Data are presented as mean values +/- SD from three biological replicates (N=3), each measured in duplicate. Error bars represent SD from replicates. The binding curve implies a binding stoichiometry above 1:1 indicated by the last three titration points with non-monotonic behavior. Individual single fits (Hill model) to the first and second binding events are shown. Note that quantification of the second binding event can only be estimated by the software and appears at high, non-physiological concentrations of protein. For comparison, a biphasic fit including both binding events (BiHill model) is shown below and reveals the independence of binding sites from the fit procedure and from each other. This suggests the later binding event is non-specific and subordinate. Errors derive from three biological replicates. **c)** Fluorescent EMSAs of 3'-Cy5 labelled non-target RNA SL4 when titrated with NTD WT and des_mutants. Protein concentrations are given above. The occurrence of higher order stoichiometric binding events for mutant S105I (as well as the related mutant Q58I) are suggested to reflect the second binding event observed in MST (panel b).

Supplementary Table 1: Triple resonance experiment details used for assignments.

Mutant	BMRB ID	% NH assigned	Residues with unassigned NHs	Sample concentration (µM)	Experiment type	Spectrometer type (^{1}H Larmor frequency)	Acquisition details			
							F3 points	F2 points	F1 points	Scans
D63G	52471	98.5	G43, G44	750	HSQC	600 MHz		2048	128	40
				750	HNCACB	600 MHz	2048	72	122	16
				750	HN(CA)CO	600 MHz	1536	64	48	16
				750	HNCO	600 MHz	2048	48	48	16
P67S	52472	98.5	G43, G44	750	HSQC	600 MHz		2048	128	40
				750	HNCACB	600 MHz	2048	72	122	16
				750	HNCO	600 MHz	2048	32	48	16
P80R	52473	98.5	G43, G44	750	HSQC	600 MHz		2048	128	40
				750	HNCACB	600 MHz	2048	72	122	16
				750	HNCO	600 MHz	2048	32	48	16
S105I	52474	98.5	G43, G44	750	HSQC	700 MHz		2048	128	40
				610	HNCACB	700 MHz	1536	112	140	16
				750	HNCO	600 MHz	2048	32	48	16
Y109A	52470	97.8	G43, G44, G97	750	HSQC	600 MHz		2048	128	40
				750	HNCACB	600 MHz	2048	72	122	16
				750	HN(CA)CO	600 MHz	1536	64	56	16
Q58I	52469	94.2	G43, G44, I58, L64, G97, R107, Y109, I131	750	HSQC	600 MHz		2048	128	40
				750	HNCACB	600 MHz	2048	72	122	16
				750	HNCA	600 MHz	2048	72	64	28
				1500	HN(CA)CO	600 MHz	1536	64	52	16
				1500	HNCO	600 MHz	2048	32	48	16
				750	CBCA(CO)NH	600 MHz	2048	48	72	60
				750	^{15}N-NOESY	1.2 GHz	2048	80	96	16

*^{1}Hε-^{15}Nε (glutamine residues), ^{1}Hδ-^{15}Nδ (asparagine residues) and ^{1}Hε-^{15}Nε (tryptophan residues)

Supplementary Table 2: Protein crystallization conditions.

	NTD_WT	NTD_D63G	NTD_P67S	NTD_P80R	NTD_A119S
Concentration (mg/mL)	15	15	15	12	15
Temperature (K)	293	293	293	285	293
Protein buffer	20 mM Tris-HCl<br>50 mM NaCl<br>1 mM DTT<br>pH 8.0	20 mM Tris-HCl<br>50 mM NaCl<br>1 mM DTT<br>pH 8.0	20 mM Tris-HCl<br>50 mM NaCl<br>1 mM DTT<br>pH 8.0	20mM Tris-HCl<br>25mM Na2SO4<br>1mM DTT<br>pH 9.0	20 mM Tris-HCl<br>50 mM NaCl<br>1 mM DTT<br>pH 8.0
Crystallization conditions	100 mM MES pH 6.0<br>20 % PEG 6000<br>10 mM Zinc Chloride	50mM HEPES pH 6.8<br>150mM Sodium Chloride<br>24% PEG 3350<br>2% PEG 400	150mM Sodium chloride<br>28% PEG Smear Medium	22 % PEG 3350<br>200 mM Zinc Acetate dihydrate	100 mM Tris-HCl pH 8.5<br>200 mM Sodium acetate trihydrate<br>30% PEG 4000
Crystallization technique	Sitting drop vapor diffusion	Sitting drop vapor diffusion	Sitting drop vapor diffusion	Hanging drop vapor diffusion	Sitting drop vapor diffusion
Crystal shape	Single Rods	Single Rods	Single Rods	Hexagonal prisms	Single Rods
Days to grow crystals	3	3	3	2	3

	NTD_E136D	NTD_P151S	NTD_Q58I	NTD_S105I	NTD_Y109A
Concentration (mg/mL)	15	15	12	12	9
Temperature (K)	293	293	277	293	277
Protein buffer	20 mM Tris-HCl<br>50 mM NaCl<br>1 mM DTT<br>pH 8.0	20 mM Tris-HCl<br>50 mM NaCl<br>1 mM DTT<br>pH 8.0	20 mM Tris-HCl<br>50 mM NaCl<br>1 mM DTT<br>pH 8.0	20 mM Tris-HCl<br>50 mM NaCl<br>1 mM DTT<br>pH 8.0	20 mM Tris-HCl<br>50 mM NaCl<br>1 mM DTT<br>pH 8.0
Crystallization conditions	100 mM BICINE pH 9.3<br>20 % PEG 3350	100mM Sodium HEPES pH 7.5<br>4.3M Sodium chloride	100mM HEPES pH 7.5<br>200mM Ammonium sulfate<br>10mM Cadmium chloride<br>25% PEG Smear Medium<br>30mM Manganese chloride	22% PEG 3350<br>200mM Zinc Acetate dihydrate	30% PEG 5000
Crystallization technique	Sitting drop vapor diffusion	Sitting drop vapor diffusion	Hanging drop vapor diffusion	Hanging drop vapor diffusion	Sitting drop vapor diffusion
Crystal shape	Single Rods	Single Rods	Plates	Tetragonal bipyramids	Rod cluster
Days to grow crystals	3	7	3	4	1

Supplementary Table 3: Crystal data collection and refinement statistics.

**Data Collection**					
	**NTD_WT**	**NTD_D63G**	**NTD_P67S**	**NTD_P80R**	**NTD_A119S**
PDB	9EXB	9F83	9EZB	9F7A	9F5L
Resolution range (Å)	67.14 – 2.30 (2.38 – 2.30)	50.43 – 1.70 (1.73 – 1.70)	66.73 – 1.60 (1.63 – 1.60)	57.81 – 1.9 (1.94 – 1.9)	67.52 – 2.36 (2.49 – 2.36)
Space group	P 21 21 21	P 1 21 1	P 21 21 21	P 65 2 2	P 21 21 21
Cell dimensions					
a, b, c (Å)	58.92, 93.07, 96.94	59.01, 55.66, 84.89	58.79, 92.47, 96.39	66.75, 66.75, 121.21	58.87, 92.20, 99.17
α, ß, γ (°)	90.00, 90.00, 90.00	90.00, 95.15, 90.00	90.00, 90.00, 90.00	90.00, 90.00, 120.00	90.00, 90.00, 90.00
I/σI	11.8 (0.9)	12.1 (1.80)	11.2 (2.20)	20.2 (1.25)	6.1 (1.20)
R_{meas}	0.21 (4.26)	0.09 (1.30)	0.11 (1.02)	0.09 (13.04)	0.29 (1.82)
R_{pim}	0.05 (0.88)	0.04 (0.49)	0.04 (0.32)	0.02 (2.64)	0.11 (0.70)
$CC_{1/2}$	1.00 (0.60)	1.00 (0.74)	1.00 (0.84)	1.00 (0.31)	0.96 (0.41)
Multiplicity	21.0 (22)	6.6 (6.8)	10.3 (9.7)	36.4 (23.9)	6.8 (6.4)
Completeness (%)	99.7 (100.0)	98.0 (99.0)	99.6 (97.1)	99.9 (99.9)	96.3 (94.6)
**Refinement**					
R_{work}/ R_{free}	0.253 / 0.295	0.239 / 0.284	0.212 / 0.234	0.276 / 0.348	0.299 / 0.314
No.atoms					
Protein	3913	3791	3931	921	3848
Water	221	502	436	50	150
Ligand	0	0	50	1	0
B-factors					
Protein	63.82	21.6	45.4	78.1	41.0
Water	59.00	49.9	46.6	71.5	26.6
Ligand	0	0	45.9	65.6	0
R.m.s. deviations					
Bond lengths (Å)	0.011	0.0139	0.012	0.0100	0.0087
Bond angles (°)	1.544	2.0830	1.602	1.7820	1.36
Ramachandran favoured (%)	96.5	97.1	99.0	97.3	98.0
Ramachandran allowed (%)	3.3	2.9	1.0	2.7	2.0
Ramachandran outliers (%)	0.2	0.0	0.0	0.0	0.0
Rotamer outliers (%)	0.5	1.3	0.8	0.0	1.0
Clash score	0.5	1.9	1.3	0.6	2.8
MolProbity score	0.9	1.2	0.85	0.81	1.08

Values in parentheses are for highest resolution shell.

**Data Collection**					
	**NTD_E136D**	**NTD_P151S**	**NTD_Q58I**	**NTD_S105I**	**NTD_Y109A**
PDB	9EVY	9FBG	9F5J	9F7C	9EWH
Resolution range (Å)	84.29 – 1.55 (1.58 – 1.55)	47.75 – 2.54 (2.58 – 2.54)	57.12 – 2.20 (2.27 – 2.20)	41.45 – 2.00 (2.05 – 2.00)	83.74 – 1.93 (1.98 – 1.93)
Space group	P 1 21 1	C 1 2 1	P 21 21 21	P 41 21 2	P 1 21 1
Cell dimensions					
a, b, c (Å) α, ß, γ (°)	58.87, 55.10, 84.59 90.00, 94.82, 90.00	188.11, 108.88, 146.27 90.00, 100.79, 90.00	47.78, 49.72, 114.25 90.00, 90.00, 90.00	58.62, 58.62, 111.59 90.00, 90.00, 90.00	58.84, 55.14, 84.09 90.00, 95.24, 90.00
I/σI	16.0 (3.90)	11.7 (1.60)	25.0 (6.40)	15.8 (1.20)	12.5 (2.98)
R_{meas}	0.06 (0.47)	0.09 (0.95)	0.11 (0.63)	0.07 (1.86)	0.11 (0.67)
R_{pim}	0.03 (0.19)	0.05 (0.52)	0.02 (0.11)	0.02 (0.55)	0.04 (0.23)
CC1/2	1.00 (0.92)	1.00 (0.66)	1.00 (0.99)	1.00 (0.94)	1.00 (0.90)
Multiplicity	5.9 (5.9)	3.5 (3.3)	43.2 (33.7)	12.4 (11.2)	8.2 (8.4)
Completeness (%)	97.6 (97.5)	99.8 (96.4)	99.9 (100)	99.8 (92.0)	98.9 (99.2)
**Refinement**					
R_{work}/ R_{free}	0.223 / 0.230	0.209 / 0.251	0.206 / 0.245	0.241 / 0.275	0.209 / 0.252
No.atoms					
Protein	3936	18516	1894	1046	3932
Water	517	1116	138	52	731
Ligand	0	0	4	2	0
B-factors					
Protein	30.1	50.7	49.5	81.6	29.5
Water	32.5	45.4	49.8	70.5	38.5
Ligand	0	0	98.4	60.7	0
R.m.s. deviations					
Bond lengths (Å)	0.012	0.010	0.0135	0.0107	0.011
Bond angles (°)	1.996	1.396	1.909	2.5610	1.581
Ramachandran favoured (%)	97.8	96.7	97.5	92.4	98.8
Ramachandran allowed (%)	2.2	3.0	2.5	6.8	1.2
Ramachandran outliers (%)	0.0	0.3	0.0	0.8	0.0
Rotamer outliers (%)	0.5	2.1	1.6	1.9	0.2
Clash score	2.5	1.9	2.4	1.4	0.5
MolProbity score	1.08	1.4	1.28	1.56	0.68

Values in parentheses are for highest resolution shell.

Supplementary Table 4: Comparison of {^1H}-^{15}N hetNOE values for finger regions of WT, S105I and Q58I. Given are average hetNOE values for the indicated region. The given errors represent the average error for the respective region, calculated according to equation (4) in the methods section.

average hetNOE comparison	WT	S105I	Q58I
N-loop finger (aa 58 – 63)	0.64 ± 0.02	0.65 ± 0.08	0.50 ± 0.03[a]
hairpin finger (aa 90 – 107)	0.53 ± 0.02 (0.52 ± 0.01[b])	0.45 ± 0.04 [b]	0.44 ± 0.01[b]
carboxy finger (aa 170 – 180)	0.33 ± 0.02	0.31 ± 0.05	0.26 ± 0.02

[a] without residue 58; [b] without residues G97, R107.

Supplementary References

1. Gangavarapu, K. *et al.* Outbreak.info genomic reports: scalable and dynamic surveillance of SARS-CoV-2 variants and mutations. *Nat Methods* **20**, 512–522 (2023).
2. Korn, S. M., Dhamotharan, K., Jeffries, C. M. & Schlundt, A. The preference signature of the SARS-CoV-2 Nucleocapsid NTD for its 5'-genomic RNA elements. *Nat Commun* **14**, 3331 (2023).
3. Vögele, J. *et al.* High-resolution structure of stem-loop 4 from the 5′-UTR of SARS-CoV-2 solved by solution state NMR. *Nucleic Acids Research* **51**, 11318–11331 (2023).
4. Zok, T. *et al.* RNApdbee 2.0: multifunctional tool for RNA structure annotation. *Nucleic Acids Research* **46**, W30–W35 (2018).
5. Jurrus, E. *et al.* Improvements to the APBS biomolecular solvation software suite. *Protein Sci* **27**, 112–128 (2018).
6. Wang, J. *et al.* iCn3D, a web-based 3D viewer for sharing 1D/2D/3D representations of biomolecular structures. *Bioinformatics* **36**, 131–135 (2020).
7. Szelazek, B. *et al.* Structural Characterization of Human Coronavirus NL63 N Protein. *Journal of Virology* **91**, 10.1128/jvi.02503-16 (2017).
8. Fan, H. *et al.* The Nucleocapsid Protein of Coronavirus Infectious Bronchitis Virus: Crystal Structure of Its N-Terminal Domain and Multimerization Properties. *Structure* **13**, 1859–1868 (2005).
