# Supplementary material for: A core network in the SARS-CoV-2 nucleocapsid NTD mediates structural integrity and selective RNA-binding
Source: Nat Commun. 2024 Dec 9;15:10656. doi: 10.1038/s41467-024-55024-0 (PMC11628620; doi:10.1038/s41467-024-55024-0)
Supplement: Supplementary file 2 — Reporting Summary [file 41467_2024_55024_MOESM2_ESM.pdf]

nature portfolio

Corresponding author(s): Sophie M. Korn
Andreas Schlundt

Last updated by author(s): Nov 14, 2024

Reporting Summary

Nature Portfolio wishes to improve the reproducibility of the work that we publish. This form provides structure for consistency and transparency in reporting. For further information on Nature Portfolio policies, see our Editorial Policies and the Editorial Policy Checklist.

Statistics

For all statistical analyses, confirm that the following items are present in the figure legend, table legend, main text, or Methods section.

n/a	Confirmed	
☐	☒	The exact sample size (*n*) for each experimental group/condition, given as a discrete number and unit of measurement
☐	☒	A statement on whether measurements were taken from distinct samples or whether the same sample was measured repeatedly
☐	☒	The statistical test(s) used AND whether they are one- or two-sided<br>*Only common tests should be described solely by name; describe more complex techniques in the Methods section.*
☐	☒	A description of all covariates tested
☐	☒	A description of any assumptions or corrections, such as tests of normality and adjustment for multiple comparisons
☐	☒	A full description of the statistical parameters including central tendency (e.g. means) or other basic estimates (e.g. regression coefficient) AND variation (e.g. standard deviation) or associated estimates of uncertainty (e.g. confidence intervals)
☒	☐	For null hypothesis testing, the test statistic (e.g. *F*, *t*, *r*) with confidence intervals, effect sizes, degrees of freedom and *P* value noted<br>*Give P values as exact values whenever suitable.*
☒	☐	For Bayesian analysis, information on the choice of priors and Markov chain Monte Carlo settings
☒	☐	For hierarchical and complex designs, identification of the appropriate level for tests and full reporting of outcomes
☒	☐	Estimates of effect sizes (e.g. Cohen's *d*, Pearson's *r*), indicating how they were calculated

Our web collection on statistics for biologists contains articles on many of the points above.

Software and code

Policy information about availability of computer code

Data collection	1. Protein crystal diffraction data was collected at P13 beamline at the PETRA III storage ring of the DESY synchrotron and at the Swiss Light Source (SLS) on macromolecular crystallography beamline PXI-X06SA.<br>2. NMR data was acquired using Topspin(v3 and v4).<br>3. Nano Differential Scanning Fluorimetry (nanoDSF) data was obtained using Prometheus Panta (NanoTemper Technologies) instrument at Sample Preparation and Characterization facility (SPC) in Hamburg and in the group of Dr. Melanie McDowell at the Max Planck Institute of Biophysics in Frankfurt.<br>4. Micro scale thermophoresis (MST) data were acquired using Monolith NT.115 instrument (NanoTemper Technologies).<br>5. EMSA gels were scanned using Herolab E.A.S.Y.429K (Herolab GmbH, Germany).<br>6. Fluorescent EMSAs were imaged using Bio-Rad ChemiDoc™ imaging system in the group of Prof. Dr. Stefanie Kaiser at Goethe University Frankfurt.<br>7. RNA secondary structure was plotted using RNArtist (https://github.com/fjossinet/RNArtist).
Data analysis	1. Crystal data were processed using CCP4i2 (v1.1.0).<br>2. Manual model building was performed in Coot (v0.9.8.93).<br>3. Protein structures were visualized using Pymol (v2.5.5) and ChimeraX (v1.8).<br>4. NMR data were processed using Topspin(v3 and v4).<br>5. Backbone assignment, analysis of CSPs and relaxation data were performed in CCPNMR analysis software suite (v2.5 and 3.2) and plotted in Microsoft Excel (v2405).<br>6. nanoDSF based protein melting temperatures were analyzed using MoltenProt server (https://spc.embl-hamburg.de/app/moltenprot).<br>7. MST data was analyzed using PALMIST (v1.5.8) and normalized averages were plotted in OriginPro (v2020b).

8. EMSA gels were analyzed using Herolab E.A.S.Y.429K (Herolab GmbH, Germany).
9. EMSA gels were quantified using ImageQuantTL 10.2 (Cytiva, Germany).
10. Fluorescent EMSA gels were analyzed using Bio-Rad ChemiDoc™ imaging system.
11. PDB code used for construct design are 6YI3 and 6M3M.
12. PDB code used in data analysis are 6YI3, 7CDZ, 54NK, 2BXX, 4J3K, 4UD1, 3HD4 and 2OFZ.
13. Sidechain assignments were transferred based on previous assignments from BMRB entry 34511.

For manuscripts utilizing custom algorithms or software that are central to the research but not yet described in published literature, software must be made available to editors and reviewers. We strongly encourage code deposition in a community repository (e.g. GitHub). See the Nature Portfolio guidelines for submitting code & software for further information.

Data

Policy information about availability of data

All manuscripts must include a data availability statement. This statement should provide the following information, where applicable:

- Accession codes, unique identifiers, or web links for publicly available datasets
- A description of any restrictions on data availability
- For clinical datasets or third party data, please ensure that the statement adheres to our policy

Material requests shall be made to the corresponding author.
1. All PDB data will be accessible at RCSB upon publication and the PDB IDs are
9EXB (NTD_WT) https://doi.org/10.2210/pdb9EXB/pdb
9F83 (NTD_D63G) https://doi.org/10.2210/pdb9F83/pdb
9EZB (NTD_P67S) https://doi.org/10.2210/pdb9EZB/pdb
9F7A (NTD_P80R) https://doi.org/10.2210/pdb9F7A/pdb
9F5L (NTD_A119S) https://doi.org/10.2210/pdb9F5L/pdb
9EVY (NTD_E136D) https://doi.org/10.2210/pdb9EVY/pdb
9FBG (NTD_P151S) https://doi.org/10.2210/pdb9FBG/pdb
9F5J (NTD_Q58I) https://doi.org/10.2210/pdb9F5J/pdb
9F7C (NTD_S105I) https://doi.org/10.2210/pdb9F7C/pdb
9EWH (NTD_Y109A) https://doi.org/10.2210/pdb9EWH/pdb
2. Backbone assignments are deposited at BMRB under the following accession numbers:
52469 (NTD_Q58I) https://doi.org/10.13018/BMR52469
52470 (NTD_Y109A) https://doi.org/10.13018/BMR52470
52471 (NTD_D63G) https://doi.org/10.13018/BMR52471
52472 (NTD_P67S) https://doi.org/10.13018/BMR52472
52473 (NTD_P80R) https://doi.org/10.13018/BMR52473
52474 (NTD_S105I) https://doi.org/10.13018/BMR52474
BMRB depositions can be accessed temporarily until publication at:
https://bmrb.io/author_view/52469_hy_xkakqqix.str
https://bmrb.io/author_view/52470_hy_juntgkoh.str
https://bmrb.io/author_view/52471_hy_scilmmpq.str
https://bmrb.io/author_view/52472_hy_ppqtouhk.str
https://bmrb.io/author_view/52473_hy_jdgbvvol.str
https://bmrb.io/author_view/52474_hy_ztxdlipo.str
3. All NMR spectra underlying the herein presented data will be provided upon request.
4. All raw data used in this study are included in the Source Data file.

Research involving human participants, their data, or biological material

Policy information about studies with human participants or human data. See also policy information about sex, gender (identity/presentation), and sexual orientation and race, ethnicity and racism.

Reporting on sex and gender	n/a
Reporting on race, ethnicity, or other socially relevant groupings	n/a
Population characteristics	n/a
Recruitment	n/a
Ethics oversight	n/a

Note that full information on the approval of the study protocol must also be provided in the manuscript.

Field-specific reporting

Please select the one below that is the best fit for your research. If you are not sure, read the appropriate sections before making your selection.

☒ Life sciences ☐ Behavioural & social sciences ☐ Ecological, evolutionary & environmental sciences

For a reference copy of the document with all sections, see nature.com/documents/nr-reporting-summary-flat.pdf

Life sciences study design

All studies must disclose on these points even when the disclosure is negative.

Sample size	n/a
Data exclusions	n/a
Replication	1. nanoDSF data were collected from three biological replicates each measured in duplicate (N=3).<br>2. MST data were collected from three biological replicates each measured in duplicate(N=3).<br>3. EMSA assays were done with two biological replicates and are included in the Source Data (N=2).<br>4. Biological replicates of 15N isotopically-labeled recombinant protein batches were verified by identical HSQC NMR spectra.<br>5. In vitro transcribed RNA batches (biological replicates) were checked by 1D 1H NMR and by native/denaturing Urea PAGE.
Randomization	Not applicable to this study, since the work does not include any sample or organism that require randomization.
Blinding	Not applicable to this study, since the work does not include any clinical research.

Reporting for specific materials, systems and methods

We require information from authors about some types of materials, experimental systems and methods used in many studies. Here, indicate whether each material, system or method listed is relevant to your study. If you are not sure if a list item applies to your research, read the appropriate section before selecting a response.

Materials & experimental systems

n/a	Involved in the study	
☒	☐	Antibodies
☒	☐	Eukaryotic cell lines
☒	☐	Palaeontology and archaeology
☒	☐	Animals and other organisms
☒	☐	Clinical data
☒	☐	Dual use research of concern
☒	☐	Plants

Methods

n/a	Involved in the study	
☒	☐	ChIP-seq
☒	☐	Flow cytometry
☒	☐	MRI-based neuroimaging

Plants

Seed stocks	*Report on the source of all seed stocks or other plant material used. If applicable, state the seed stock centre and catalogue number. If plant specimens were collected from the field, describe the collection location, date and sampling procedures.*
Novel plant genotypes	*Describe the methods by which all novel plant genotypes were produced. This includes those generated by transgenic approaches, gene editing, chemical/radiation-based mutagenesis and hybridization. For transgenic lines, describe the transformation method, the number of independent lines analyzed and the generation upon which experiments were performed. For gene-edited lines, describe the editor used, the endogenous sequence targeted for editing, the targeting guide RNA sequence (if applicable) and how the editor was applied.*
Authentication	*Describe any authentication procedures for each seed stock used or novel genotype generated. Describe any experiments used to assess the effect of a mutation and, where applicable, how potential secondary effects (e.g. second site T-DNA insertions, mosiacism, off-target gene editing) were examined.*
