# Supplementary material for: A core network in the SARS-CoV-2 nucleocapsid NTD mediates structural integrity and selective RNA-binding
Source: Nat Commun. 2024 Dec 9;15:10656. doi: 10.1038/s41467-024-55024-0 (PMC11628620; doi:10.1038/s41467-024-55024-0)
Supplement: Supplementary file 3 — Transparent Peer Review file [file 41467_2024_55024_MOESM3_ESM.pdf]

nature portfolio

Peer Review File

A Core Network in the SARS-CoV-2 Nucleocapsid NTD Mediates Structural Integrity and Selective RNA-binding

Corresponding Author: Professor Andreas Schlundt

This file contains all reviewer reports in order by version, followed by all author rebuttals in order by version.

Version 0:

Reviewer comments:

Reviewer #1

(Remarks to the Author)
In this comprehensive study carried out by Dhamotharan, et al, they examine and characterize the impact that naturally occurring mutations have on the NTD domain of SARS-CoV-2 and investigate the impact that these mutations have on RNA binding. The approaches used include NMR, Xray crystallography, MST, and EMSAs. The authors provide a structural explanation for the change in binding affinity (or lack thereof) of several different mutant forms of the N protein, and identify a network of residues critical for the proper folding of the N protein, affirmed through mutational analysis, melting point experiments, and NMR CSPs. Impressively, they find that the network of residues contributes to the RNA sequence specificity of N, and provide a structural explanation based off their Xray structures.

The work is significant in that it describes the mechanisms governing the recognition and binding of the SARS-CoV-2 N protein and its RNA targets. The N protein is relatively understudied compared to other components of SARS-CoV-2 (eg S, Mpro, RdRP), so more structural and biochemical data on this particular part of the virus is greatly appreciated. Particularly, the robust structural characterization of N and several different mutants that had not previously been structurally characterized is valuable for the field.

Overall, I the conclusions are solid and are supported by ample data sourced from orthogonal techniques. This is an excellent demonstration of how biochemical and biophysical assays can be combined to investigate the molecular mechanisms that drive biological / viral function. I think this provides not only insight to SARS-CoV-2, but also demonstrates to others in the field a pipeline approach (granted their proteins crystalize and give good NMR spectra) for linking structure to function. An good follow-up to this excellent structural work could be to investigate, in cells, how the mutations impact viral replication. I have some suggestions for improving the text, as well as some comments/questions to help clarify some of the data.

Line 43: Consider revising this sentence:
Systematically combining crystallography and solution NMR, we here investigate the
influence of six naturally occurring plus strategic NTD mutations on structural integrity and RNA-
binding.

Line 58 and 60: use the word viral?:
virus variants

Line 75: Consider revising (the “e.g.” in the middle of the sentence seems out of place):
Those mutations account e.g., for increased transmissibility

Line 80: spell out SR since it is the first time using it:
SR-rich region

Line 82: Consider revising
but among them a few are found to be lineage-defining

Line 83 For someone not familiar with viral proteins, why is this likely?

Line 88/89: add a ref (unless those are what are referenced in the next sentence):
RNA complex formation has been suggested to rely on electrostatics and on stacking interactions mediated by highly conserved palm residues.

Line 93: Is the affinity for random RNA interactions known?

Line 94: do you mean affinity?
newly formed RNP through high-affine interactions

Line 112: Two of these nat_mutants show a slight increase in RNA affinity.
Be sure to come back around to explaining why this occurs.

Line 125: Could the authors comment on the potential impact of mutations outside the structured NTD?

Line 137: Maybe the authors should point out that the large CSP of the residue adjacent to the mutation in A199S compared to E136D can be attributed to the similar chemical nature of E and D in respect to A and S. This could avoid confusion. What is the impact of the E136D mutant? It seems minimally invasive, even though it is lineage defining for VOC omicron?

Figure 1:
Figure 1B: how do you set the threshold for what you consider significant (eg the red-labeled amino acid) vs not (like 136)?
Figure 1's legend does not have a 'c' descriptor.
Figure 1's legend for e: maybe state it is the NTD in addition to residues:
Sequence of the WT NTD, spanning residues 41 to 180
Figure 1 is VERY crowded. Can panels A and B be combined, possibly by putting the sausage plot above the entropy plot? Is panel e (the sequence) necessary to show here? Especially if you have f? I actually think that supplementary figure 1c should replace most of Figure 1 (panel D, E, F and G).

Line 161: for non-experts, state what one qualitatively looks for as an indicator of conserved structure (e.g., the peaks remain well dispersed, and for the most part, that they overlay nearly perfectly with the introduction of mutations)

The spectra in supplementary Figure 2 are way too small. There is no space limitation for the supplementary file. Please make the spectra and corresponding CSP plots bigger. You can trim off some of the regions upfield and downfield that do not have resonances. Also, P67S and P80R have quite a few significant chemical shift perturbations; there are seemingly fewer resonances in the CSP plot that appear above the threshold relative to the spectra. How did the authors pick which resonances to label in the spectra? Why aren't the mutated residues highlighted in the mutant spectra?
Supplementary Fig 2b is not mentioned in the text? Also, what's going on with Q58I? This could use some explaining. I think all of these mutations need some explaining, tbh.

The RNAs SL4ext and RNA Ext – can the authors show a picture of these and/or describe their sequences and 2D structures in the text at the time that they are introduced? Considering this is a comprehensive study of the sequence / structural requirements for binding, this is important. I am curious if the authors attempted to obtain crystal structures of the proteins bound to the RNA, or if they carried out titrations from an RNA point of view? What residues in the RNA are required for binding / facilitate these interactions? Does the stem loop remain intact? Could the authors show their 1D spectra of the RNA (even the 1.2 titration point) of the imino region?

Figure 2:

I would suggest adding the CSP plot for the D63G variant to panel a.
Why not show the site of mutation (the red) in the CSP plots too (panel a)?

Where are the fitted curves for the binding shift assays shown in Figure 2b (as well as Supplementary Figure 5a)? The RNA control sometimes shows two bands; what is the second species? And how do the authors account for this in the fitting of their data? And, how do the fitted binding curves from the EMSAs correlate with the MST data?

Panel b. Some mutants show an additional band for 0 concentration of protein. This is peculiar if the same RNA was used for all EMSA experiments. For some variants there is a clear intermediate band visible before the final complex is formed. Could the authors comment on this?

Line 188: please consider starting a new paragraph for the NMR data for the "We further investigated…" sentence.

In Supplementary Figure 4, what is the threshold for CSPs? This should be added. I have the same comment for Supplementary Figure 5.

Line 317:
W108 in F171G seems broadened in the 15N dimension. Do the structures provide a potential explanation for this dynamic?

Line 338: 0.64 (WT average value) to 0.50 for Q58I, and from 0.52 to 0.44 for hairpin residues 90-105

The error should be listed here.

Figure 5:
Panel a. SL4 non-target plot uses different symbols for legend and plot. Could the authors comment on the unexpected behaviour observed for the last two data points for S105I. Were these points removed from the fitting process?
Panel g. The geometry between F171 and W108 seems not ideal for pi interactions?

Figure 6:
I find this figure confusing. The explanation of colours is not straightforward. For example, does light blue (e.g. R107) mean binding affinity is changed when this residue is changed? What is the significance of the blue stripes in the green bar? Significance of colour combinations (e.g. does blue+green signify residues that are important for binding and are conservative for the fold?) is not obvious. The fact that wedges are used for stability, affinity and RNA differentiation makes the reader expect some kind of gradient to be shown, which to my understanding is not the case. Having a classic legend showing the significance of each colour could be beneficial.

Line 567: in detail instead of in details?

Line 568: Do the structures provide a rationale why Q58I could only be solved in different boundaries?

I think it would be interesting, if possible, to describe the lineage of the variants with the binding affinity. Like, were there less disruptive mutations longer sustained mutations detected in the human population as COVID evolved?

Some other comments:

I do find myself wanting a little more from certain sections. I think the manuscript would be much stronger if the authors were able to get structures of N in complex with their RNA constructs, but I recognize that may be difficult / impractical -- although mention of if they attempted and any struggles they may have faced would be welcome. I find the conclusions the authors make are very much supported by their data, which is good. I more wish the authors took a few extra steps. The authors report D63G and P80R increase binding affinity of N for RNA, and offer a structural explanation for it -- which I found agreeable. Are these mutations seen at all in sequence data from clinical populations? Why do the authors think SARS-CoV-2 hasn't evolved these mutations naturally? would there be some disadvantage associated with increasing RNA binding affinity through mutating these residues?

I would have also liked to see more analysis by the authors on how the Q58I and / or S105I mutants contribute to RNA specificity. I found it interesting that these mutations disrupted the fold of the B-hairpin -- but the connection between the misfold and RNA specificity was lacking in my opinion. Again, without structures of RNA-bound N it is difficult to make more conclusions with the data the authors present, but more experiments pursuing this line of thought would have been appreciated and resulted in a much stronger work. Even perhaps a hypothesis of how mutations in this region disrupt specificity would be welcome. Especially since one mutation (Q58I) completely disrupts the core network while the other (S105I) does not, yet they have similar binding affinities for SL4 compared to the WT. This was, in my opinion, the weakest part of the work.

Discussion or analysis on how these mutations might affect N oligomerization / packing would have been welcomed, but I recognize it's beyond the scope of the authors current intents.

All that said, I do think the work is an extremely valuable contribution to the field and is very robust and thorough.

Reviewer #2

(Remarks to the Author)
A lot of attention was given to every mutation in the Spike protein in every SARS-CoV-2 variant of concern. However, the N protein, probably the second most important coronaviral protein (for instance, all antigen tests are based on the N protein) was never analyzed in such detail and, finally, Dhamotharan and Korn et al. fill the gap. This study represents careful analysis of the naturally occurring mutations in the N-protein and given the socioeconomic importance of CoV-2, it deserves to be published in Nat. Comm. I have few suggestions for improvement.

1) Dhamotharan et al. write that “The N-terminal domain (NTD) was suggested to mediate specific RNA-interactions, but the lack of high-resolution structures with viral RNA prohibits insights into the precise mechanism of complex formation.”

An NMR structure with ssRNA and dsRNA was published and Dhamotharan et al. cite that paper. So this sentence, perhaps, should be reworded.

2) In Figure 2 all the mutants are analyzed using gel shift assay. But the gels are not quantified and the Kd was determined only for half of the mutantss. The Kd needs to be determined for all mutants analyzed.

3) Binding affinities to the RNA Ext are presented for the mutants and wt in Fig. 3. I would be very informative to also use control RNA, such as scrambled RNA Ext.

4) “Mutations within this interface impact NTD RNA-binding, while no structural explanation for this has been provided yet.”

Not really, the NMR structure of the NTD in complex with RNA can explain the impact of most of these mutations.

PDB id 7ACT actually shows that the residue Y109 makes a hydrogen bond with the RNA backbone. The mutant Y109A obviously cannot.

5) “...the three-dimensional fold of the NTD depends on the conserved triple residue network (Q58-W108-F171), connecting the beta sheet core with the adjacent N- and C-terminal fingers.”

If this would be true, than these residues would be absolutely conserved. But they are not conserved between alpha and beta coronaviruses and yet the N NTD appears to have the same fold in alpha coronaviruses. For instance PDB id 5N4K. Although later in the manuscript, the differences between alpha- and beta- coronavirus NTDs are discussed, the statement above seems too strong. Should be at least rewritten as”

“...the three-dimensional fold of the NTD of betacoronaviruses depends on the conserved triple residue network (Q58-W108-F171), connecting the beta sheet core with the adjacent N- and C-terminal fingers.”

.

Reviewer #3

(Remarks to the Author)
Among them, R203(K/M) and G204R, located in the SR-rich region, are most prevalent and associated with an increased viral load and fitness12–14.
However, a comprehensive understanding of the NTD RNA-binding mechanism is still missing, despite some efforts of gaining structural information on RNP.

Interestingly,though widely used as a biological tool to mute NTD RNA-binding in a fl-N context23,28, no structural characterization of this des_mutant existed. We here solved the Y109A crystal structure and can
show that structural integrity is retained in this mutant.

Analysis of the CSP plots for each residue in the ds-14mer titration with this distinction in mind reveals a cluster of residues around D81 and I146, including the helix at residues 79–82, residues 119 and 120, and residues 143–146 (Fig. 4h, Fig. S7). As the CSPs at this site differ substantially from those near the primary binding site, we conclude that this shift corresponds to a second, weak binding surface of the NTD.

Fig 2: a. P80R no colors on the structure, b. is covering structures in e,

The manuscript by Dhamotharan et al is focused on elucidating the effect of naturally occurring and designed mutations on the structure of the NTD of SARS-CoV2 and on identifying the structural features that contribute to specific interactions with RNA. In the process they determined crystal structures of 9 mutants, their effect on CSP by NMR, and compared their thermal stability and RNA binding affinities to WT. Naturally occurring mutations were chosen based on their prevalence in variants of concern. Designed mutants were chosen based on earlier reports of loss of binding to RNA and from examining long range contacts in the crystal structure, and their presence at the RNA primary site and based on conservation among alpha and beta coronaviruses. The work is solid, thorough, uses both NMR and crystallography and multiple other biophysical tools on a large number of mutants. Data are well presented, and the manuscript is clearly written. However, much of the results are expected with no major insights provided as describe below.

1. The 6 naturally occurring mutant NTD showed similar fold to the WT and similar binding affinity to RNA, except for two that showed higher affinity due to changes in their electrostatic contributions. This result is expected. It is rare in proteins that a single mutation will change the overall fold and small changes in affinity are rarely associated with changes in structure. The NT structure has been extensively studies. Structures of the NTD have been determined by multiple techniques (solid state NMR, solution NMR, crystallography) and therefore methods for purification and crystallization are available making this investigation pretty straightforward.
2. For these same mutants, the CSPs were different but not much was made of what this observation implies.
3. Designed mutants chosen at the RNA primary site reduce RNA binding like Y109A and R107A but those have been reported earlier. NMR characterization of structure and dynamics and RNA binding are published for Y109A (Estelle et al.,

PNAS Nexus, 2023). What is new here is the crystal structure but the conclusion is the same as in Estelle et al, that there is no change in structure and that there is some weakened binding rather than total loss of binding.
4. The most interesting part of this manuscript is in the third set of mutants referred as intradomain contact mutants, and their effect on the structure and selectivity of RNA recognition. These mutants drastically affected the fold with highest RMSD compared to WT and therefore their effect on binding is expected. However, what is interesting here is identifying the prpotein residues that contribute to the difference in specificity of binding between the two RNA segments studies SL4 and SL4-Ext. Still some lingering questions should be addressed as below:
a. Why weren't the SL4 and SL4-Ext tested with the naturally occurring mutants or the Y109A?
b. Why wasn't a structure for F171G determined, while there are structures for the two other mutants in the network.
c. What is the rationale for mutations to G and to I. While it was clear why these specific residues were mutated, it was not clear why to G and I.
d. There is no discussion of why Ext is a better binder, is it because of nucleotide specificity or because of its single stranded structure. If the latter then this supports the observation reported in Estelle et al, 2023 of higher specificity to single stranded RNA over double stranded. There was no mention about this in the manuscript.

Reviewer #4

(Remarks to the Author)
I co-reviewed this manuscript with one of the reviewers who provided the listed reports as part of the Nature Communications initiative to facilitate training in peer review and appropriate recognition for co-reviewers.

Reviewer #5

(Remarks to the Author)
I co-reviewed this manuscript with one of the reviewers who provided the listed reports as part of the Nature Communications initiative to facilitate training in peer review and appropriate recognition for co-reviewers.

Version 1:

Reviewer comments:

Reviewer #1

(Remarks to the Author)
I want to thank the authors for thoroughly addressing each and every comment (from all reviewers). I only have one minor comment:

Figure 3D -- just color that alanine differently so it is easier to see.

Reviewer #2

(Remarks to the Author)
The manuscript by Dhamotharan and Korn significantly improved during revisions and can be published as it is although I have few suggestions below. Please, treat these as only suggestions, I do not want to delay the publication process any further. Up to the authors and the editor if they use these.

1) In the mean time, a high resolution structure of the NTD bound to DNA aptamer was published by Esler et al in NAR. This could be perhaps discussed or at least mentioned.

2) The N-terminal domain (NTD) was suggested to mediate specific RNA-interactions, but the lack of high-resolution structures with viral RNA prohibits insights into the precise mechanism of complex formation."

I still believe that this sentence is misleading, it overestimates novelty of this study. It could perhaps state: "The N-terminal domain (NTD) was suggested to mediate specific RNA-interactions, but the lack of high-resolution structures with viral RNA prohibits insights into the precise mechanism of complex formation although some information was gained from hybrid structures of the NTD in complex with ss and dsRNA."

Reviewer #4

(Remarks to the Author)
I co-reviewed this manuscript with one of the reviewers who provided the listed reports. This is part of the Nature Communications initiative to facilitate training in peer review and to provide appropriate recognition for Early Career Researchers who co-review manuscripts.

Reviewer #5

(Remarks to the Author)
I co-reviewed this manuscript with one of the reviewers who provided the listed reports. This is part of the Nature Communications initiative to facilitate training in peer review and to provide appropriate recognition for Early Career Researchers who co-review manuscripts.

Open Access This Peer Review File is licensed under a Creative Commons Attribution 4.0 International License, which permits use, sharing, adaptation, distribution and reproduction in any medium or format, as long as you give appropriate credit to the original author(s) and the source, provide a link to the Creative Commons license, and indicate if changes were made.
In cases where reviewers are anonymous, credit should be given to 'Anonymous Referee' and the source.
The images or other third party material in this Peer Review File are included in the article's Creative Commons license, unless indicated otherwise in a credit line to the material. If material is not included in the article's Creative Commons license and your intended use is not permitted by statutory regulation or exceeds the permitted use, you will need to obtain permission directly from the copyright holder.
To view a copy of this license, visit https://creativecommons.org/licenses/by/4.0/

We sincerely thank all reviewers for their constructive and valuable feedback, and we are pleased with the overall positive reception of our work. Below, we provide a point-by-point response to each of the reviewers' comments.

Of note, we have **made adjustments to the following Figures** (in alignment with reviewer comments):

Figure 1 (rearrangement and fusion of panels)
Figure 2 (addition of D63G to panel a, realignment of EMSAs in panel b, addition of P67S MST curve and changing of individual symbols in panel d)
Figure 3 (changing of individual symbols in panel c)
Figure 4 (Tm plot in panel b, adjusted spectral processing in panel c, adjusted CSD plots in panel d)
Figure 5 (changing of individual symbols and addition of biological replicates in panel a, updating panels b and c based on biological replicates in a)
Figure 6 (reduced complexity)

Supplementary Figure 2 (increased panel sizes and adjusted spectral processing in a and b)
Supplementary Figure 4 (increased plot sizes, included CSP plots for SL4 titration and P67S for Ext and SL4, included thresholds in CSP plots)
Supplementary Figure 5 (added EMSA titration scheme in panel a, included CSP plots for SL4 titration and thresholds for panel b)
Supplementary Figure 7 (updated Tm comparison with additional biological replicates in panel b)
Supplementary Figure 9 (adjusted axis labelling in panel c)
Supplementary Figure 10 (adjusted the arrangement of layers in panel b – WT now on top of mutant, for better visibility)
Supplementary Figure 11 (included thresholds for panel a)

Of note, we have **included new Figure panels** (to support our responses to reviewer comments, as depicted in detail at the respective response site):

Supplementary Figure 2 panel c (representation of RNA secondary structure)
Supplementary Figure 11 panel b (MST fits for S105I/SL4 binding)
Supplementary Figure 11 panel c (EMSAs for WT, Q58I and S105I to SL4)

REVIEWER COMMENTS

Reviewer #1 (Remarks to the Author):
In this comprehensive study carried out by Dhamotharan, et al, they examine and characterize the impact that naturally occurring mutations have on the NTD domain of SARS-CoV-2 and investigate the impact that these mutations have on RNA binding. The approaches used include NMR, Xray crystallography, MST, and EMSAs. The authors provide a structural explanation for the change in binding affinity (or lack thereof) of several different mutant forms of the N protein and identify a network of residues critical for the proper folding of the N protein, affirmed through mutational analysis, melting point experiments, and NMR CSPs. Impressively, they find that the network of residues contributes to the RNA sequence specificity of N, and provide a structural explanation based off their Xray structures.

The work is significant in that it describes the mechanisms governing the recognition and binding of the SARS-CoV-2 N protein and its RNA targets. The N protein is relatively understudied compared to other components of SARS-CoV-2 (eg S, Mpro, RdRP), so more structural and biochemical data on this particular part of the virus is greatly appreciated. Particularly, the robust structural characterization of N and several different mutants that had not previously been structurally characterized is valuable for the field.

Overall, I the conclusions are solid and are supported by ample data sourced from orthogonal techniques. This is an excellent demonstration of how biochemical and biophysical assays can be combined to investigate the molecular mechanisms that drive biological / viral function. I think this provides not only insight to SARS-CoV-2, but also demonstrates to others in the field a pipeline approach (granted their proteins crystalize and give good NMR spectra) for linking structure to function. A good follow-up to this excellent structural work could be to investigate, in cells, how the mutations

impact viral replication. I have some suggestions for improving the text, as well as some comments/questions to help clarify some of the data.

We appreciate the valuable and positive feedback of reviewer #1 regarding our work, and the detailed suggestions for improving the manuscript further.

Line 43: Consider revising this sentence:
Systematically combining crystallography and solution NMR, we here investigate the influence of six naturally occurring plus strategic NTD mutations on structural integrity and RNA-binding.

We revised the sentence to the following:
'*We used crystallography and solution NMR to investigate the influence of mutations in the NTD on structural integrity and RNA-binding.*'

Line 58 and 60: use the word viral?: virus variants

We changed the term 'virus variants of concern (VOC)' to 'variants of concern (VOC)', and 'virus variants' to 'variants'.

Line 75: Consider revising (the "e.g." in the middle of the sentence seems out of place): Those mutations account e.g., for increased transmissibility

We decided to keep the 'e.g.', since we are only giving one example of what these mutations may influence.

Line 80: spell out SR since it is the first time using it:
SR-rich region

We thank the reviewer for noticing and introduced the abbreviation accordingly.

Line 82: Consider revising but among them a few are found to be lineage-defining

We changed the sentence to: *'are less frequent, but some are found to be lineage-defining'*.

Line 83: For someone not familiar with viral proteins, why is this likely?

We agree with the reviewer that this statement needs adjustment. Our intention was to highlight that mutations in the folded RNA-binding domain are likely to lead to more nuanced functional changes compared to those in intrinsically disordered regions (IDRs), where functionality is primarily influenced by altered electrostatic potential. We have revised the sentence to:

'Mutations in the folded NTD will likely have a more complex impact on Nucleocapsid functionality than those in the neighbouring IDRs and thus, require our detailed examinations.'

Line 88/89: add a ref (unless those are what are referenced in the next sentence):
RNA complex formation has been suggested to rely on electrostatics and on stacking interactions mediated by highly conserved palm residues.

Indeed, the respective citations are combined with those cited after the next sentence.

Line 93: Is the affinity for random RNA interactions known?

Affinity for random RNAs varies depending on RNA size and buffer conditions. In our previous publication (Korn et al., 2023, Nature Communications), we reported that affinities for target versus random RNAs of similar size can differ by a factor of 100 (e.g., 0.5 µM vs 50 µM). We confirmed the non-specific nature of RNA interactions by increasing salt concentrations, which abolished non-specific interactions while maintaining specific RNA-binding. While we have chosen not to include affinity values

for random RNAs in the text, due to the variability introduced by multiple factors, we added the following sentence to the introduction to support our statement:
‘Indeed, a number of studies have shown binding of NTD to non-viral model RNAs as proxys for single-stranded, double-stranded, and/or transiently structured motifs’, along with respective references.

Line 94: do you mean affinity?
newly formed RNP through high-affine interactions

Indeed. We thank the reviewer for pointing us at this and corrected the word.

Line 112: Two of these nat_mutants show a slight increase in RNA affinity. Be sure to come back around to explaining why this occurs.

Based on our data, we suggest the increased affinity of these mutants to arise from subtle changes in intramolecular interactions, possible change in sidechain flexibility (from line 223) and changes in surface charge (from line 480).

Line 125: Could the authors comment on the potential impact of mutations outside the structured NTD?

Mutations outside the structured NTD cluster mainly in the surrounding IDRs. To make that fact clearer, we revised the sentence, which now reads:

‘*Along with multiple mutations in the SARS-CoV-2 N protein outside the structured domains, which cluster in the IDRs, several naturally occurring mutations (nat_mutants) have also emerged in the folded NTD (Fig. 1a and b)*.’

With the revised Figure 1 (see our response below regarding Figure 1), the reference to IDR clustering is now much clearer than before.

Line 137: Maybe the authors should point out that the large CSP of the residue adjacent to the mutation in A199S compared to E136D can be attributed to the similar chemical nature of E and D in respect to A and S. This could avoid confusion.

We agree with the reviewer that the statement could potentially be confusing to non-NMR readers and are grateful for the constructive NMR-related feedback. We adjusted the sentence to clarify. We removed ‘magnitude’ and ‘small’ from the sentence, to shift the focus towards the ‘local’ effect around the site of mutation. Additionally, we replaced the term *CSP* (chemical shift perturbation) for the evaluation of mutants in comparison to WT to *CSD* (chemical shift difference), to make it easier to differentiate throughout the text between mutant-vs-WT spectral changes as compared to RNA-induced spectral perturbations (CSP). The sentence is now written as follows:

‘*We found that nat_mutants could–as a proxy–be divided into two groups according to the distribution of CSDs: NTDs A119S, E136D and P151S show exclusively local effects induced by their respective mutations, while P67S, D63G and P80R display more pronounced and long-range CSDs (Fig. 2a and Supplementary Fig. 2a)*.’

What is the impact of the E136D mutant? It seems minimally invasive, even though it is lineage defining for VOC omicron.

The conservative exchange E136D might have manifested in Omicron without having a positive or negative effect on functionality and vitality on the protein level. In contrast to the lineage defining mutants (D63G, P80R and A119S), E136D is found as a prevalent mutation in PANGO lineage BE.1.1 and did not become lineage-defining.

Figure 1:
Figure 1B: how do you set the threshold for what you consider significant (eg the red-labeled amino acid) vs not (like 136)?

We apologize for the caption not being detailed enough to clarify those questions. The red-labeled amino acid (D63G) is given as a point of orientation, indicating the highest entropy value inside the NTD boundaries, with a value of 0.46. It is not indicating a level of significance, and no threshold was used for mutant selection. Indeed, mutant selection was based on either, a characterization as lineage-defining (D63G, P80R and A119S), or, being a prevalent mutation in the VOC omicron (as described in detail in the methods section: Selection of NTD nat_mutants).

We included the following sentence in the Figure legend:

'*For rationale of mutant selection, see methods section*.'

Figure 1's legend does not have a 'c' descriptor.

We thank the reviewer for noticing. After rearrangement of Figure 1, all descriptors are now included in the legend.

Figure 1's legend for e: maybe state it is the NTD in addition to residues:
Sequence of the WT NTD, spanning residues 41 to 180

Figure panel e does no longer exist in that context (in line with the reviewer's suggestion of Figure 1 adjustments, see below).

Figure 1 is VERY crowded. Can panels A and B be combined, possibly by putting the sausage plot above the entropy plot? Is panel e (the sequence) necessary to show here? Especially if you have f? I actually think that supplementary figure 1c should replace most of Figure 1 (panel D, E, F and G).

Indeed, panels in Figure 1 can be represented more compact. We provide an adjusted Figure 1 in our re-submission, following the reviewer's suggestions.

Line 161: for non-experts, state what one qualitatively looks for as an indicator of conserved structure (e.g., the peaks remain well dispersed, and for the most part, that they overlay nearly perfectly with the introduction of mutations)

We clarified that sentence as follows:

'In line with the unaltered RNA-binding behavior of these nat_mutants, we assumed a retained structure based on their NMR fingerprint spectra. Well-dispersed HSQC peak patterns overlaid largely with that of the WT spectrum, suggesting a merely local or less apparent impact of natural mutations on the NTD fold.'

The spectra in supplementary Figure 2 are way too small. There is no space limitation for the supplementary file. Please make the spectra and corresponding CSP plots bigger. You can trim off some of the regions upfield and downfield that do not have resonances.
Also, P67S and P80R have quite a few significant chemical shift perturbations; there are seemingly fewer resonances in the CSP plot that appear above the threshold relative to the spectra. How did the authors pick which resonances to label in the spectra? Why aren't the mutated residues highlighted in the mutant spectra?

We thank the reviewer for carefully going through the manuscript and the Supplementary Information and pointing out these questions. Following the suggestions of the reviewer, we increased the size of panels in Supplementary Fig. 2, making spectra and difference plots clearer and more comprehensible. We additionally indicated the site of mutation in the difference plots and labelled significantly shifted resonances (CSD above the threshold) for the natural mutants. We do not label any residues in the des_mutant spectra, as it would be too crowded due to the large number of changed residues.

The HSQC spectra used for the qualitative overlay in the Supplementary Information had not been adjusted to different acquisition parameters (concentration and number of scans) and processing. We corrected that short-coming in that we now overlaid all spectra from samples of same concentration and acquisition parameters, processed identically (and recorded at the same spectrometer).

The question raised by the reviewer regarding the CSD threshold not perfectly reflecting the actual number of significantly perturbed peaks is understandable. The impression arises from a number of sidechains being perturbed that do not go into the CSD plot. We stated that in the Figure legend (Supplementary Fig. 2) to prevent confusion. Please also note that we use a standard formula to calculate the combined CSP/CSD (see method section), in which ^{1}H and ^{15}N dimensions are differently weighted, such that the visual impression may not always transfer into an expected subsequent number.

Supplementary Fig 2b is not mentioned in the text?

We thank the reviewer for noticing. This has been corrected (see tracked changes).

Also, what's going on with Q58I? This could use some explaining. I think all of these mutations need some explaining, tbh.

The large changes in the spectrum of Q58I compared to the NTD WT reflect the significant effect that this single-residue exchange has on the structural integrity of the domain, which is in line with the impact we observe for F171G (of the core network) in the respective HSQC in Supplementary Fig. 9b. We discuss these effects in the respective chapter, e.g., from lines 354 on. We decided to include the des_mutant spectral overlays with WT (and respective CSD plots) in Supplementary Fig. 2, together with the nat_mutants, for convenient clustering of those data sets, but only discuss des_mutant effects on the NMR-level throughout later passages.

The RNAs SL4ext and RNA Ext – can the authors show a picture of these and/or describe their sequences and 2D structures in the text at the time that they are introduced? Considering this is a comprehensive study of the sequence / structural requirements for binding, this is important.

Including the RNA secondary structures is indeed useful and important. We included the secondary structures of all used RNAs in **Supplementary Fig. 2 c**.

I am curious if the authors attempted to obtain crystal structures of the proteins bound to the RNA, or if they carried out titrations from an RNA point of view.

Regarding complex structures of the NTD and RNA: we absolutely agree that a high-resolution RNP structure would be desirable. We (and many other groups) have extensively tried that with various RNA targets and in numerous rounds of optimization, but–likely due to the dynamics of both RNA and NTD domain–were unsuccessful so far. Also see our more detailed response below!

What residues in the RNA are required for binding / facilitate these interactions? Does the stem loop remain intact? Could the authors show their 1D spectra of the RNA (even the 1.2 titration point) of the imino region?

In our previous publication (Korn et al., 2023, Nature Communications) we intensively studied the target RNA preferences of the SARS-CoV-2 NTD, which is also the origin of the target/non-target RNA elements we used in this study here. In Korn et al, we screen various viral RNAs and find a preference of the NTD towards labile stem-loops (such as 'Ext'). We use NMR, ITC/EMSA and SAXS to characterize RNA-binding to a set of RNAs. In that study, we also show titrations of NTD to the RNA, which clearly demonstrated the NTD to capture the ssRNA moiety in Ext, rather than binding dsRNA. That study also includes extensive analysis of RNA 1D and 2D spectra, incl. multiple temperatures and CSP mappings for the mentioned RNAs. We there (mainly in the SI) discuss binding of the NTD to the various RNAs, esp. Ext and SL4ext, which fully covers this reviewer's questions. However, as we agree that the choice of RNA used in that study should be explained a bit better, we decided to include–at the site of first mention–a short paragraph to introduce the SL4ext RNA target (lines 171-175).

Apart from this, we would like to state again that this time we here concentrate on NTD mutants and thus chose an established RNA target/non-target pair for their individual analyses.

Figure 2:
I would suggest adding the CSP plot for the D63G variant to panel a.
Why not show the site of mutation (the red) in the CSP plots too (panel a)?

We have added D63G to the main text Fig. 2a, and indicated the site of mutation.

Where are the fitted curves for the binding shift assays shown in Figure 2b (as well as Supplementary Figure 5a)? The RNA control sometimes shows two bands; what is the second species? And how do the authors account for this in the fitting of their data? And how do the fitted binding curves from the EMSAs correlate with the MST data?
Panel b. Some mutants show an additional band for 0 concentration of protein. This is peculiar if the same RNA was used for all EMSA experiments. For some variants there is a clear intermediate band visible before the final complex is formed. Could the authors comment on this?

Regarding quantification of the EMSAs provided in this study: initial categorization of nat_mutants has been carried out on the basis of qualitative EMSAs, to compare mutant RNA-binding to that of the NTD WT (with respect to complex formation and the approximate order of magnitude of K_D values). Since EMSAs have been carried out with non-labelled RNA (requiring a minimum of 3µM RNA in the herein used volumes to be visualized), our experimental setup does not allow to reliably derive K_D values, since we are close to or above the expected K_D for the binding events (Korn et al., 2023, Nature Communications). We thus believe, fitting these qualitative EMSAs is not useful, and they rather should be considered–as intended–an approximate classification of interactions and the quantification of affinities to be carried out with a more suitable methodology (which we do by MST).
For clarification, we provide below–as an example–the fitted curve for our EMSA of SL4ext when titrated with NTD WT. The calculated K_D is in the range of 2µM, and thus around ten-fold increased when compared to an ITC-derived value (Korn et al., 2023, Nature Communications). This apparent lower affinity of the EMSA is derived from the region of transition (important for K_D calculation), which is extremely steep (large error) and insufficient for reliable fitting.

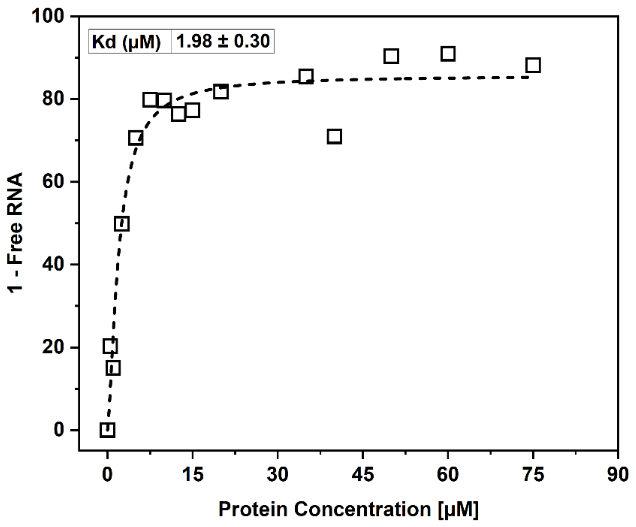

Quantification of NTD WT binding to SL4ext from an exemplary EMSA (compare to main text Fig. 2b).

The second band observed in EMSAs with SL4ext likely represents an alternative RNA conformation under the native gel conditions (see the comparison of SL4ext vs. SL4 and Ext on a native gel, added below), as commonly observed for larger RNAs (i.e., here, >60nt). Denaturing gels of the RNA used for EMSAs clearly exclude their degradation as they show one single band (shown below). Indeed, we see both bands in all native EMSA gels, at varying intensity among replicates (see source data for repetitions). The same RNA was used within the EMSA experiments, so we can assume that both RNA conformations exist in all EMSA experiments (possibly differing slightly in the percentage of population). Since we do not fit EMSAs to obtain K_D values, we neglect the existence of a second minor conformation for our qualitative analysis of complex formation. Important in this context: The MST-derived fits have been obtained for Ext RNA (which is a smaller portion of the SL4ext RNA, and in fact the portion that is preferentially bound), which shows just one conformation under native gel conditions (see below).

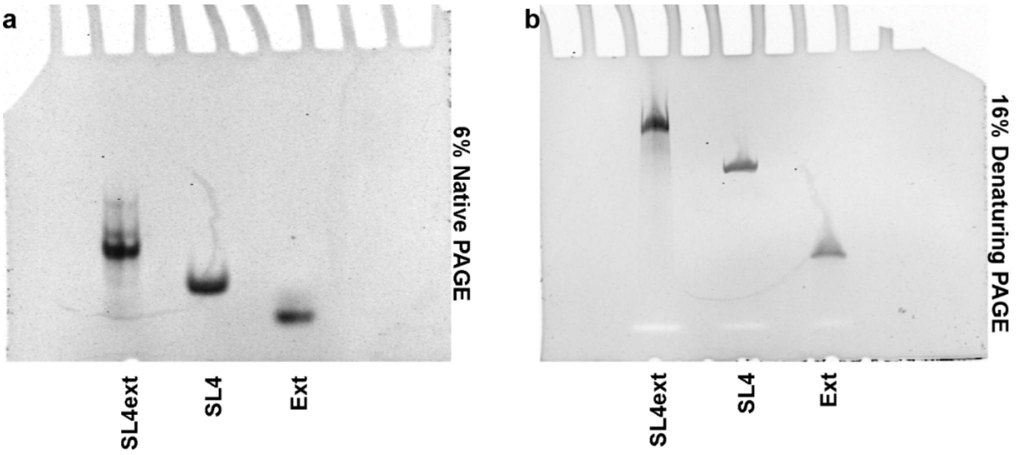

RNAs of this study run on a native polyacrylamide gel (a) or a denaturing polyacrylamide gel (b).

Regarding the intermediate complex band in EMSAs: we correlate this band at sub-stoichiometric concentrations to the initial and high-affinity binding of one NTD molecule to the Ext moiety in SL4ext. At higher concentrations, additional NTD-binding to the SL4 moiety co-occurs (with the described and confirmed lower affinity for that element, (Korn et al., 2023, Nature Communications)), increasing the MW of the RNP complex. To circumvent any complications regarding the quantification of K_D values, we decided to use Ext RNA in MST experiments that follows a pure 1:1 binding.

Line 188: please consider starting a new paragraph for the NMR data for the "We further investigated…" sentence.

We started a paragraph as suggested.

In Supplementary Figure 4, what is the threshold for CSPs? This should be added. I have the same comment for Supplementary Figure 5.

Absolutely justified comment! We included the CS difference thresholds (average + 1SD) for all respective plots, and provide an explanation in Figure legends, accordingly.

Line 317:
W108 in F171G seems broadened in the 15N dimension. Do the structures provide a potential explanation for this dynamic?

The observed differences in the 15N dimension for the Trp sidechains in Fig. 4c and Supplementary Fig. 9b were caused by the non-identical processing of overlaid spectra, which had not been taken care before first submission. We apologize for that flaw. We have adjusted processing to be the same in all HSQC overlays, including Supplementary Fig. 9b and Fig. 4c, to avoid the misleading effect of differential line-broadening. Thus, there is no role for a particular difference in dynamics here.

Line 338: 0.64 (WT average value) to 0.50 for Q58I, and from 0.52 to 0.44 for hairpin residues 90-105
The error should be listed here.

Errors have been included.

Figure 5:
Panel a. SL4 non-target plot uses different symbols for legend and plot.

Symbols in the SL4 plot have been adjusted.

Could the authors comment on the unexpected behavior observed for the last two data points for S105I. Were these points removed from the fitting process?

The data points from MST binding experiments for S105I to SL4 RNA suggest a second, low-affinity binding event at high protein concentrations (K_D > 0.5 mM). A similar trend was observed for Q58I, though at even higher concentrations (not shown). To better visualize the binding of S105I, Q58I, and WT at non-physiologically high concentrations, we performed EMSAs with fluorescently labeled SL4 RNA, which we have included in the new **Supplementary Fig. 11c**. The EMSAs reveal that beyond a concentration of 40 µM, both mutants appear to form higher-order oligomeric complexes.
To assess the impact of the secondary low-affinity binding of S105I on the calculation of the high-affinity binding event, presumably at 1:1, we compared different analysis models (**Supplementary Fig. 11b**).

1. Fitting both binding events independently using a one-site Hill model yielded K_D 1 6.7 µM (± 1.47 µM) and K_D 2 > 500 µM.
2. A biphasic fit (BiHill model) resulted in K_D 1 6.61 µM (± 0.96 µM) and K_D 2 498 µM (± 170 µM).

Both models produced highly consistent K_D 1 values for the first binding event, indicating that the second, low-affinity binding event is independent and does not affect our calculations.
While the behavior of these mutants at high protein concentrations is an intriguing feature that warrants further investigation into the mechanistic effects of these particular NTD mutants, we do not expect it to influence the conclusions of this study. Note that those findings only became obvious from very high, non-physiological stocks of protein samples and harbor the risk of oligomer-caused artifacts.

Panel g. The geometry between F171 and W108 seems not ideal for pi interactions?

We agree that it is not an ideal π-π stacking interaction. The orientation and the centroid distance between the phenyl ring of F171 and the indole ring of W108 allows a T-shaped π-π interaction in an edge-to-face conformation as also observed in other studies (PMID: 29308120; PMID: 26370211: see here Fig. 6 for an angle/distance-related classification). As an example, we show below the interaction between W108 and F171 in our NTD_WT structure. The two centroids are shown as red spheres, and the dotted line represents the distance between them. While not ideal, these two planar rings do exhibit a, albeit weakened, π-π stacking interaction, also supported by additional interactions with adjacent sidechains that keep the rings in place, e.g. L64 and Q58 as shown in Suppl. Fig. 8.

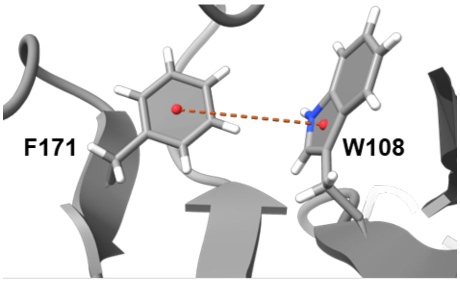

**Centroid distance**	**5.47 Å**
**Angle**	**62.94°**

An exemplary zoom-in into NTD WT structure (9EXB chain C) showing the orientation of W108 and F171.

Figure 6:
I find this figure confusing. The explanation of colours is not straightforward. For example, does light blue (e.g. R107) mean binding affinity is changed when this residue is changed? What is the significance of the blue stripes in the green bar? Significance of colour combinations (e.g. does blue+green signify residues that are important for binding and are conservative for the fold?) is not obvious. The fact that wedges are used for stability, affinity and RNA differentiation makes the reader expect some kind of gradient to be shown, which to my understanding is not the case. Having a classic legend showing the significance of each colour could be beneficial.

We understand that seemingly too many layers of complexity destroyed our intention of providing a visual summary of the findings. We now provide an updated version with reduced complexity (and numbers of colors) and hope to meet the expectations of a summary figure.

Line 567: in detail instead of in details?

Has been changed.

Line 568: Do the structures provide a rationale why Q58I could only be solved in different boundaries?

In the end, we can only speculate about this as the success of crystallizing a protein is influenced by many parameters including the number of tested conditions and total trials (a somewhat personal limitation). The C-terminal boundary of all our crystallizing protein constructs is residue 174, and in those, we assume any flexible overhang would rather complicate crystallization.
Different from that, Q58I seems to need this exact overhang, which reaches to residue 180 indicating a stabilizing effect through helpful intra-domain interactions by residues 174-180. We now comment on that with a bit more emphasis in lines 414-416. This is fully supported by comparing NMR CSDs between WT and mutants, which show a unique involvement of this region for Q58I, but not the other mutants (Supplementary Fig. 2b). While a too short C-terminus would abrogate those contacts (leading to no crystal), the extended boundary in our Q58I crystal reveals residue E174 to have interactions to the NTD N-loop residue H59, as well as basic finger residues D103 and S105. Those contacts are not present (and apparently not needed) in the other structures (see below WT for comparison).
As an explanation, we suggest that any disruption of the mentioned core network (Q58-W108-F171) leads to protein instability (see also comments to the other reviewers and data on W108G and F171G) or the lack of crystals. From the three involved residues, only the mutation of Q58 still led to well-behaved protein and was crystallizable (although not at all straightforward). However, as shown below, this mutant is able to use E174 to reintroduce stability via the mentioned contacts, which obviously only appear accessible after breaking up the core network. Comparison of structures suggests, among others, that an interaction between the sidechains of H59 and Y172 is lost as a secondary effect of mutating the core network in Q58I. This enables interactions with E174, which altogether leads to a much more compact arrangement in this protein region.
Thus, we assume this is the reason for its altered crystallization behavior, while in all other mutants, residues beyond 173 may actually be obsolete for crystallization (or disturbing). In fact, e.g. the WT did not crystallize in the longer boundaries, and indeed here the thermal stability appears unaffected (see below). In contrast, the stabilizing effect provided by the extended C-terminus in Q58I is supported by a significantly increased thermal stability.
Likely, mutations of the other two network residues (W108 or F171) lead to a too drastic destabilization that cannot be compensated for by adjusted domain boundaries (crucial pi-interactions). At least we did not succeed in crystallizing F171G, while–even worse–for W108G NMR data reveal the lower degree of folding (together with both having significantly lower thermal stability).

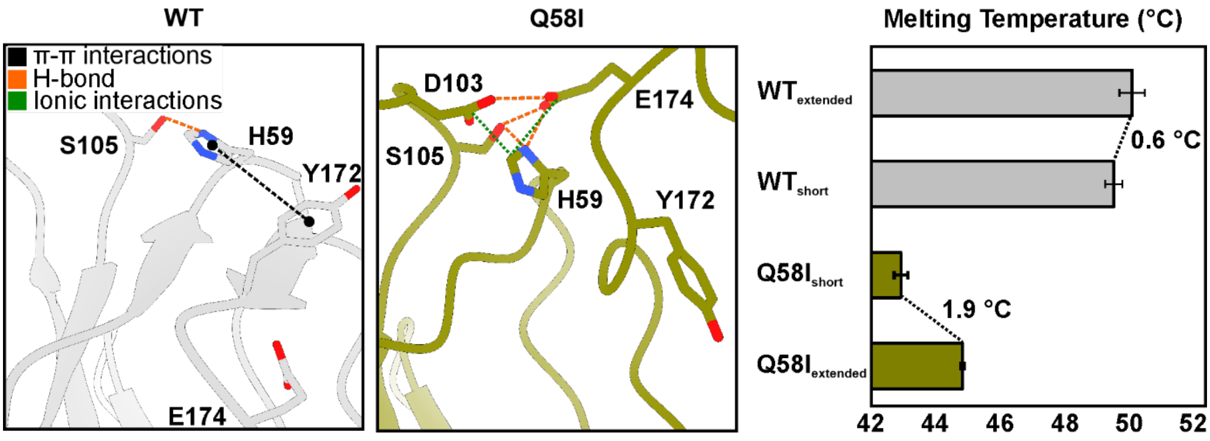

I think it would be interesting, if possible, to describe the lineage of the variants with the binding affinity. Like, were there less disruptive mutations longer sustained mutations detected in the human population as COVID evolved?

We apologize in case we do not correctly understand the question. However, note that we are only looking at single mutations in the conserved NTD. As any lineage of the variants contains additional mutations in other regions of N (i.e., CTD and IDRs), we can only speculate about if they correspond to binding affinity in individual lineages. We do not classify any of the natural mutants to be disruptive at the protein level as all respective crystal structures are WT-like. However, we certainly do not know the

possible effects of mutations on the RNA-genomic level or on the structure/function of the partly overlapping protein ORF9b. Certainly, changes in RNA structure and stability may heavily influence the virus' fitness. We have also briefly touched this very last point in the manuscript discussion again (from line 486 on).

Some other comments:

I do find myself wanting a little more from certain sections. I think the manuscript would be much stronger if the authors were able to get structures of N in complex with their RNA constructs, but I recognize that may be difficult / impractical -- although mention of if they attempted and any struggles, they may have faced would be welcome.

We agree that it would be of immense value to get a structure of the NTD in complex with target RNA as also mentioned in other places in this response letter. However, we–and others–have failed to provide an experimental, convincing structure, likely due to the dynamic nature of both the RNAs and the domain itself. Asking for the efforts within our lab, we have extensively tried to get a complex structure of the N NTD with RNA ligands. To name some of it here, we undertook e.g.

1. Co-crystallization trails of WT with both the preferentially bound Ext and the yet higher affine SL4ext RNAs at different temperatures (4°C, 20°C, 30°C and 37°C).
2. Co-crystallization using the increased-affinity natural NTD mutants D63G and P80R with Ext.
3. Soaking of apo protein crystals of WT / D63G / P80R with shorter fragments of RNA derived from Ext.

All of this has been tried in many crystallization conditions, but with no final success. In most cases, we end up with either micro crystals (smaller than 5 µm in size) or disordered quasi-crystals. In a few cases, we found non-definite RNA oligonucleotides as artifacts along with the NTD, but sticking to sites of the domain, that were inferred by crystal conditions and not supported by any other data.

We would again like to stress out that the challenge in obtaining NTD-RNA co-crystals is also reflected by the alternative approaches used in literature (staring from the valuable HADDOCK approach mentioned above) as well as e.g., an X-link MS-modelling approach by the Allain/Leitner team (PMID: 36928389, here with a folded element).

For this manuscript, we prefer not to elaborate on our manifold fails in crystallizing the NTD with relevant RNA. We do, however, underline again that we have undertaken strong efforts, which justifies the hypothesis of a crucial dynamic component for specific RNA-recognition as well as the chaperone-like nature of the NTD towards RNA. We have additionally added this statement now in the discussion, where we also list references to earlier attempts (line 462-463).

I find the conclusions the authors make are very much supported by their data, which is good. I more wish the authors took a few extra steps. The authors report D63G and P80R increase binding affinity of N for RNA and offer a structural explanation for it -- which I found agreeable. Are these mutations seen at all in sequence data from clinical populations? Why do the authors think SARS-CoV-2 hasn't evolved these mutations naturally? would there be some disadvantage associated with increasing RNA binding affinity through mutating these residues?

We agree that at some point we may have been short in linking our in vitro data to the viability and evolution of SARS-CoV-2. However, regarding the two mentioned mutants there may be a misunderstanding as they in fact are naturally occurring mutants. All natural mutants of our study were selected based on globally sequenced genome information through GISAID (PMID: 36841805, see Fig. 1), and we describe the criteria of how we chose mutants in dependence of sequence data and mutational prevalence in the methods section. Despite the rather minor benefit in RNA-binding there should be no disadvantages to the virus, and apparently the mutants were able to evolve.
However, this reviewer may be absolutely right: In fact, a too drastic increase of RNA-binding through the N NTD may be much more disadvantageous because it could affect the fine-tuned balance of RNA-encountering and -release during genome processing. Besides this, as already outlined above, effects of mutations may (additionally) involve the RNA-level, with outcomes not explainable on the level of the NTD protein domain. In the manuscript we have now included a short statement on the effects on the RNA level in paragraph 4 of the discussion.

I would have also liked to see more analysis by the authors on how the Q58I and / or S105I mutants contribute to RNA specificity. I found it interesting that these mutations disrupted the fold of the B-hairpin -- but the connection between the misfold and RNA specificity was lacking in my opinion. Again, without structures of RNA-bound N it is difficult to make more conclusions with the data the authors present, but more experiments pursuing this line of thought would have been appreciated and resulted in a much stronger work. Even perhaps a hypothesis of how mutations in this region disrupt specificity would be welcome. Especially since one mutation (Q58I) completely disrupts the core network while the other (S105I) does not, yet they have similar binding affinities for SL4 compared to the WT. This was, in my opinion, the weakest part of the work. Discussion or analysis on how these mutations might affect N oligomerization / packing would have been welcomed, but I recognize it's beyond the scope of the authors current intents.

With the above-mentioned lack of meaningful experimental structures, we can only provide a partial structural explanation regarding the impact of reduced specific binding by Q58I and S105I. Experimental evidence confirms that NTD mutants, like WT NTD (in the isolated domain context outside of full-length N) are monomeric (data not included in the manuscript), thus we do not expect that oligomerization of full-length N would be altered in the mutant context. Of note, the two des_mutants are non-natural, and their effect on fl-N can at this point only be theoretically discussed. It will certainly be of great interest to study these mutations in the context of genome packaging, as a tool to understand better the underlying mechanisms of how that important process is steered. From our data we conclude that bulk RNA binding can be accommodated in both mutants by the positive charge clustering on the primary RNA interaction surface, in combination with the central Y109, allowing for stacking interactions with RNAs. That interface is not altered by mutation of the core (or extended) network, still allowing the domain to interact with RNA using this canonical mechanism of encountering RNA. The formation of specific complexes together with preferred target RNAs, however, requires the interplay of the NTD's flexible fingers to 'grasp' the respective target. That concerted mechanism likely requires the network, e.g. in order to timely coordinate the set of protein sidechain contacts with respective RNA bases (or backbone found in the correct sequence-encoded conformation). This hypothesis is well reflected in the unaffected binding of S105I and Q58I to the non-target RNA SL4, while the affinity of S105I and Q58I to target RNA Ext is significantly reduced compared to WT. We now discuss our hypothesis a bit more detailed from line 530 onwards.

All that said, I do think the work is an extremely valuable contribution to the field and is very robust and thorough.

We thank the reviewer again for the constructive feedback and positive evaluation of our work and hope to meet the addressed points to satisfaction.

Reviewer #2 (Remarks to the Author):
A lot of attention was given to every mutation in the Spike protein in every SARS-CoV-2 variant of concern. However, the N protein, probably the second most important coronaviral protein (for instance, all antigen tests are based on the N protein) was never analyzed in such detail and, finally, Dhamotharan and Korn et al. fill the gap. This study represents careful analysis of the naturally occurring mutations in the N-protein and given the socioeconomic importance of CoV-2, it deserves to be published in Nat. Comm. I have few suggestions for improvement.

1) Dhamotharan et al. write that "The N-terminal domain (NTD) was suggested to mediate specific RNA-interactions, but the lack of high-resolution structures with viral RNA prohibits insights into the precise mechanism of complex formation."
An NMR structure with ssRNA and dsRNA was published and Dhamotharan et al. cite that paper. So this sentence, perhaps, should be reworded.

We apologize if this has been misleading or was misunderstood. What we wanted to say is that the NTD in complex with RNAs as shown by Dinesh et al. represents hybrid atomic models (PDB 7ACT and 7ACS). It is an NMR structure of the NTD used with both single and double stranded RNAs docked to it (using HADDOCK and YASRA) guided by NMR-observed CSPs, which may include indirect effects. The available structural ensembles therefore are definitely of large value (esp. early in 2020, but also now) and likely give a good sense of the respective interfaces (with many features in line with our data), which is why they appear as a starting and anchor point reflected in their multiple citations here. Still, we certainly cannot fully rely on them as compared to comprehensively validated experimental structures that would yield a direct readout of RNA-protein contacts obtained without bias (e.g. using inter-molecular NOEs).
In a different approach, the high resolution experimental complex structure of the NTD with a random double stranded RNA (PDB 7XWZ) does not show any specific contacts other than interactions to the RNA backbone and likely is not free of crystallographic artifacts. We indicate and cite these two approaches towards NTD-RNA complexes in paragraph 5 of the introduction. However, we still face a lack of high-resolution experimental structures with biologically relevant RNAs bound to the NTD RNA-binding interface found in solution. As a consequence, we would like to keep our statement unchanged, while we again emphasize the great value of any published NTD-RNA complex model.

2) In Figure 2 all the mutants are analyzed using gel shift assay. But the gels are not quantified, and the Kd was determined only for half of the mutants. The Kd needs to be determined for all mutants analyzed.

We apologize in case our experimental intention has not become clear here. Our EMSA experimental setup does not allow reliable quantification, as the RNA concentrations used are above the expected K_D (as derived by ITC – Korn et al., 2023, Nature Communications) for the WT, and apparently most of the mutants. In fact, this leads to an early saturation and/or steep rise-up in the binding curve (see our exemplary quantification of an EMSA showing WT NTD binding to SL4ext in a similar response to reviewer 1) and a large uncertainty in defining the transition (large error in K_D). Irrespective of that, EMSAs are a straight-forward approach to qualitatively estimate and compare affinities visually, e.g. as an initial experiment before aiming at quantification where needed.
This is exactly, what we intended to and thus all natural mutants were tested qualitatively for binding to the described target RNA SL4ext by EMSAs. Subsequently, affinities were quantitatively determined by MST only for those mutants that had shown altered RNA binding in EMSAs, while the natural mutants P67S, A119S, E136D and P151S showed RNA-binding comparable to WT. However from the latter, P67S was chosen as a representative mutant for quantification by MST in order to corroborate the experimental link between EMSA and MST for WT-like binders. This is now included in the adjusted Fig. 2d and supports the optically inspected affinities linked to reliable numbers from MST. Other than EMSAs, MST allows to reveal the subtle differences between the classes of binders because of small errors. However, altogether seeing the relatively high financial and laboratory load for repetitive MST experiments, we are thus convinced, that our logics is justified and only "relevant" NTDmut-RNA complexes remain quantified, after all of them were pre-analyzed by semi-quantitative EMSAs.

3) Binding affinities to the RNA Ext are presented for the mutants and wt in Fig. 3. It would be very informative to also use control RNA, such as scrambled RNA Ext.

We fully agree that including a bona fide non-related, random RNA negative control would be ideal in this and similar studies. However, existing literature demonstrates that the SARS-CoV-2 N NTD has the ability to interact with virtually any nucleic acid, including DNA. This observation motivated us to investigate potential markers that could help differentiate between preferred and non-preferred RNA elements within the viral genome in our recent work (Korn et al., 2023, Nature Communications). In that study, we tested a variety of artificial (non)-viral RNA sequences, all of which were bound by the NTD, though with varying patterns and affinities that followed a systematic model of complex formation with the NTD.

Given these findings, we expect any scrambled RNA to also induce CSPs in NMR and show a diffuse binding pattern in EMSAs. In this manuscript, we have therefore opted to use SL4 RNA, which has been established as a non-preferred/unspecific binder, in contrast to specific binding to the specific Ext RNA target.

4) "Mutations within this interface impact NTD RNA-binding, while no structural explanation for this has been provided yet."
Not really, the NMR structure of the NTD in complex with RNA can explain the impact of most of these mutations.
PDB id 7ACT actually shows that the residue Y109 makes a hydrogen bond with the RNA backbone. The mutant Y109A obviously cannot.

We apologize that this sentence may have been misleading in its intended meaning. We did not want to question the value of the PDB 7ACT as a hybrid NMR-docking model for our study (see comment above). Indeed, the very same structure well suggests the role of Y109, however based on a (thorough) docking approach.
We here meant to emphasize that no NTD interface mutant structures are available, which may reveal crucial effects of amino acid replacements relative to the WT, also apart from the exact interface, as e.g., other amino acids could take over the function of Y109. We have thus rephrased the sentence to make it hopefully clearer:
'Mutations within this interface impact NTD RNA-binding, while no experimental structure of such a mutant has been provided yet, which could report on the holistic effects from exchange of critical amino acids.'

5) "...the three-dimensional fold of the NTD depends on the conserved triple residue network (Q58-W108-F171), connecting the beta sheet core with the adjacent N- and C-terminal fingers."
If this would be true, than these residues would be absolutely conserved. But they are not conserved between alpha and beta coronaviruses and yet the N NTD appears to have the same fold in alpha coronaviruses. For instance, PDB id 5N4K. Although later in the manuscript, the differences between alpha- and beta- coronavirus NTDs are discussed, the statement above seems too strong. Should be at least rewritten as "...the three-dimensional fold of the NTD of Betacoronaviruses depends on the conserved triple residue network (Q58-W108-F171), connecting the beta sheet core with the adjacent N- and C-terminal fingers."

We appreciate this suggestion and have now changed the text exactly as suggested.

Reviewer #3 (Remarks to the Author):
Among them, R203(K/M) and G204R, located in the SR-rich region, are most prevalent and associated with an increased viral load and fitness12–14.
However, a comprehensive understanding of the NTD RNA-binding mechanism is still missing, despite some efforts of gaining structural information on RNP.
Interestingly, though widely used as a biological tool to mute NTD RNA-binding in a fl-N context23,28, no structural characterization of this des_mutant existed. We here solved the Y109A crystal structure and can show that structural integrity is retained in this mutant.
Analysis of the CSP plots for each residue in the ds-14mer titration with this distinction in mind reveals a cluster of residues around D81 and I146, including the helix at residues 79–82, residues 119 and 120, and residues 143–146 (Fig. 4h, Fig. S7). As the CSPs at this site differ substantially from those near the primary binding site, we conclude that this shift corresponds to a second, weak binding surface of the NTD.
Fig 2: a. P80R no colors on the structure, b. is covering structures in e,

We assumed this section constitutes a part of the reviewer's draft, which was accidentally not removed, and as such we shaded that section.

The manuscript by Dhamotharan et al is focused on elucidating the effect of naturally occurring and designed mutations on the structure of the NTD of SARS-CoV2 and on identifying the structural features that contribute to specific interactions with RNA. In the process they determined crystal structures of 9 mutants, their effect on CSP by NMR, and compared their thermal stability and RNA binding affinities to WT. Naturally occurring mutations were chosen based on their prevalence in variants of concern. Designed mutants were chosen based on earlier reports of loss of binding to RNA and from examining long range contacts in the crystal structure, and their presence at the RNA primary site and based on conservation among alpha and beta coronaviruses. The work is solid, thorough, uses both NMR and crystallography and multiple other biophysical tools on a large number of mutants. Data are well presented, and the manuscript is clearly written. However, much of the results are expected with no major insights provided as described below.

1. The 6 naturally occurring mutant NTD showed similar fold to the WT and similar binding affinity to RNA, except for two that showed higher affinity due to changes in their electrostatic contributions. This result is expected. It is rare in proteins that a single mutation will change the overall fold and small changes in affinity are rarely associated with changes in structure. The NT structure has been extensively studied. Structures of the NTD have been determined by multiple techniques (solid state NMR, solution NMR, crystallography) and therefore methods for purification and crystallization are available making this investigation pretty straightforward.

While we agree that a single point mutation is not automatically expected to severely impair a domain's fold, the real impact of such a mutation should not be assumed impactless unless experimentally proven. In fact, single mutations can lead to domain instability or unfolding, albeit this may not be evolutionarily beneficial. However, also small local steric or charge changes can have drastic effects on structural integrity, as we show by our single-residue mutants S105I and Q58I.

Indeed, the WT NTD structure has been studied by various groups, as reflected by numerous X-ray structures. Yet, no structural information existed for NTD mutations. We thus believe to contribute significantly to the field by providing a comprehensive set of structures, which create an atomistic context of those mutations.
While the experimental setup for WT crystallization is widely available, it does not translate 1:1 to the mutants. For some of the mutants, a lot of effort went into optimization of crystallization conditions (Supplementary Table 2), and of note: not every mutant could be crystallized (despite great efforts) – see also comment below (regarding the question of F171G crystallization). Also, we found that e.g., the W108G mutant led to a significantly more complicated protein sample in terms of aggregation and misfolding, representing a single mutation with much impact and a not-so-straightforward-to-adjust purification.
In fact, both W108G and F171G were unstable proteins that could not be concentrated easily for crystallization trails. Supplementary Fig. 9 shows HSQC overlays of these two mutants and the significant effects the mutations have on the NTD fold as seen by significantly less well dispersed peaks in W108G (unfolding, oligomerization). Thermal melting temperatures of W108G and F171G are

approximately two-fold lower compared to that of WT. We thus respectfully disagree in that any version of the NTD is per se straightforward to produce and investigate.

2. For these same mutants, the CSPs were different but not much was made of what this observation implies.

We apologize if we have not outlined this carefully or clearly enough. In our initial screening, we clustered mutants with regard to 'local' and more 'wide range' effects, which we did by analyzing the chemical shift differences (CSDs) observed for mutants compared to WT (by HSQC spectra). The categorization based on CSDs was well in line with the structural and biochemical analysis (superimposing crystal structures/RNA-binding affinity). But we agree that we did not revisit the initial categorization during the manuscript. To undo that shortcoming, we added a potential link between CSDs and RNA-binding in the first paragraphs of the first two result sections, i.e.

"The three-dimensional fold of the NTD is conserved in naturally occurring mutations" and "NTD natural mutants with increased RNA-binding affinity".

3. Designed mutants chosen at the RNA primary site reduce RNA binding like Y109A and R107A but those have been reported earlier. NMR characterization of structure and dynamics and RNA binding are published for Y109A (Estelle et al., PNAS Nexus, 2023). What is new here is the crystal structure, but the conclusion is the same as in Estelle et al, that there is no change in structure and that there is some weakened binding rather than total loss of binding.

In line with Estelle et al (as cited in the submitted manuscript at line 276), we show that Y109A is structurally intact, and its RNA-binding is in fact reduced due to the indicated mutation. The structural confirmation has been missing and nicely confirms previous reports, but importantly also raises awareness that it does not abolish binding to RNA completely. We have now again mentioned the supportive observation of Estelle et al in our discussion.

4. The most interesting part of this manuscript is in the third set of mutants referred as intradomain contact mutants, and their effect on the structure and selectivity of RNA recognition. These mutants drastically affected the fold with the highest RMSD compared to WT and therefore their effect on binding is expected. However, what is interesting here is identifying the protein residues that contribute to the difference in specificity of binding between the two RNA segments studies SL4 and SL4-Ext. Still some lingering questions should be addressed as below:

a. Why weren't the SL4, and SL4-Ext tested with the naturally occurring mutants or the Y109A?

We agree that this is a valuable experiment. We now included NMR observed titrations (**Supplementary Fig. 4a**) with Ext and SL4 RNAs as CSP plots in the Supplementary Information for the naturally occurring affinity-altered mutants D63G and P80R, as well as the designed mutants Q58I, S105I (**Supplementary Fig. 11a**) and Y109A (**Supplementary Fig. 5b**). Additionally, P67S was included as an exemplary mutant to again underline the WT-like binding behavior of this category of mutants (as expected from the structure). Individual effects for mutants are now included in the manuscript text.

b. Why wasn't a structure for F171G determined, while there are structures for the two other mutants in the network.

Of course we had also wanted to include this particular structure. Despite great effort we were not able to crystallize F171G (reflecting that crystallization of mutant NTDs was not straight-forward for all of them). Both F171G and W108G have significantly lowered thermal stability (Fig. 4 and Supplementary Fig. 7), and thus, F171 and W108 seem to be more critical for structural integrity than Q58. We assume this correlates with the lack of success in crystallizing F171G.

c. What is the rationale for mutations to G and to I. While it was clear why these specific residues were mutated, it was not clear why to G and I.

The rational for mutation to Isoleucine was to maintain the residue sidechain dimensions, yet to eliminate charge and the ability for H-bonding as would be given in Q58I (line 351-352). S105I has

previously been described to have an impaired ability to distinguish RNA targets (Korn et al., 2023, Nature Communications).
The choice for mutations to G was rationalized by reducing its capacity to make contacts as strictly as possible. We have added a sentence in the respective paragraph (line 339) and have included a specific paragraph in the methods section (**construct design**).

d. There is no discussion of why Ext is a better binder, is it because of nucleotide specificity or because of its single stranded structure. If the latter then this supports the observation reported in Estelle et al, 2023 of higher specificity to single stranded RNA over double stranded. There was no mention about this in the manuscript.

The choice of target vs. random (viral) RNA was based on our previous publication (Korn et al., 2023, Nature Communications), where we studied the RNA-binding preferences of the N-NTD in great detail. In line with Estelle et al 2023 and our own publication, we claim that the NTD has a preference for labile folded RNA stems that exist as ssRNA at physiological temperatures. Thus, Ext is preferred, based on its single stranded nature, but importantly, its genomic context, locating it adjacent to a stable stem-looped structure (SL4), marking the target nature of this sequence (seen by increased affinity of NTD for SL4ext compared to only Ext, see Korn et al., 2023, Nature Communications).
To again emphasize the independent observations (strengthening the relevance of both of them), we have now again added a more direct reference to this in the introduction and discussion and introduced the used target RNA more in detail as follows:

'SL4ext is a described cis-regulatory element located in the 5'-untranslated region of the genomic RNA, comprising the stable stemloop (SL) 4 ***[Voegele et al., 2023]*** *and a 22 nucleotide extension (ext), that transiently folds as a SL at physiological temperature. We recently showed that the NTD preferentially binds to single stranded Ext* ***[Korn et al., 2023]****, in line with its described preference for ss over dsRNA* ***[Dinesh et al., 2020; Estelle et al., 2023]****.'*

We thank all reviewers for their thoughtful review of our revised manuscript and greatly appreciate their valuable feedback.

REVIEWERS' COMMENTS

Reviewer #1 (Remarks to the Author):

I want to thank the authors for thoroughly addressing each and every comment (from all reviewers). I only have one minor comment:

Figure 3D -- just color that alanine differently so it is easier to see.

We are pleased to have addressed the comments and concerns raised by Reviewers 1 and 3. In addition, we have adjusted Figure 3, panel d, for improved clarity.

Reviewer #2 (Remarks to the Author):

The manuscript by Dhamotharan and Korn significantly improved during revisions and can be published as it is although I have few suggestions below. Please, treat these as only suggestions, I do not want to delay the publication process any further. Up to the authors and the editor if they use these.

We appreciate the positive reception.

1) In the meantime, a high-resolution structure of the NTD bound to DNA aptamer was published by Esler et al in NAR. This could be perhaps discussed or at least mentioned.

We thank the reviewer for highlighting this and now cite the publication in our discussion.

2) The N-terminal domain (NTD) was suggested to mediate specific RNA-interactions, but the lack of high-resolution structures with viral RNA prohibits insights into the precise mechanism of complex formation.” I still believe that this sentence is misleading, it overestimates novelty of this study. It could perhaps state: "The N-terminal domain (NTD) was suggested to mediate specific RNA-interactions, but the lack of high-resolution structures with viral RNA prohibits insights into the precise mechanism of complex formation although some information was gained from hybrid structures of the NTD in complex with ss and dsRNA.”

We have incorporated the suggested wording to make the sentence in the abstract more concise and to better acknowledge existing literature.

Reviewer #4 (Remarks to the Author):

I co-reviewed this manuscript with one of the reviewers who provided the listed reports. This is part of the NatureCommunications initiative to facilitate training in peer review and to provide appropriate recognition for Early Career Researchers who co-review manuscripts.

Reviewer #5 (Remarks to the Author):

I co-reviewed this manuscript with one of the reviewers who provided the listed reports. This is part of the NatureCommunications initiative to facilitate training in peer review and to provide appropriate recognition for Early Career Researchers who co-review manuscripts.

EDITORIAL REQUEST

Having spoken to the reviewers, they still raise some concerns regarding the rationale for not completing MST experiments for all the mutants developed in this work, especially as the gel-based assays have not been quantified. Editorially, we agree with this concern and kindly suggest that this additional work is undertaken to expand the appeal to the reader and level of insight drawn in this work. We do, however, request that gel quantification is undertaken before resubmission.

We have included gel quantifications in the Source Data file alongside the EMSA replicates. We hope that incorporating the gel quantifications satisfactorily addresses the concern raised. We conducted EMSAs as an initial tool to classify mutants (alongside with NMR-observed changes of peak patterns). We realised early that the EMSAs do not qualify to derive reliable K_D values for the mutants (close in range), as errors and deviations are relatively big. While 'by eye' inspection indicates comparable binding patterns for the WT and four of the nat_mutants, we observed sharper complex bands for the two nat_mutants P80R and D63G. Based on that, we expected the K_D values of these two mutants to differ from that of the WT. We consequently chose those two mutants, the WT and P67S (as a representative for the four nat_mutants with unsuspicious binding pattern) to do a quantitative determination of K_D values by MST. We also provide values for the binding-impaired Y109A and R107A mutants.
